# Keys to the avian *Haemoproteus* parasites (Haemosporida, Haemoproteidae)

**DOI:** 10.1186/s12936-022-04235-1

**Published:** 2022-09-19

**Authors:** Gediminas Valkiūnas, Tatjana A. Iezhova

**Affiliations:** Nature Research Centre, Akademijos 2, 2100, LT-08412 Vilnius, Lithuania

**Keywords:** Key to species, Birds, Molecular characterization, *Haemoproteus* taxonomy, *Plasmodium*

## Abstract

**Background:**

*Haemoproteus* is a sister genus to malaria parasites (*Plasmodium*), which both belong to the order Haemosporida (Apicomplexa). Parasites of both genera are flourishing in birds, however, *Haemoproteus* species are noticeably less investigated. This is unfortunate because knowledge about close relatives of malaria pathogens is important for better understanding the evolutionary origin and basic biological features of the entire group of haemosporidian infections. Moreover, recent findings show that *Haemoproteu*s species can cause severe damage of various bird organs due to megalomeronts and other exo-erythrocytic stages. These haemosporidians are remarkably diverse, but remain neglected partly due to difficulties in species identification. Hundreds of *Haemoproteus* genetic lineages have been reported in birds, and numerous new lineages are found each year, but most remain unidentified to the species level. Numerous new *Haemoproteus* pathogens were described during the past 20 years. However, keys for their identification are absent. Identification of *Haemoproteus* species remains a difficult task and is an obstacle for better understanding of the distribution and epidemiology of these parasites. This study aimed to develop comprehensive keys for the identification of described avian *Haemoproteus* species using morphological features of their blood stages (gametocytes).

**Methods:**

Type and voucher preparations of avian *Haemoproteus* species were accessed in museums in Europe, Australia and the USA. Gametocytes of most described species were examined, and these data formed a background for this study. The data also were considered from published articles containing parasite species descriptions. The method of dichotomous keys was applied. The most difficult steps in the keys were accompanied with references to the corresponding parasite pictures.

**Results:**

In all, 201 published articles were included in this review. Morphological diagnostic features of gametocytes of all described *Haemoproteus* species were analysed and compared. Illustrated keys for identification of these parasite species were developed. Available information about the molecular characterization of *Haemoproteus* parasites was provided.

**Conclusion:**

This review shows that 177 described species of avian *Haemoproteus* can be distinguished and identified in blood films using morphological characters of their gametocytes and host cells. These species were incorporated in the keys. Information about possible morphologically cryptic parasites was provided. Molecular markers are available for only 42% of the described *Haemoproteus* parasites, calling for researchers to fill this gap.

## Background

Order Haemosporida (Alveolata, Apicomplexa, Sporozoa) unites a diverse group of closely related obligate heteroxenous protists, whose currently are classified in four families—Plasmodiidae, Haemoproteidae, Leucocytozoidae and Garniidae [1–5]. These pathogens have many similar basic features in their life cycles. Mainly, they are transmitted exclusively by dipteran blood-sucking insect (Diptera), in which the sporozoites (invasive stage for vertebrates) develop. Sporozoites initiate the exo-erythrocytic development (exo-erythrocytic merogony or schizogony) in various tissues of vertebrate hosts. Exoerythrocytic merozoites are capable of infecting blood cells, in which gametocytes (the invasive stage for vectors) finally inhabit. Asexual dividing stages (erythrocytic meronts or schizonts) do not develop in *Haemoproteus* species. Gametocytes are characterized by sexually dimorphic characters. Development in vectors is similar in all haemosporidians; it consists of exflagellation, which is followed by the sexual process of the oogamy, development of motile ookinetes, sporogony, and finally the formation of sporozoites, which complete their maturation in the salivary glands of vectors. Infection of vertebrate hosts occurs actively during the blood meal of vectors, who inject sporozoites into the blood stream. Similarities in life cycles and other features of the biology (particularly ultrastructure) of haemosporidians belonging to different families [3, 5–7] have been supported by molecular phylogenies, which indicate that these parasites are relatives and likely have a common origin [8–12]. In other word, accumulation of new knowledge about different groups of haemosporidians, which are remarkably diverse and flourishing in wildlife [5, 6, 13–15], would be beneficial for better understanding the biology of the entire group of Haemosporida, including agents of diseases of domestic animals and humans, as well as malaria [10].

Molecular phylogenetic data show that *Haemoproteus* parasites (Haemoproteidae) are a sister group to malaria agents of the genus *Plasmodium* (Plasmodiidae) [8, 9, 11]. Haemosporidians of both these genera do not digest haemoglobin completely, resulting in the accumulation of residual pigment (haemozoin) in their blood stages [1–3]. This feature unites species of *Haemoproteus* and *Plasmodium* and distinguishes them from species of the Leucocytozoidae and Garniidae, which do not produce residual pigment when developing in red blood cells. The life cycles of *Haemoproteus* and *Plasmodium* parasites differ mainly due to the inability of the haemoproteids (i) to multiply in blood cells (erythrocytic merogony is absent) and (ii) to complete sporogony in mosquitoes [7]. *Haemoproteus* parasites are transmitted mainly by *Culicoides* biting midges (Ceratopogonidae), and a few species are vectored by louse flies (Hippoboscidae) [2, 7, 16]. In spite of these differences, the knowledge on *Haemoproteus* spp. is important for better understanding evolutionary biology of haemosporidians [17–19], including human malaria parasites of genus *Plasmodium* [10]. For example, the application of *Haemoproteus tartakovskyi* genomic information in phylogenetic studies contributes to understanding the evolutionary relationships of *Laverania* parasites [8].

Haemosporidians of the genus *Haemoproteus* parasitize only birds and reptiles [1, 3, 6]. These protists remain a neglected group of blood pathogens mainly because they have been traditionally considered to be relatively benign to their hosts [20]. This seems to be true in regard to the blood pathology during haemoproteosis due to the absence of multiplication in blood cells and predominantly light or moderate parasitaemia, which rarely reaches 5%, but usually is less than 1% in wild-caught animals [3]. However, recent studies show that haemoproteids are pathogenic to blood-sucking insects, including mosquitoes, who often die within 12 h after taking heavily infected blood meals due to damage caused by the migration of ookinetes [21–23]. Furthermore, the application of molecular diagnostic tools has proven that large-sized megalomeronts (up to 300 µm and even bigger) develop in many *Haemoproteus* infections, resulting in the damage of various organs [2, 24–30]. These findings call for research aimed at better understanding the biology of haemoproteids in regard of animal health.

*Haemoproteus* species are cosmopolitan and often prevalent in birds [3, 5, 14, 31–34]. Molecular studies have revealed over 4600 unique cytochrome *b* gene lineages of these parasites, and many more likely exist [13] (see MalAvi database http://130.235.244.92/Malavi, accessed in April 2022). Over 170 species of *Haemoproteus* have been described, including 49 new species described during the past 20 years. Difficulties in species identification using morphological data preclude comprehensive parasite diversity research. This is unfortunate because morphological and molecular data complement each other and are essential in obtaining a true understanding of pathogen diversity in certain bird populations [35, 36], particularly during co-infections of parasites belonging to same genus [37–39]. Species-specific molecular markers are absent for the majority of avian *Haemoproteus*, and currently they are difficult to design and use due to the vast genetic diversity of these pathogens, most of which remain insufficiently investigated or even non-described in wildlife. Morphological identification using microscopic examination of blood films supplements the information providing by polymerase chain reaction (PCR)-based diagnostic tools and remains important in the research of wildlife haemosporidians [36, 38, 40]. Thus, the available keys for the identification of avian *Haemoproteus* species [3] should be reworked and supplemented.

This review aimed to simplify the identification of avian *Haemoproteus* species by developing easy-to-use keys based on morphological characters of gametocytes, the parasite development stage easily accessed due to presence in the peripheral circulation. This article provides comprehensive keys, which include new *Haemoproteus* species that are not already found in formerly published keys [3]. This should assist academic and veterinary medicine researchers in the identification of *Haemoproteus* pathogens. The available information about molecular markers (molecular barcodes), which can be used for the detection of described *Haemoproteus* species and the comparative research, was summarized as well. This study generalizes the over 40-years of experience of the authors in taxonomy of *Haemoproteus* species, and is designed for researchers who are interested in wildlife pathogens.

## Methods

Full-length papers with descriptions of new *Haemoproteus* species as well as articles with re-descriptions and molecular characterizations of these parasites published in peer-reviewed journals were considered. In all, 201 articles and books were reviewed, and 191 publications containing the representative morphological and/or PCR-based information related to identification of these parasites were cited and incorporated in the References.

Type and voucher preparation as well as gametocyte images of avian *Haemoproteus* parasites were obtained from the collections of Nature Research Centre (Vilnius, Lithuania), International Reference Centre for Avian Haematozoa (Queensland Museum, Queensland, Australia), the US National Parasite Collection (National Museum of Natural History, Washington DC, USA), Natural History Museum (London, UK), Muséum National d’Histoire Naturelle (Paris, France), Grupo de Estudio Relación Parásito Hospedero, Universidad Nacional de Colombia (Bogotá, Colombia) and individual researchers. All accessed preparations were examined. An Olympus BX61 light microscope (Olympus, Tokyo, Japan) equipped with an Olympus DP70 digital camera and imaging software AnalySIS FIVE (Olympus Soft Imaging Solution GmbH, Münster, Germany) was used to examine preparations and prepare illustrations. It is important to note that the staining quality of some old type specimens of *Haemoproteus* species, which were obtained from museums, was of insufficient quality mainly due to fading, resulting in some poorly visible diagnostic characters. However, images of the parasites from such preparations were included in this review and were shown in the corresponding species figures if they provided valuable information about the general shape of gametocytes, the size of pigment granules, the mode of influence on host cells and others readily visible taxonomic characters. Use of this information helps to distinguish some species, and also is important for future taxonomic studies being the source of illustrations from valuable type specimens. The quality of these slides could hardly be improved in the future, so worth documentation at this stage. Black and white drawings were also provided to illustrate morphological details of the parasites, of whose high-quality photographs were not available.

Classical dichotomous keys were developed for the identification of *Haemoproteus* species [3]. Each key consists of steps divided into two alternatives, which identifies the species of a specimen following a series of simple choices that lead the user to the correct name of a given species. The most difficult choices, which might lead to ambiguity, were accompanied with references to the corresponding pictures that further illustrate meaning of the text information. This simplifies the comparison of diagnostic features used in the keys and minimizes possible misunderstanding. All species names in the keys were accompanied with references to the original parasite descriptions, re-descriptions and (or) other publications, which contain description and (or) illustrations of corresponding parasites. Published articles containing valuable morphological descriptions were collected, analysed and cited. These references help to access parasite descriptions and confirm a parasite identification. Information about the barcoding DNA sequences, which can be used for molecular detection and identification of corresponding parasites are also provided.

The experimental observations showed that *Haemoproteus* species vary in vertebrate host specificity, but the same parasite usually cannot complete life cycle and produce invasive stages (gametocytes) in birds belonging to different orders (see review in [3, p. 69]. Molecular sequence information is in accordance with these empirical data and indicates only rare cases when the same *Haemoproteus* lineages could be found in birds belonging to different orders [13]. Importantly, the rare reports of the same *Haemoproteus* lineages in birds of different orders have never been supported by the observation of the corresponding species gametocytes, an invasive parasite stage for vectors, indicating abortive (incomplete) development, which is a dead-end of infection [7]. Thus, morphologically similar parasites in birds belonging to different orders are different species in most cases. This conclusion was confirmed by molecular data [7, 9, 11, 13]. Due to vertebrate host specificity and the resulting restriction of parasite distributions by bird orders, the natural host range of haemosporidians remains helpful in species identification. This provided an opportunity to design separate keys for identification of parasites inhabiting birds of different orders. This approach simplifies parasite identification by minimizing the number of species, which are needed for comparison before making the final conclusion about a species identity. This approach was used in the keys.

Approximately 50% of all described *Haemoproteus* species parasitize birds of the order Passeriformes. Due to the marked species diversity of haemoproteids in passerines, the keys for their identification were developed for closely related passeriform bird families, which were grouped in suborders or superfamilies as suggested by the current bird phylogenies [41, 42]. Such keys provide the opportunity to easily identify the majority of described parasites of passeriform birds. However, it should be noted that some *Haemoproteus* parasites of passerines might infect and produce gametocytes in birds belonging to different families within the same order [43], supporting information that host taxonomic characteristic cannot be considered as the main parasite taxonomic character [3, 44]. This might lead to circumstances when a sample under identification could be not found in a certain key. This also certainly will happen if a researcher is dealing with a new (non-described) parasite species. Further comparison of such samples with parasites of most closely related avian groups is suggested before making a final conclusion about the identity of a sample. This is a relatively weak point of the keys for the identification of *Haemoproteus* species parasitizing passeriform birds. However, experience shows [3] that creating one big key for all parasites of Passeriformes birds would be even more difficult to use due to the need of too many minor morphological characters, which often are difficult to estimate in practical work, particularly during low parasitaemia. This usually make the identification even more complicated in comparison to the approach, which was used here.

Genus *Haemoproteus* includes two subgenera—*Haemoproteus* and *Parahaemoproteus*. Species of these subgenera differ in patterns of sporogony, which occur in louse flies (family Hippoboscidae) and *Culicoides* biting midges (family Ceratopogonidae), respectively [3, 7]. *Haemoproteus* and *Parahaemoproteus* parasites are indistinguishable at the gametocyte stages, but might be present in birds of the same orders. They also sometimes occur in co-infection [3]. The current examples are the parasites of Columbiformes and Suliformes birds [45, 46]. To facilitate identification of species of both subgenera using morphological characters of gametocytes, these parasites were given in the same keys. Most of avian haemoproteid species belong to *Parahaemoproteus*. All species of subgenus *Haemoproteus* were indicated in notes to the corresponding keys.

## Results

**Fig. 1:**
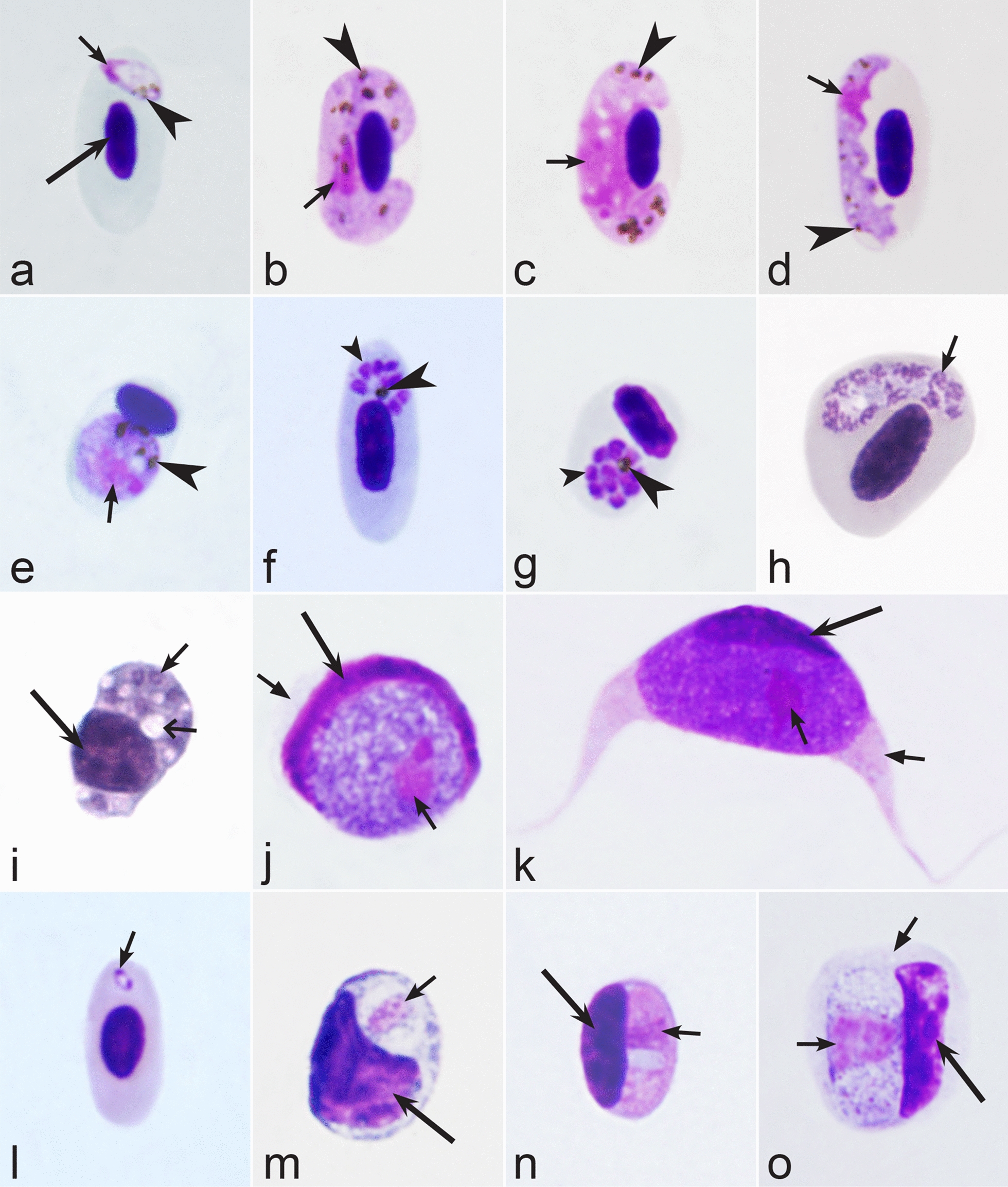
Main morphological features of blood stages, which are helpful to distinguish *Haemoproteus* parasites (**a**–**c**) from other avian intracellular protists (**d**–**o**). Young (**a**) and fully grown (**b**, **c**) gametocytes of *Haemoproteus* species. Fully grown gametocytes (**d, e**) and mature erythrocytic meronts (**f**, **g**) of *Plasmodium* species. Growing meronts of *Garnia* (**h**) and *Fallisia* (**i**) species. Gametocytes (**j**, **k**) in roundish host cell (**j**) and fusiform host cell (**k**) of *Leucocytozoon* species. Growing meront (**l**) of *Babesia* species. Merozoite (**m**) of *Isospora* species. Sporozoite (**n**) of *Lankesterella* and gamont (**o**) of *Hepatozoon* species. Note presence of malarial pigment (haemozoin) in species of *Haemoproteus* (**a**–**c**) and *Plasmodium* (**d**–**g**) and its absence in species of other avian blood parasites (**h**–**o**). Elongate gametocytes of malaria parasites belonging to the subgenera *Giovannolaia* and *Huffia* (**d**) are similar to gametocytes of *Haemoproteus* species in forms, but the gametocytes of malaria parasites usually are more irregular in shape (**d**) and the outline of their macrogametocyte nuclei often is not so well indistinct (compare **b** and **d**). Presence of merogony in blood cells (**f**, **g**) clearly shows malaria infection. Long simple arrows—host cell nuclei. Short simple arrows—parasite nuclei. Simple arrowheads—pigment granules. Simple small arrowheads—merozoites. Simple wide short arrows—vacuoles. Triangle arrows—remnants of host cell cytoplasm. Other explanations are given in the text

Birds are hosts of various intracellular blood parasites belonging to Apicomplexa. For example, they are infected by haemosporidians of the genera *Plasmodium, Leucocytozoon, Garnia, Fallisia* and related species of *Babesia, Isospora, Lankesterella* and *Hepatozoon* [2–4, 47, 48]. These organisms (Fig. 1a–o) often occur in co-infections. *Haemoproteus* species can be readily distinguished from all other avian intracellular haematozoa, except *Plasmodium* parasites, because their blood stages (gametocytes) develop exclusively in red blood cells and always contain refractive pigment granules (haemozoin granules) (Fig. 1a–c), which are absent in all other blood parasites (Fig. 1h–o). Even the smallest *Haemoproteus* gametocytes contain pigment granules and can be distinguished from other intracellular blood parasites (Fig. 1a), except for *Plasmodium* species, which sometimes look similar at the gametocyte or early trophozoite stages (Fig. 1d, e). Furthermore, it is important to note that gametocytes of some species of *Haemoproteus* are similar to the elongate gametocytes of malaria parasites (Fig. 1b–d). Species of *Plasmodium* can be readily distinguished because they multiply in blood cells and produce meronts (= schizonts), in which invasive merozoites develop (Fig. 1f, g). This is not the case in species of *Haemoproteus*. Extensive examination of blood films at magnification 500× usually provides opportunity to visualize meronts of *Plasmodium* even during low parasitaemia and thus to identify malaria infections. Examination of blood films using an oil immersion objective 50× is particularly convenient for this diagnostic procedure. However, an obstacle still might be a low *Plasmodium* sp. parasitaemia (< 0.001% of infected red blood cells), which however is the obstacle for identification of any haematozoan infection using microscopic examination of blood films and sometimes even sensitive PCR-based methods [38].

**Fig. 2:**
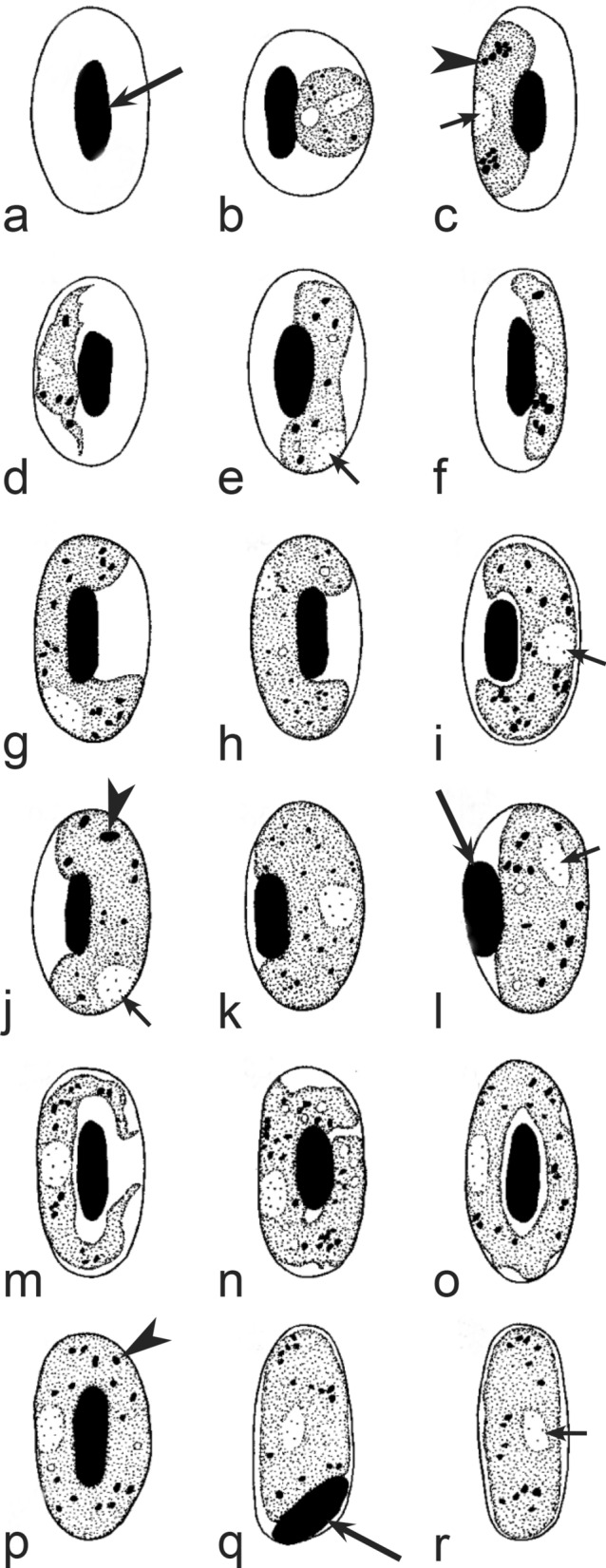
Main morphological forms of fully grown gametocytes, which are used in keys for identification of *Haemoproteus* species: roundish (**b**), microhalteridial (**c**–**f**), halteridial (**g**–**j**) and broadly-halteridial (**k**, **l**), close to circumnuclear (**m**, **n**), circumnuclear (**o**, **p**) and rhabdosomal (**q**, **r**). Uninfected erythrocyte (**a**) was shown for comparison purpose. All pictures show only fully grown gametocytes. Note that form of growing gametocytes often is different from the form of the fully grown gametocytes. That is why the form of young growing gametocytes usually was not mentioned in the keys, except for some rare cases, which were specified in the tables for species identification. The gametocyte forms depend on mode of parasite growth in red blood cells, which often is species-specific and was used in taxonomy. Various variations of halteridial (**c**–**l**) and circumnuclear (**m**–**p**) gametocyte forms predominate in avian haemoproteids. Roundish form (**a**) is particularly rare. Rhabdosomal forms (**q**, **r**) develop in several *Haemoproteus* species whose fully grown gametocytes enucleate infected erythrocytes. Dumbbell-like form of gametocytes (**e**) was often mentioned in the keys. Long simple arrows—host cell nuclei. Short simple arrows—parasite nuclei. Simple arrowheads—pigment granules. Other explanations are given in the text

It should be noted that a shape of fully grown gametocytes is an important character for many *Haemoproteus* species identifications [2, 3, 49]. Several major gametocyte forms are readily distinguishable and were used in the keys (Fig. 2a–r). These forms are microhalteridial (fully grown gametocytes are small and do not reach the poles of infected erythrocytes, Fig. 2b–f), halteridial (fully grown gametocytes reach and occupy the poles of infected erythrocytes, Fig. 2g–j), circumnuclear (fully grown gametocytes encircle the nuclei of infected erythrocytes completely, Fig. 2o, p) and rhabdosomal (fully grown gametocytes push the nuclei of erythrocytes to polar position and finally enucleate the host cells, Fig. 2q, r). A non-infected erythrocyte was shown in Fig. 2a for comparison with the infected erythrocytes. Sometimes subdivisions of these main forms were also helpful during species identification, and they were mentioned in the keys. These forms are broadly-halteridial (fully grown gametocytes occupy the poles of infected erythrocytes and markedly displace the nuclei laterally, Fig. 2k, l) and close to circumnuclear (fully grown gametocytes encircle the nuclei of infected erythrocytes nearly completely, Fig. 2m, n). It is important to remind in this regard that the form of only fully grown gametocytes was considered in the keys.

Blood films should be carefully examined before parasite identification. When the most typical growing and fully-grown gametocytes of *Haemoproteus* are recognized in samples, the next step is the species identification using the keys. The latter were designed in the form of separate tables, each created for a separate group of avian hosts. To facilitate the use of the keys, a guide to all keys has been provided in Table 1. It lists all the tables for *Haemoproteus* species identification in relation to bird orders or relative families, as grouped in the keys.

**Table 1:** Guide to keys of *Haemoproteus* species according to their avian hosts

Bird taxa^a^	Key for parasite species
Order Accipitriformes	Table 2, Fig. 3
Order Anseriformes	Table 3, Fig. 4
Order Apodiformes	Table 4, Fig. 5
Order Bucerotiformes	Table 5, Fig. 6
Order Caprimulgiformes	Table 6, Fig. 7
Order Cariamiformes	Table 7, Fig. 8
Order Cathartiformes	Table 8, Fig. 9
Order Charadriiformes	Table 9, Fig. 10
Order Ciconiiformes	Table 10, Fig. 11
Order Coliiformes	Table 11, Fig. 12
Order Columbiformes	Table 12, Fig. 13
Order Coraciiformes	Table 13, Fig. 14
Order Cuculiformes	Table 14, Fig. 15
Order Falconiformes	Table 15, Fig. 16
Order Galliformes	Table 16, Fig. 17
Order Gruiformes	Table 17, Fig. 18
Order Musophagiformes	Table 18, Fig. 19
Order Otidiformes	Table 19, Fig. 20
Order Pelecaniformes	Table 20, Fig. 21
Order Piciformes	Table 21, Fig. 22
Order Psittaciformes	Table 22, Fig. 23
Order Pterocliformes	Table 23, Fig. 24
Order Strigiformes	Table 24, Fig. 25
Order Suliformes	Table 25, Fig. 26
Order Passeriformes	
Suborder Tyranni	Table 26, Fig. 27
Suboder Passeri	
Families Meliphagidae, Oriolidae, Pachycephalidae, Vireonidae	Table 27, Fig. 28
Families Aegithinidae, Artamidae, Malaconotidae, Vangidae	Table 28, Fig. 29
Families Corvidae, Dicruridae, Laniidae, Monarchidae	Table 29, Fig. 30
Families Alaudidae, Cisticolidae, Melanocharitidae, Paridae	Table 30, Fig. 31
Families Acrocephalidae, Hirundinidae	Table 31, Fig. 32
Families Leiothrichidae, Phylloscopidae, Pycnonotidae, Sylviidae and Zosteropidae	Table 32, Fig. 33
Families Mimidae, Muscicapidae, Sittidae, Sturnidae and Turdidae	Table 33, Fig. 34
Families Dicaeidae, Estrildidae, Fringillidae, Motacillidae, Nectariniidae, Passeridae, Ploceidae	Table 34, Fig. 35
Families Emberizidae, Icteridae, Parulidae, Passerellidae and Thraupidae	Table 35, Fig. 36

^a^Only orders and families of birds were shown, in whose *Haemoproteus* species were identified

## Discussion

The recent discoveries of severe damage caused by tissue stages (meronts and megalomeronts) of *Haemoproteus* parasites in various organs, including the brain, heart, lungs, kidneys and skeletal muscles of naturally infected birds call for research aimed at better understanding the diversity and patterns of development of these pathogens [2, 24, 25, 27, 28, 170, 186]. This work requires parasite species identification and molecular data analysis. The combination of microscopic and PCR-based tools complements each other and increases the significance of research on haemosporidian parasite diversity, so is preferable, particularly in wildlife studies [36, 38, 40]. However, the progress in developing morphological identifications of *Haemoproteus* species is slow and noticeably falls behind the accumulation of DNA sequence information. This is unfortunate because the identification of the parasite species helps in accessing the basic data on the life cycles and patterns of pathogens’ development in various hosts and would be helpful for better understanding of haemoproteosis. The easy-to-use keys should stimulate taxonomic research and contribute to the discovery of new pathogen species by providing direct indications how to distinguish the parasites, which are morphologically different from described ones. In other words, the samples under identification, which are absent in the keys, likely represent new pathogens and would be worth targeted taxonomic investigation.

The following possible obstacles should be considered during the identification of *Haemoproteus* parasites using samples collected in wildlife. First, the intensity of parasitaemia is often low in naturally infected birds, and all necessary blood stages (young, growing or fully grown gametocytes) might be absent in a single sample. Experienced taxonomists can often perform the species identification by visualization of several *Haemoproteus* gametocytes, which are typical for the species, however this is hardly achievable for novices. As a result, low parasitaemia might limit the use of the keys. The negative effect of this factor can be minimized by sampling a sufficient number of host individuals belonging to the same species at the same study site. Extensive sampling of the same host species is common in population studies. The sample size needed for parasite taxonomical work depends on the prevalence of infection in a certain bird population [187]. The sampling of 10–30 host individuals often is sufficient and usually provides an opportunity to access various intensities of parasitaemia of the same parasite and then to visualize the full range of gametocytes, which are necessary for parasite species identification. This is preferable for the development of comprehensive descriptions of new pathogens.

Second, gametocytes of *Haemoproteus* inhabit erythrocytes, which are fragile cells and might be deformed during the preparation of blood films, resulting in presence of unusual parasite forms, which are artefacts from the taxonomic point of view and should be ignored during species identification and parasite descriptions. Morphological characters of typical (the most common) non-deformed gametocytes and their host cells should be selected for identification. Only such cells were used and shown in all corresponding illustrations in the keys (Figs. 3, 4, 5, 6, 7, 8, 9, 10, 11, 12, 13, 14, 15, 16, 17, 18, 19, 20, 21, 22, 23, 24, 25, 26, 27, 28, 29, 30, 31, 32, 33, 34, 35, 36).

**Fig. 3:**
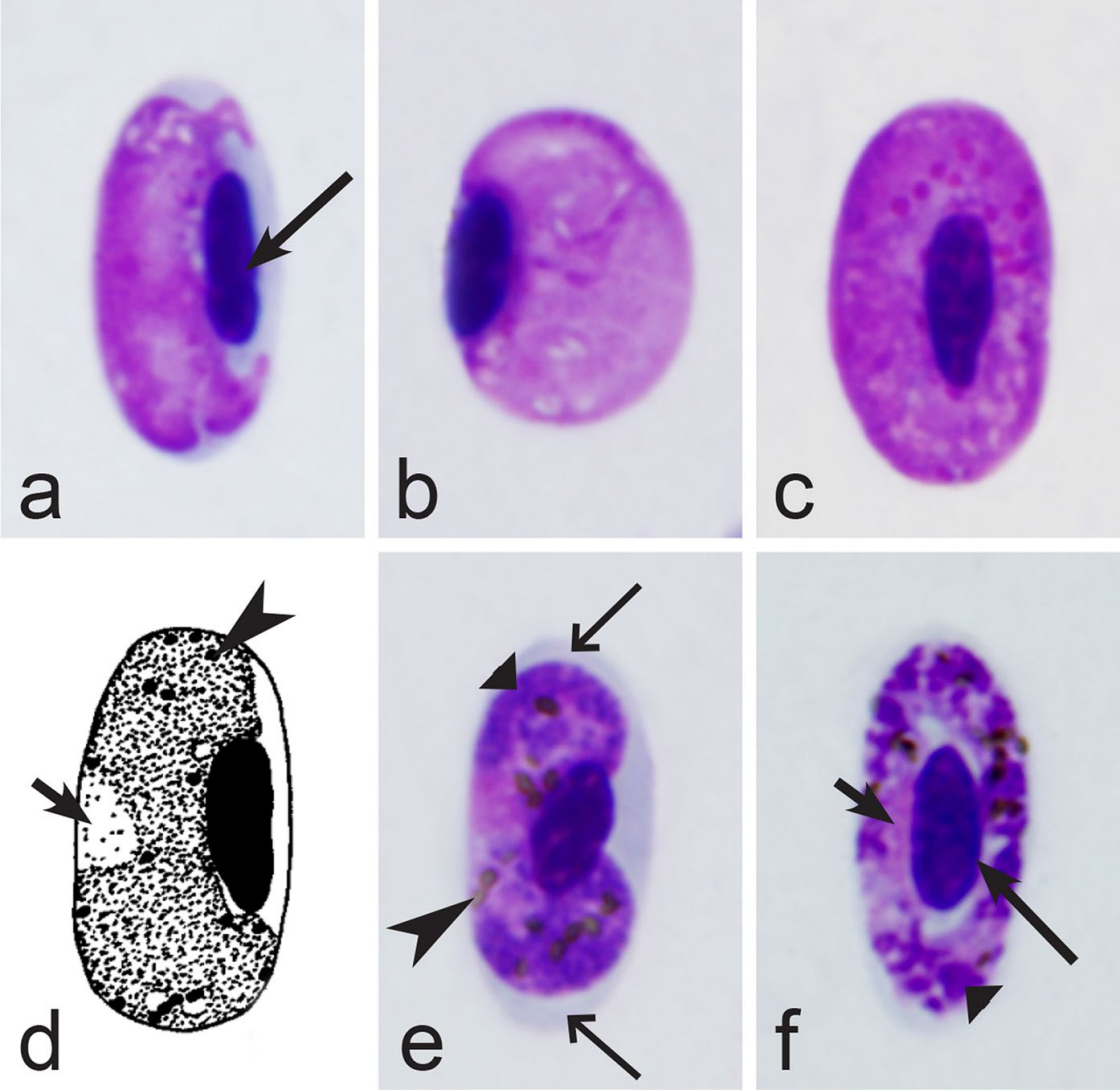
Morphological features of gametocytes, which are used for identification of *Haemoproteus* species parasitizing Accipitriformes birds. Microgametocytes (**a**–**c**) and macrogametocytes (**d**–**f**) of *Haemoproteus janovyi* (**a**–**c**), *H. elani* (**d**), *H. buteonis* (**e**) and *H. nisi* (**f**). Note the markedly variable form of *H. janovyi* gametocytes (**a**–**c**), the broadly halteridial form of *H. elani* gametocyte (**d**), the presence of unfilled spaces on the poles of infected erythrocytes during *H. buteonis* infection (**e**), and the circumnuclear form of *H. nisi* gametocyte, which is overfilled with volutin granules (**f**). Images **a**–**c** are from the type material, which is fading, resulting in pale staining and poorly recognizable pigment granules and nuclei, however the overall form of the gametocytes is readily visible. Long simple arrows—host cell nuclei. Short simple arrows—parasite nuclei. Simple arrowheads—pigment granules. Simple wide long arrows—unfilled space on poles of infected erythrocytes. Triangle wide arrowheads—volutin granules. Other explanations are given in the text

**Fig. 4:**
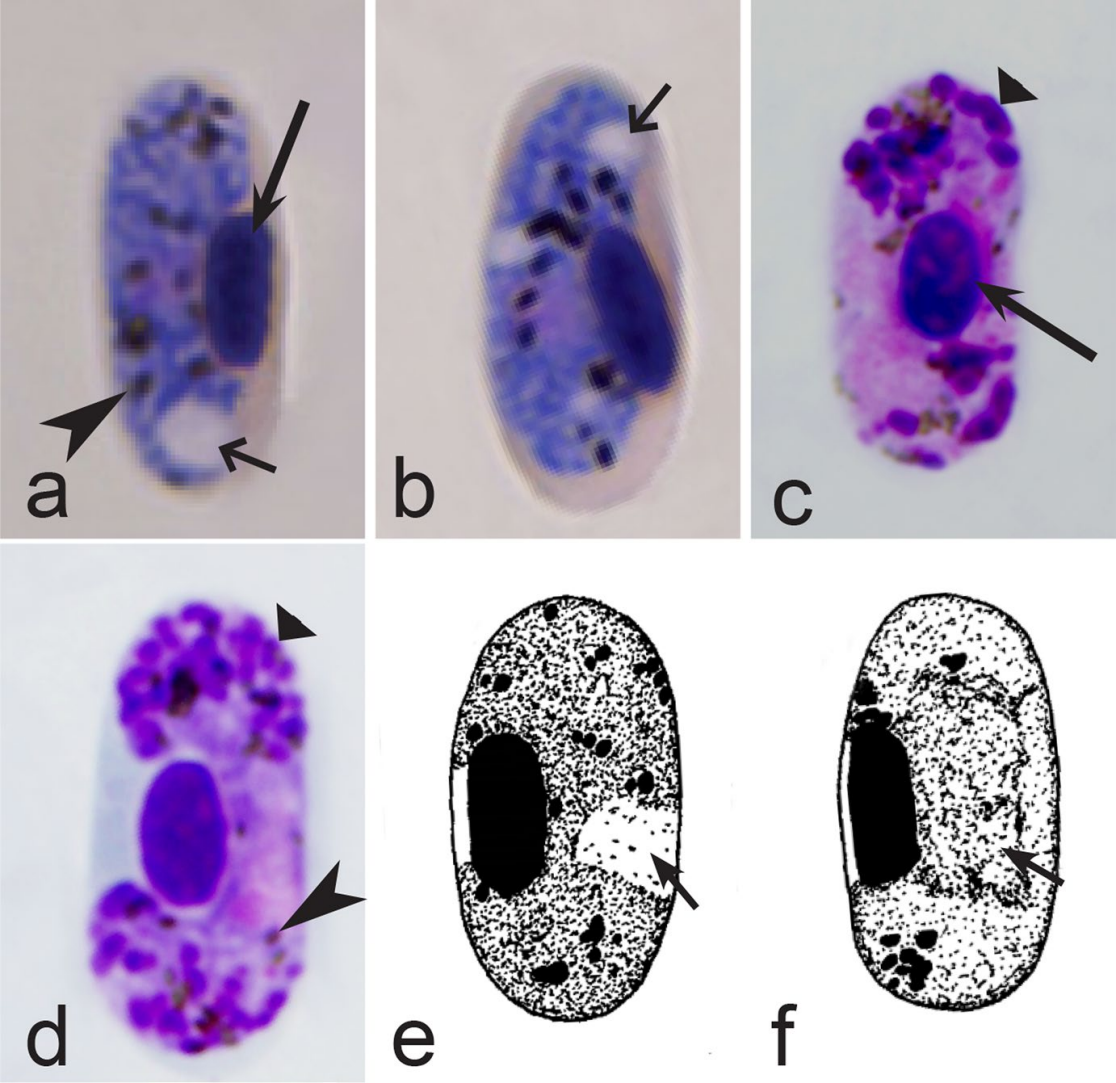
Morphological features of gametocytes, which are used for identification of *Haemoproteus* species parasitizing Anseriformes birds. Macrogametocytes (**a**, **b**, **e**, **d**) and microgametocytes (**c**, **f**) of *Haemoproteus macrovacuolatus* (**a**, **b**), *H. greineri* (**c**, **d**) and *H. nettionis* (**e**, **f**). Note the presence of large vacuoles in the cytoplasm of *H. macrovacuolatus* macrogametocytes (**a**, **b**). Fully grown gametocytes of *H. greineri* are predominantly close to circumnuclear (**d**) and circumnuclear (**c**) in form, but fully grown gametocytes of *H. nettionis* are predominantly broadly halteridial (**e**, **f**). Prominent volutin granules (**c**, **d**) are present in gametocytes of *H. greineri* and *H. nettionis* (volutin was not shown in white-and black pictures **e**, **f**); these species are indistinguishable based on this character. Long simple arrows—host cell nuclei. Short simple arrows—parasite nuclei. Simple arrowheads—pigment granules. Simple wide short arrows—vacuoles. Triangle wide arrowheads—volutin granules. Other explanations are given in the text

**Fig. 5:**
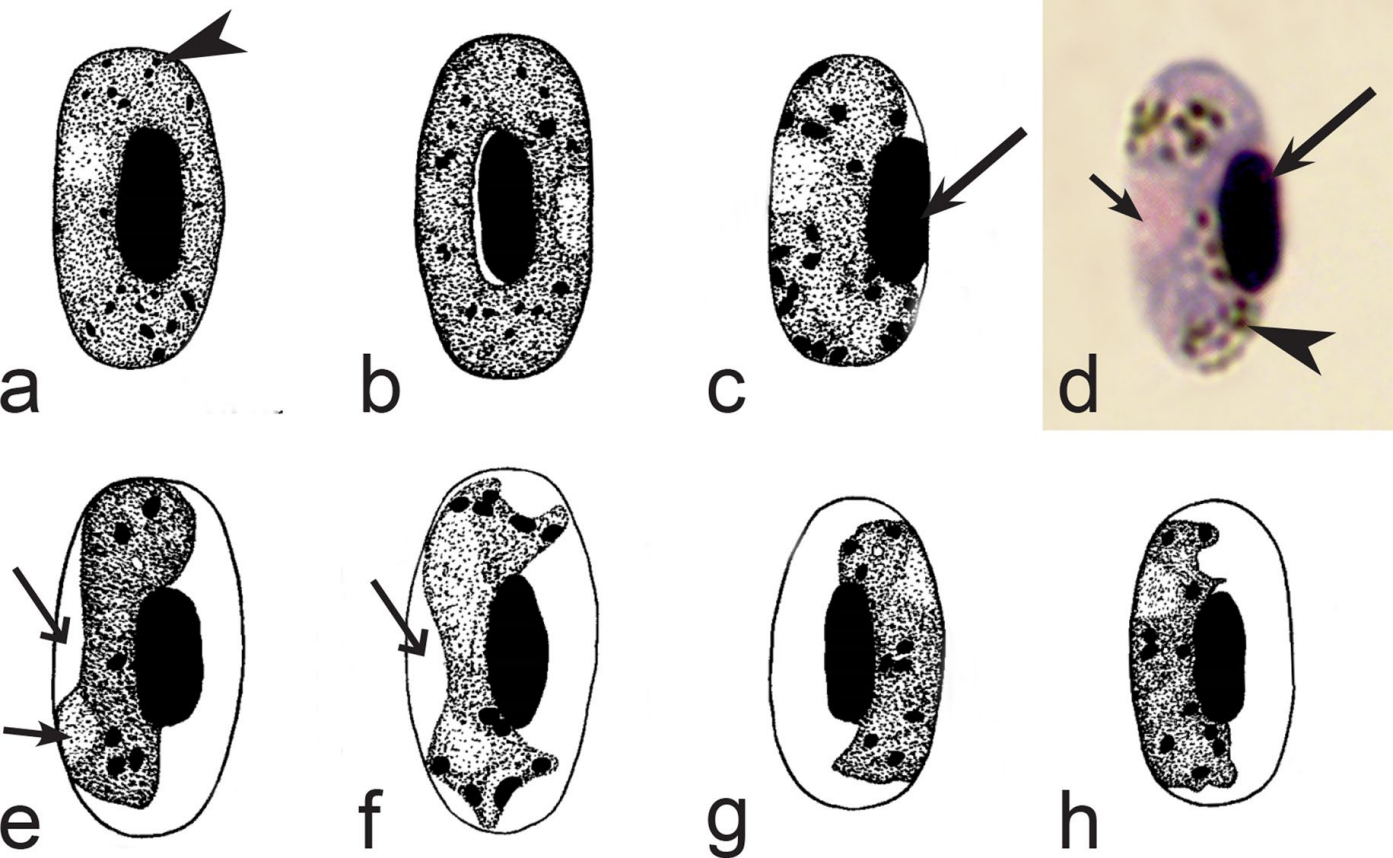
Morphological features of gametocytes, which are used for identification of *Haemoproteus* species parasitizing Apodiformes birds. Macrogametocytes (**a**–**e**, **g**, **h**) and microgametocyte (**f**) *Haemoproteus archilochus* (**a**, **b**), *H. witti* (**c**, **d**), *H. apodus *(**e**, **f**) and *H. trochili* (**g**, **h**). Note the circumnuclear (**a**, **b**) and broadly halteridial (**c**, **d**) forms of fully grown gametocytes in *H. archilochus* (**a**, **b**) and *H. witti* (**c**, **d**)*,* respectively. Gametocytes of *H. apodus *(**e**, **f**) and *H. trochili* (**g**, **h**) are microhalteridial in form. *Haemoproteus apodus* is readily distinguishable due to presence of numerous dumbbell-like growing gametocytes (**e**, **f**). Long simple arrows—host cell nuclei. Short simple arrows—parasite nuclei. Simple arrowheads—pigment granules. Simple wide long arrows—unfilled space between growing gametocytes and envelope of infected erythrocyte. Other explanations are given in the text

**Fig. 6:**
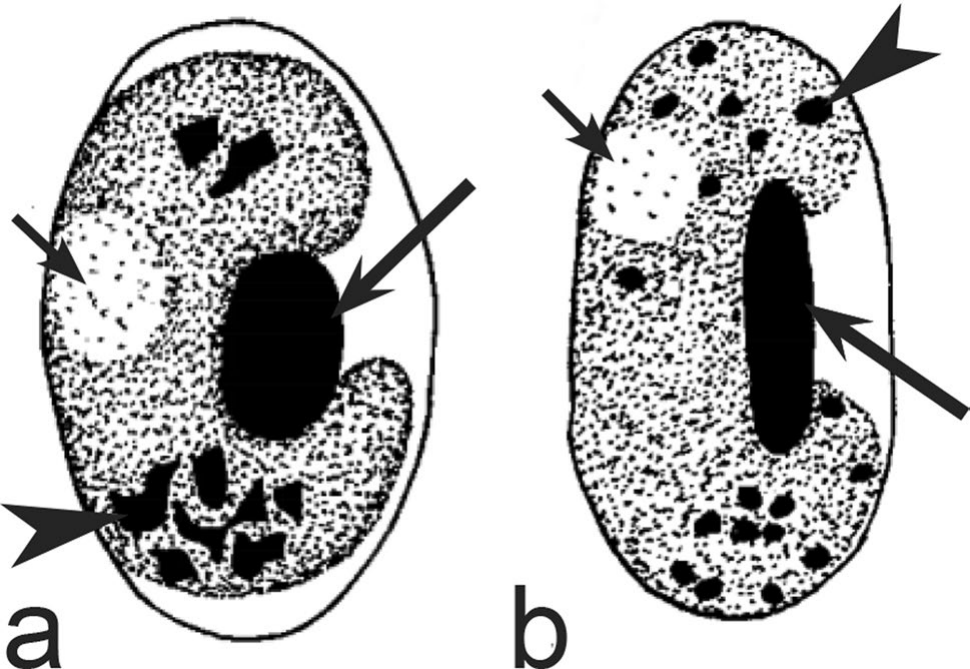
Morphological features of gametocytes, which are used for identification of *Haemoproteus* species parasitizing Bucerotiformes birds. Macrogametocytes (**a**, **b**) of *Haemoproteus upupae* (**a**) and *H. bucerotis* (**b**). Note the different position of nuclei in macrogametocytes—close to central (**a**) and subcentral (**b**)—of these species, and the different morphology and mode of distribution of pigment granules in the cytoplasm (compare **a** with **b**). Long simple arrows—host cell nuclei. Short simple arrows—parasite nuclei. Simple arrowheads—pigment granules. Other explanations are given in the text

**Fig. 7:**
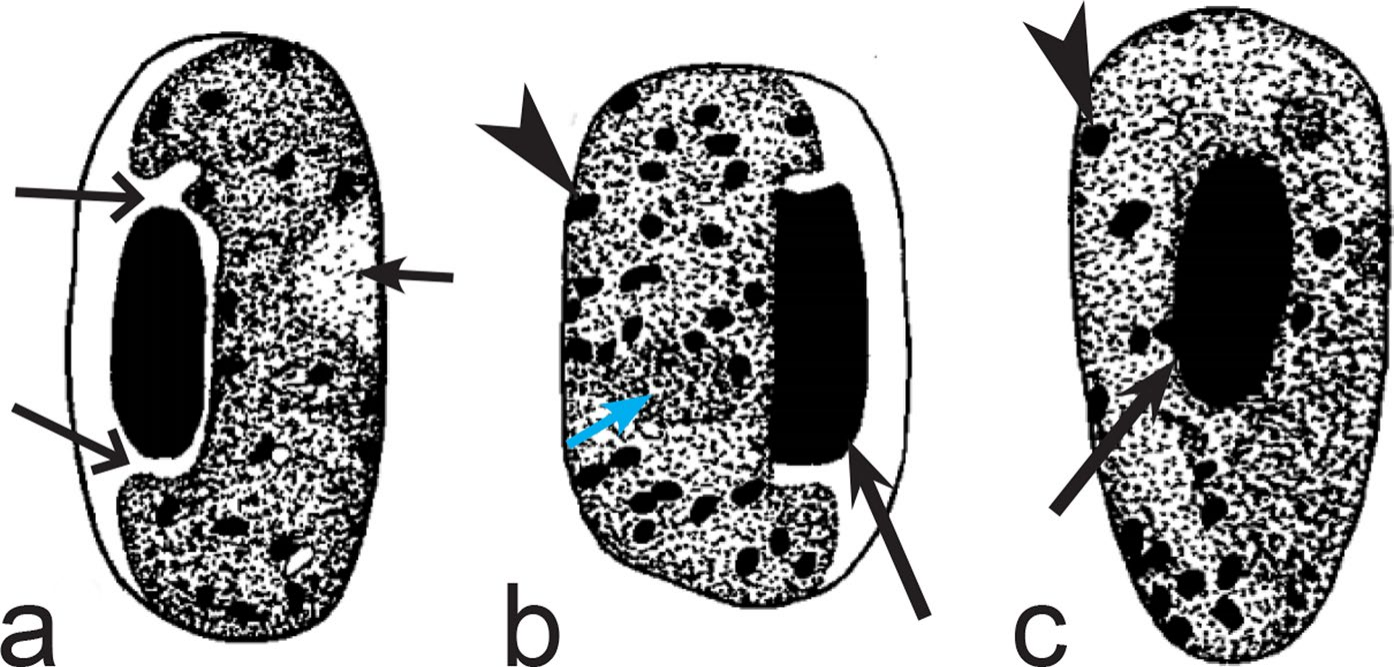
Morphological features of gametocytes, which are used for identification of *Haemoproteus* species parasitizing Caprimulgiformes birds. Macrogametocytes (**a**, **b**) and microgametocyte (**c**) of *Haemoproteus caprimulgi* (**a**–**c**). Note the markedly pleomorphic form of fully grown gametocytes (**b**, **c**). Advanced growing gametocytes often do not adhere to the nuclei of infected erythrocytes (**a**). Long simple arrows—host cell nuclei. Short simple arrows—parasite nuclei. Simple arrowheads—pigment granules. Simple wide long arrows—unfilled space between growing gametocyte and the nucleus of infected erythrocyte. Other explanations are given in the text

**Fig. 8:**
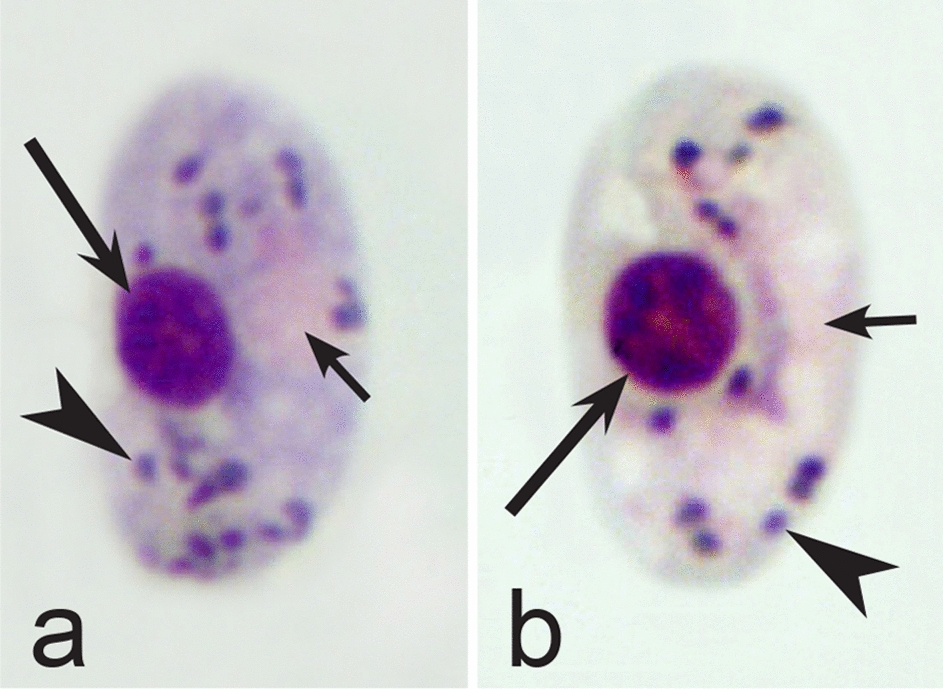
Morphological features of gametocytes, which are used for identification of *Haemoproteus* species parasitizing Cariamiformes birds. Macrogametocyte (**a**) and microgametocyte (**b**) of *H. pulcher* (**a**, **b**). Note that advanced growing gametocytes often do not adhere to the nuclei of infected erythrocytes (**b**), and the infected erythrocytes nuclei assume roundish form (**a**, **b**). Long simple arrows—host cell nuclei. Short simple arrows—parasite nuclei. Simple arrowhead—pigment granules. Other explanations are given in the text

**Fig. 9:**
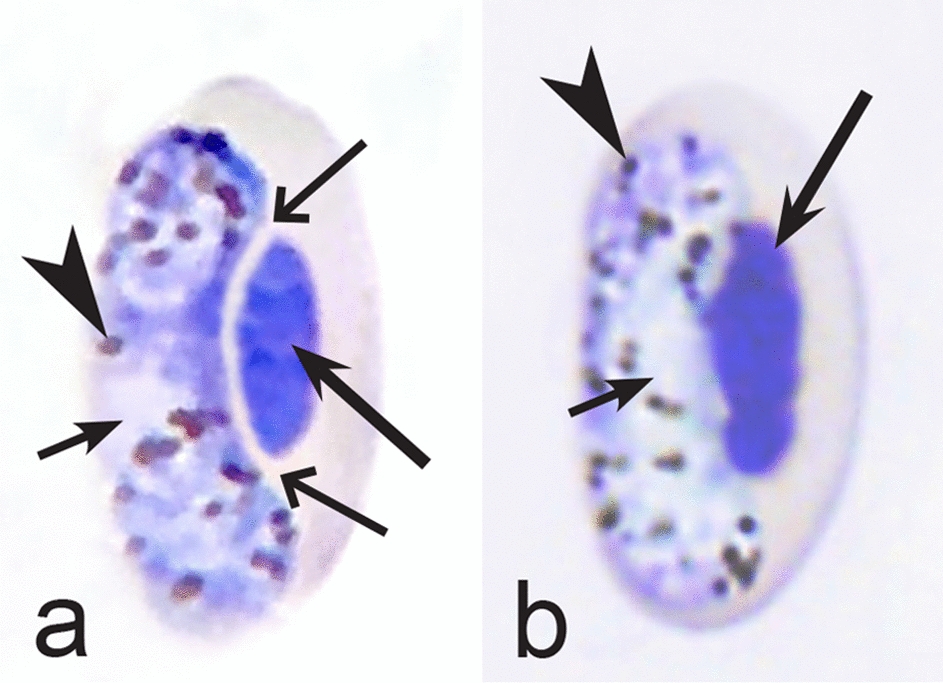
Morphological features of gametocytes, which are used for identification of *Haemoproteus* species parasitizing Cathartiformes birds. Macrogametocyte (**a**) and microgametocyte (**b**) of *Haemoproteus catharti* (**a**, **b**). Note that advanced growing gametocytes often do not adhere to erythrocyte nuclei (**a**). Pigment granules are of medium size and numerous (**a**, **b**). Long simple arrows—host cell nuclei. Short simple arrows—parasite nuclei. Simple arrowheads—pigment granules. Simple wide long arrows—unfilled space between growing gametocyte and nucleus of infected erythrocyte. Other explanations are given in the text

**Fig. 10:**
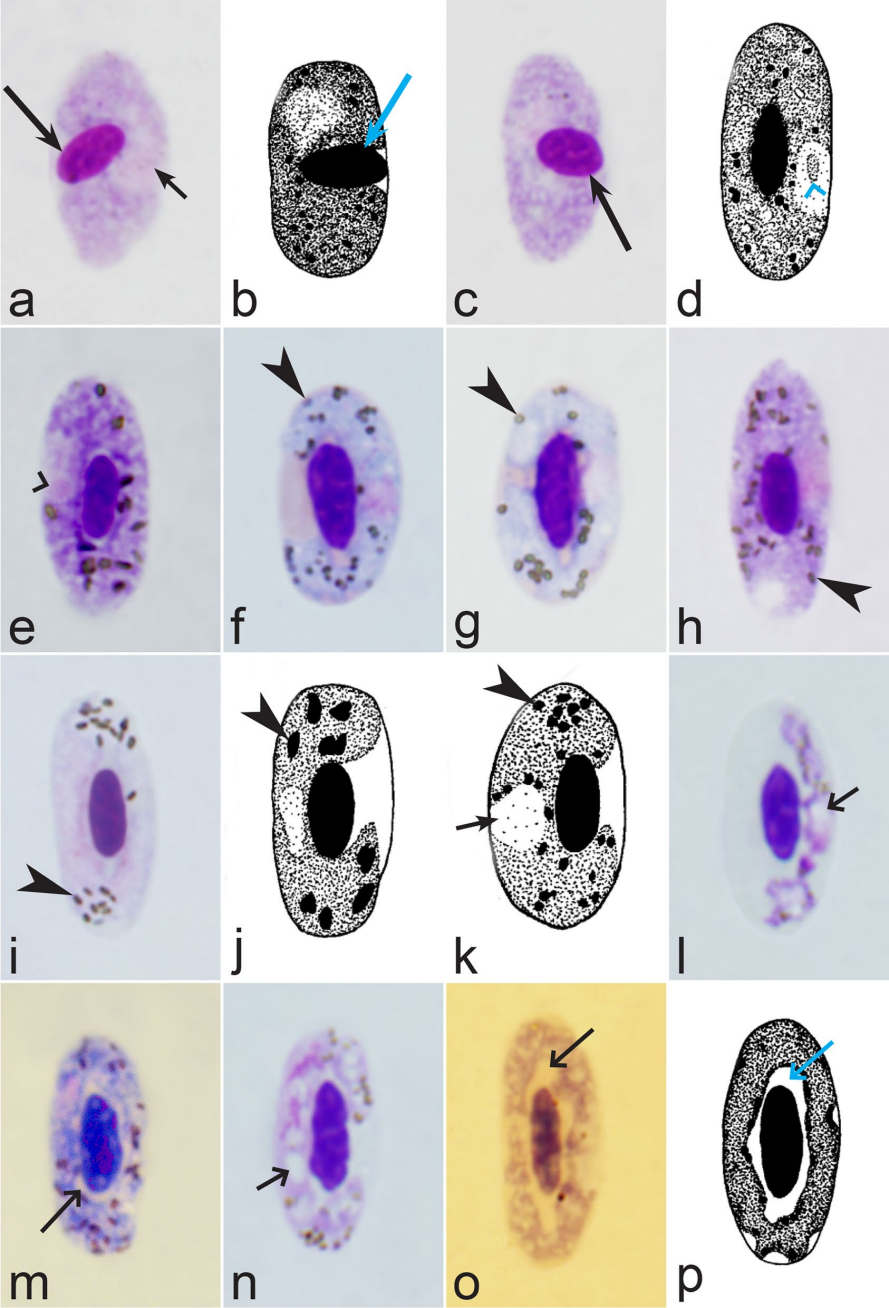
Morphological features of gametocytes, which are used for identification of *Haemoproteus* species parasitizing Charadriiformes birds. Macrogametocytes (**a**–**f**, **h**, **j**–**m**, **o**, **p**) and microgametocytes (**g**, **i**, **n**) of *Haemoproteus rotator* (**a**–**c**), *H. scolopaci* (**d**, **e**), *H. jenniae* (**f**, **g**), *H. larae* (**h**, **i**), *H. abdusalomovi* (**j**), *H. burhini* (**k**), *H. skuae* (**l**–**n**), *H. contortus* (**o**, **p**). Note that fully grown gametocytes of *H. rotator* markedly rotate the nuclei of infected erythrocytes (**a**–**c**). Nucleolus is readily visible in nuclei of *H. scolopaci* macrogametocytes (**d**, **e**). Fully grown gametocytes of *H. jenniae* contain predominantly roundish or slightly oval and of approximately uniform size and form pigment granules (**f**, **g**), which are different from the predominantly elongate rod-like pigment granules in gametocytes of *H. larae* (**h**, **i**). More or less evident unfilled spaces are present between circumnuclear macrogametocytes and nuclei of infected erythrocytes in *H. skuae* (**m**) and *H. contortus* (**p**). Images **a**, **c**, **l**–**n**, **o** are from the type material, which is fading, resulting in pale staining and poorly recognizable pigment granules and nuclei, however the overall form of the gametocytes is readily visible. Long simple arrows—host cell nuclei. Short simple arrows—parasite nuclei. Simple arrowheads—pigment granules. Simple wide short arrows—vacuoles. Simple wide long arrows—unfilled spaces between gametocytes and nuclei of infected erythrocytes. Other explanations are given in the text

**Fig. 11:**
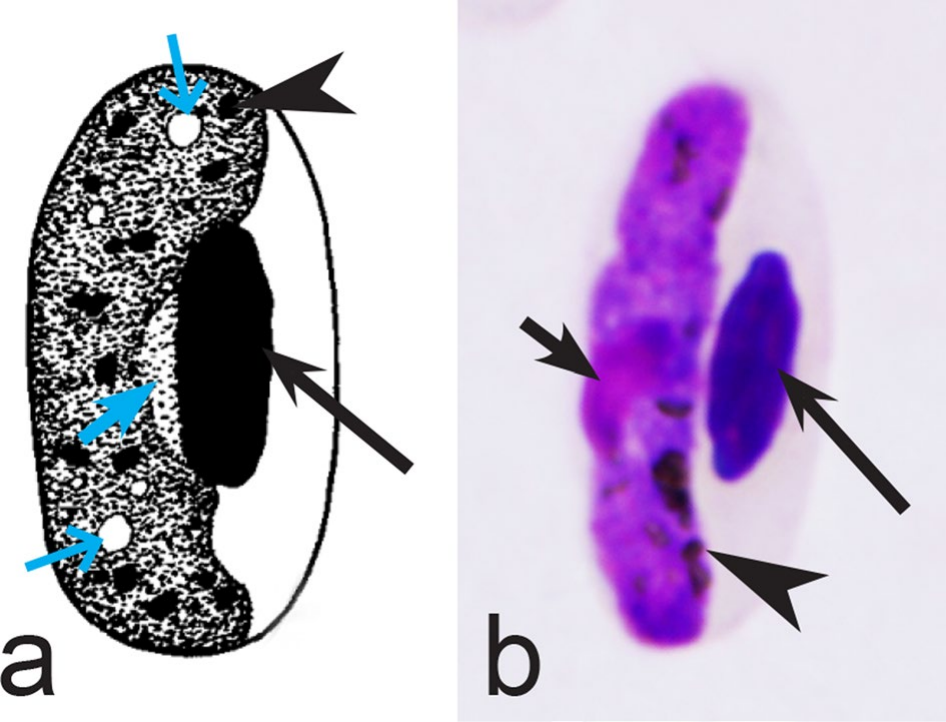
Morphological features of gametocytes, which are used for identification of *Haemoproteus* species parasitizing Ciconiiformes birds. Macrogametocytes (**a**, **b**) of *Haemoproteus crumenium* (**a**) and *H. ciconiae* (**b**). Note that nucleus of *H. crumenium* macrogametocyte locate close the erythrocyte nucleus (**a**), but this is usually not a case in *H. ciconiae* (**b**). Long simple arrows—host cell nuclei. Short simple arrows—parasite nuclei. Simple arrowheads—pigment granules. Simple wide short arrows—vacuoles. Other explanations are given in the text

**Fig. 12:**
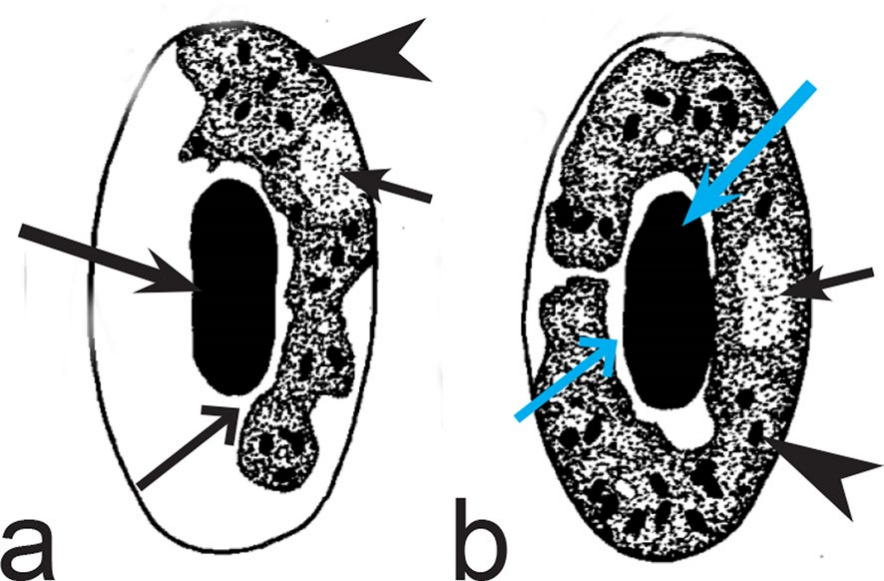
Morphological features of gametocytes, which are used for identification of *Haemoproteus* species parasitizing Coliiformes birds. Macrogametocytes of *Haemoproteus undulatus* (**a**, **b**). Note that growing gametocytes are usually appressed to the envelope of infected erythrocytes but do not touch the nuclei of erythrocytes along their entire margin (**a**). Form of advanced gametocytes is close to circumnuclear (**b**) or circumnuclear. Long simple arrows—host cell nuclei. Short simple arrows—parasite nuclei. Simple arrowheads—pigment granules. Simple wide long arrows—unfilled spaces between gametocytes and nuclei of infected erythrocytes. Other explanations are given in the text

**Fig. 13:**
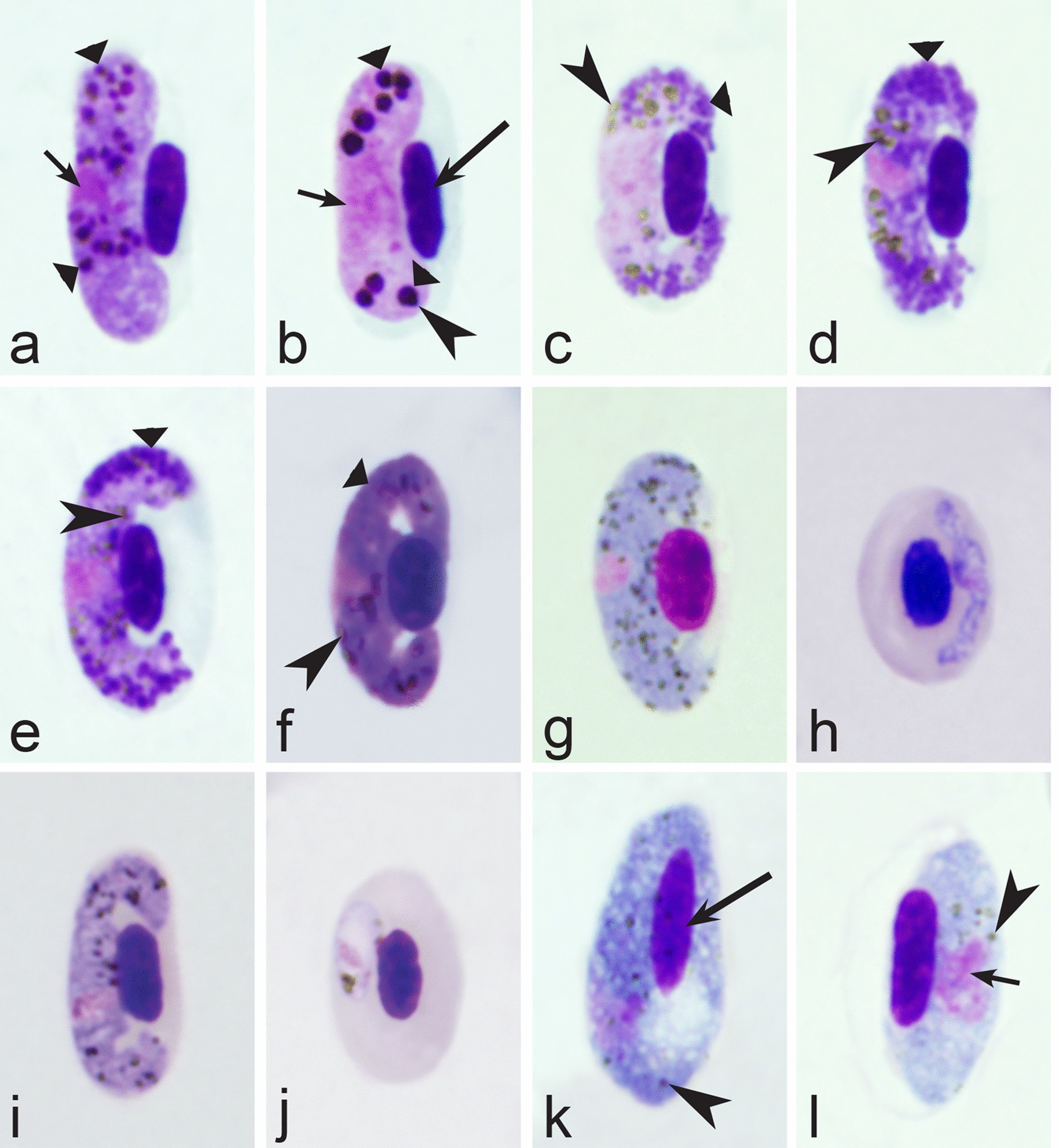
Morphological features of fully grown (**a**–**g**, **i**, **k**, **l**) and young (**h**, **j**) gametocytes, which are used for identification of *Haemoproteus* species parasitizing Columbiformes birds. Macrogametocytes (**a**, **d**–**k**) and microgametocytes (**b**, **c**, **l**) of *Haemoproteus columbae* (**a, b**), *H. turtur* (**c**, **d**), *H. palumbis* (**e**), *H. multivolutinus* (**f**), *H. multipigmentatus *(**g, h**), *H. paramultipigmentatus *(**i**, **j**) and *H. sacharovi *(**k**, **l**). Note that big roundish volutin granules present in gametocytes of *H. columbae* (**a**, **b**), and the small pigment granules locate inside these volutin granules but usually are not present free in the cytoplasm (**b**). Numerous discrete roundish volutin granules present in gametocytes of *H. turtur* (**c**, **d**) and *H. palumbis* (**e**), and pigment granules are readily visible in the cytoplasm of both these species (**d**, **e**). Volutin overfills the cytoplasm in gametocytes in *H. multivolutinus* (**f**). Young gametocytes of *H. multipigmentatus *(**h**) and *H. paramultipigmentatus *(**j**) are markedly different in form. Gametocytes of *H. sacharovi *(**k**, **l**) are outwardly similar to gametocytes of *Leucocytozoon* parasites. Long simple arrows—host cell nuclei. Short simple arrows—parasite nuclei. Simple arrowheads—pigment granules. Triangle wide arrowheads—volutin. Other explanations are given in the text

**Fig. 14:**
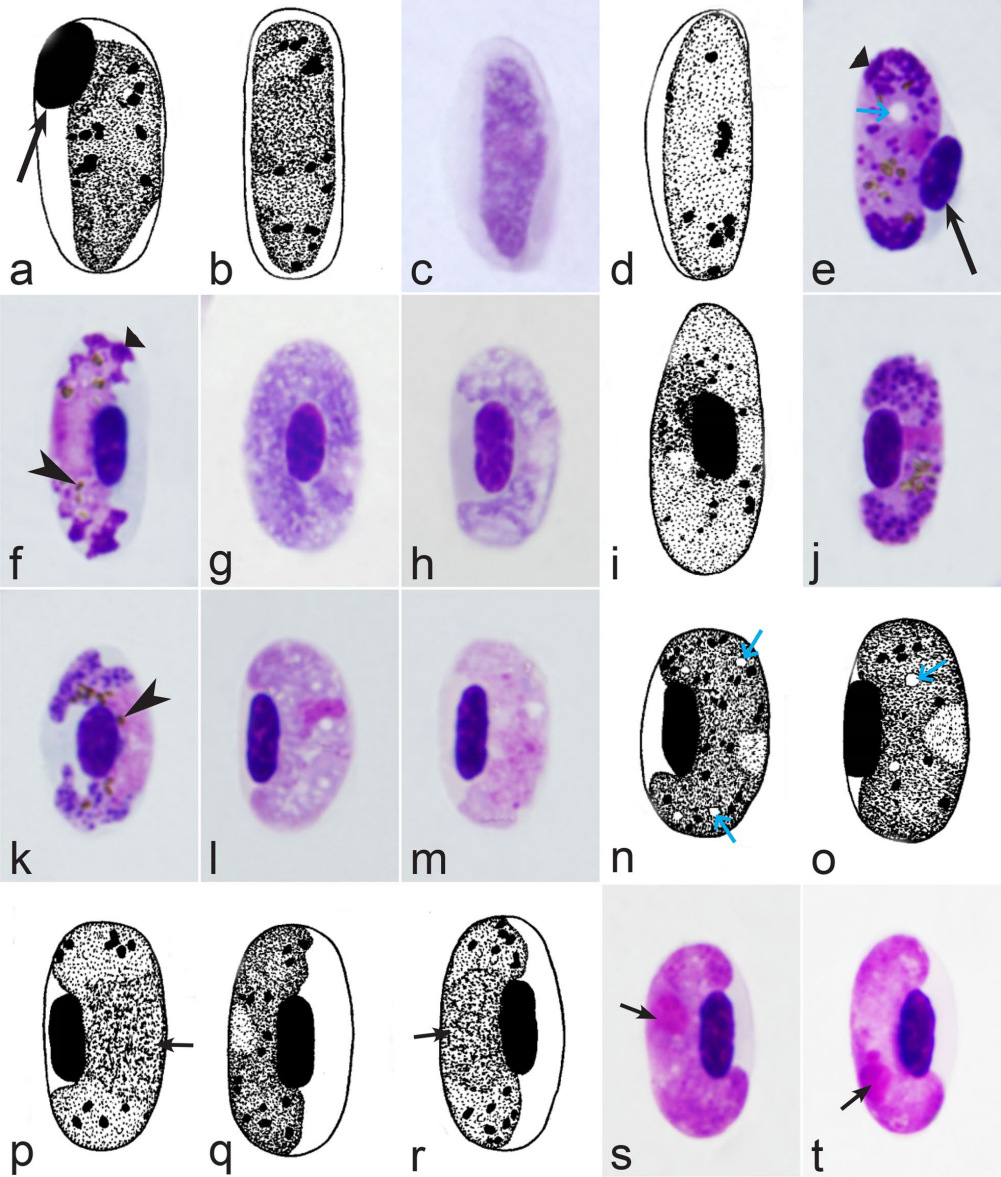
Morphological features of gametocytes, which are used for identification of *Haemoproteus* species parasitizing Coraciiformes birds. Macrogametocytes (**a**, **c**, **e**, **g**–**j**, **l**, **n**, **o**, **q**, **s**) and microgametocytes (**b**, **d**, **f**, **k**, **m**, **p**, **r**, **t**) of *Haemoproteus lairdi* (**a**, **b**), *H. enucleator* (**c**, **d**), *H. gavrilovi* (**e**, **f**), *H. fuscae* (**g**–**i**), *H. coraciae* (**j**, **k**), *H. eurystomae* (**l**–**n**), *H. manwelli* (**o**, **p**)*, H. meropis* (**q**, **r**) and *H. halcyonis* (**s**, **t**). Note the presence of rhabdosomal gametocytes in *H. lairdi* (**b**) and *H. enucleator* (**c**, **d**). One big circular vacuole is often present in the cytoplasm of *H. gavrilovi* macrogametocyte (**e**). Nucleus of *H. halcyonis* microgametocyte is condensed (**t**) and is similar in size to macrogametocyte nucleus (**s**), which is a rare feature in avian haemoproteids. Images **c**, **g**, **h**, **l**, **m**, **s**, **t** are from the type material, which is fading, resulting in pale staining and poorly recognizable pigment granules and nuclei, however the overall form of gametocytes is readily visible. Long simple arrows—host cell nuclei. Short simple arrows—parasite nuclei. Simple arrowheads—pigment granules. Simple wide short arrows—vacuoles. Triangle wide arrowheads—volutin granules. Other explanations are given in the text

**Fig. 15:**
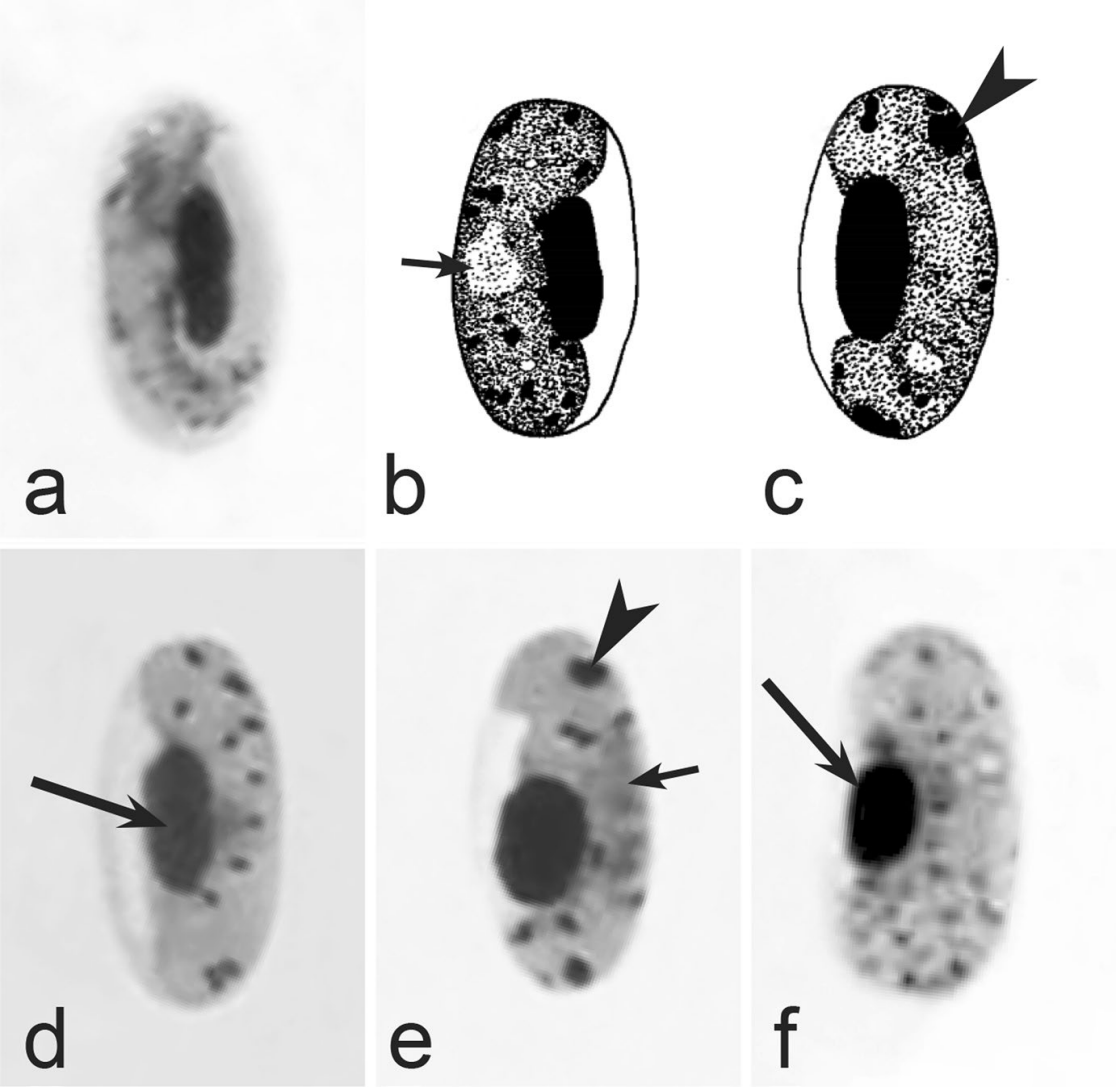
Morphological features of gametocytes, which are used for identification of *Haemoproteus* species parasitizing Cuculiformes birds. Macrogametocytes (**a**–**f**) of *Haemoproteus cuculis* (**a**), *H. centropi* (**b**–**e**) and *H. clamatori* (**f**). Note that pigment granules in *H. centropi* gametocytes tend to aggregate into compact large masses (**c**) or loosely aggregated clumps (**e**). Long simple arrows—host cell nuclei. Short simple arrows—parasite nuclei. Simple arrowheads—pigment granules. Other explanations are given in the text

**Fig. 16:**
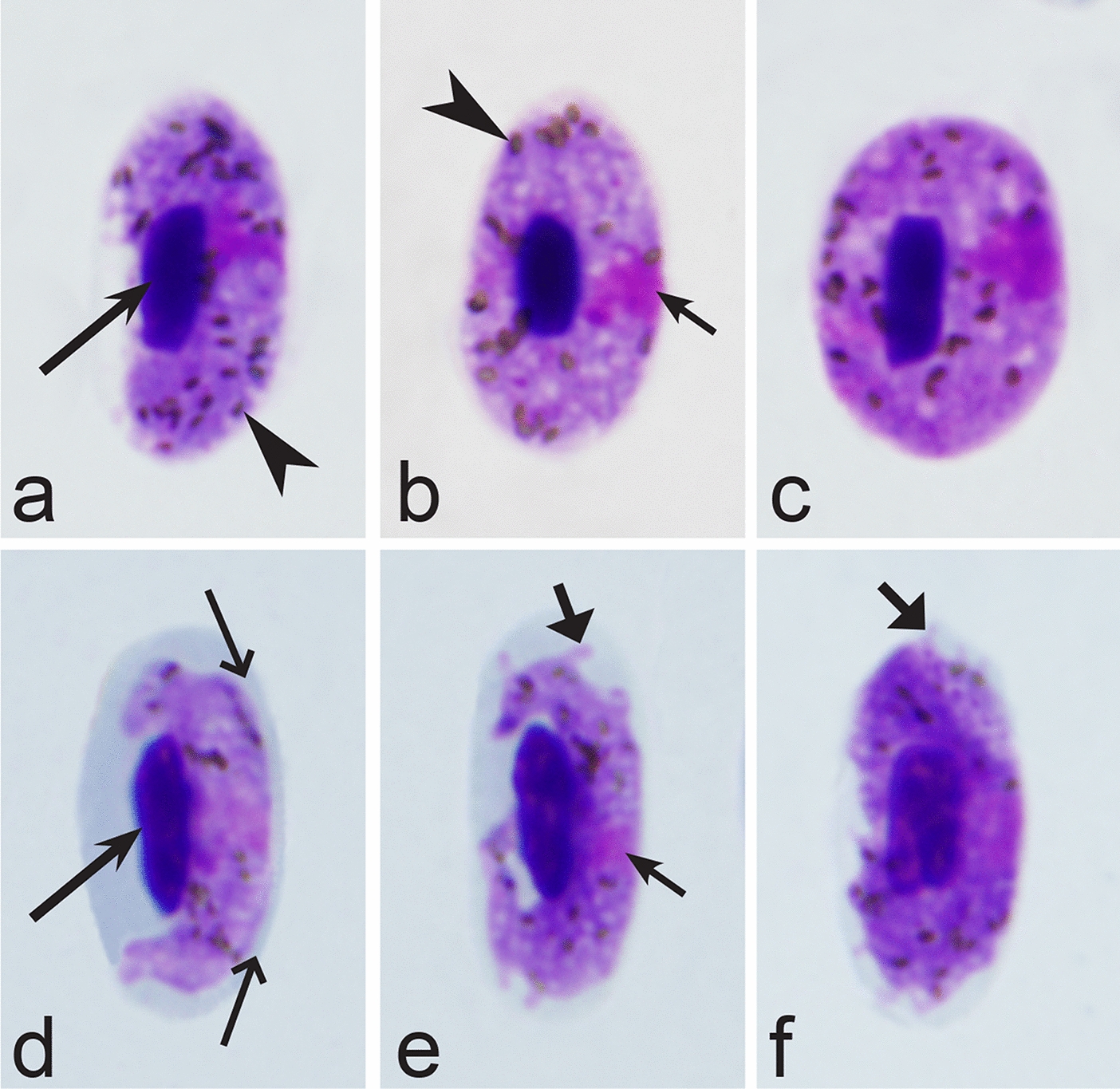
Morphological features of gametocytes, which are used for identification of *Haemoproteus* species parasitizing Falconiformes birds. Macrogametocytes (**a**–**f**) of *Haemoproteus tinnunculi* (**a**–**c**) and *H. brachiatus* (**d**–**f**). Note that growing gametocytes of *H. brachiatus* are highly irregular or amoeboid in outline (**d**, **e**). Advanced growing gametocytes of *H. brachiatus* often do not adhere to envelope of erythrocytes (**d**), which is not a case in *H. tinnunculi* (**a**). Long simple arrows—host cell nuclei. Short simple arrows—parasite nuclei. Simple arrowheads—pigment granules. Simple wide long arrows—a space between gametocyte and envelope of infected erythrocyte. Triangle wide arrows—ameboid outgrowths. Other explanations are given in the text

**Fig. 17:**
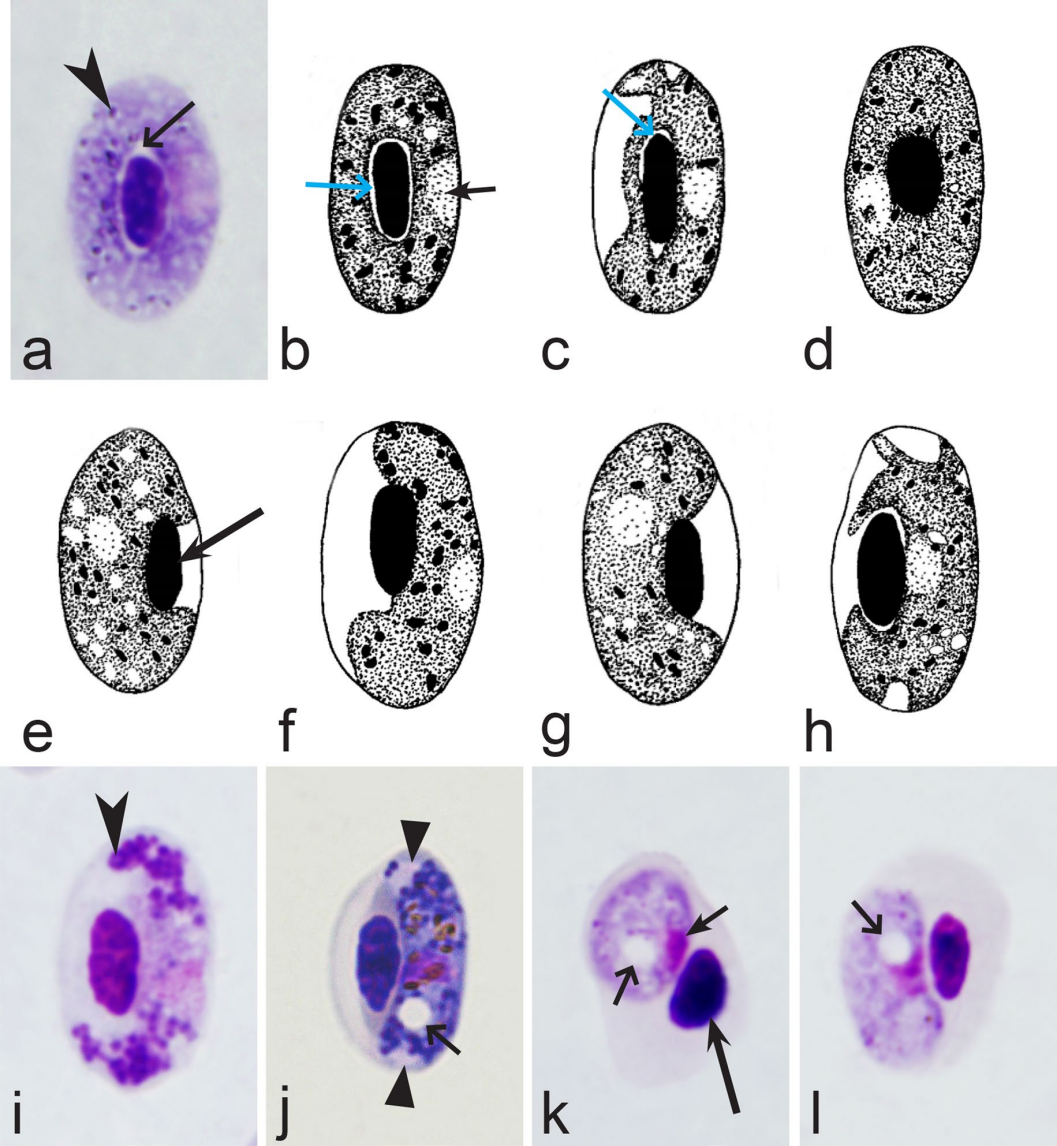
Morphological features of gametocytes, which are used for identification of *Haemoproteus* species parasitizing Galliformes birds. Macrogametocytes (**a**–**h, j**–**l**) and microgametocytes (**i**) of *Haemoproteus lophortyx* (**a**, **b**), *H. stableri* (**c**), *H. mansoni* (**d**), *H. pratasi* (**e**), *H. ammoperdix* (**f**), *H. rileyi* (**g**), *H. cracidarum* (**h**, **i**), *H. paraortalidum* (**j**) and *H. ortalidum* (**k**, **l**). Note that macrogametocytes of *H. paraortalidum* and *H. ortalidum* contain one large (bigger than 1 µm in diameter) circular vacuole (**j**–**l**). An unfilled space (a ‘cleft’) is present between the fully grown gametocytes and the nuclei of infected erythrocytes during development of *H. lophortyx* (**a**, **b**) and *H. stableri* (**c**). Vacuole-like unstained spaces (**j**) are present on both ends of *H. paraortalidum* macrogametocyte. Images **a**, **k**, **l** are from the type material, which is fading, resulting in pale staining and the poorly recognizable pigment granules and nuclei, however the overall form of the gametocytes is readily visible. Long simple arrows—host cell nuclei. Short simple arrows—parasite nuclei. Simple arrowheads—pigment granules. Simple wide long arrows—unfilled spaces between gametocytes and nuclei of infected erythrocytes. Simple wide short arrows—vacuoles. Simple wide arrowheads—unstained spaces on the ends of macrogametocyte. Other explanations are given in the text

**Fig. 18:**
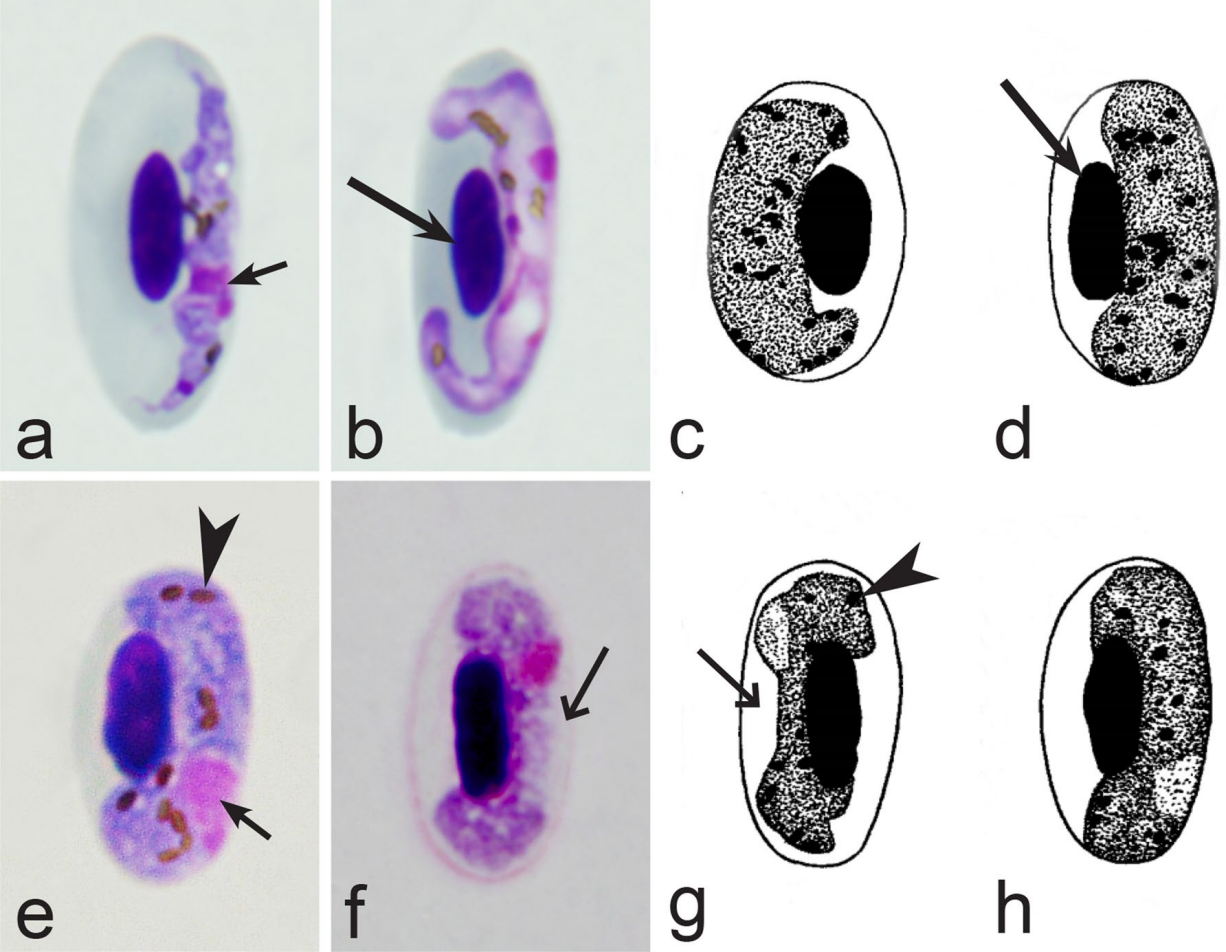
Morphological features of gametocytes, which are used for identification of *Haemoproteus* species parasitizing Gruiformes birds. Macrogametocytes (**a**, **c**–**h**) and microgametocyte (**b**) of *Haemoproteus balearicae* (**a**, **b**), *H. gallinulae* (**c, d**), *H. antigonis* (**e**), *H. porzanae* (**f**–**h**). Note the slender form of *H. balearicae* gametocytes (**a**, **b**) and the presence of dumbbell-shaped growing gametocytes in *H. porzanae* (**f**, **g**). Long simple arrows—host cell nuclei. Short simple arrows—parasite nuclei. Simple arrowheads—pigment granules. Simple wide long arrows—spaces between gametocytes and envelope of infected erythrocytes. Other explanations are given in the text

**Fig. 19:**
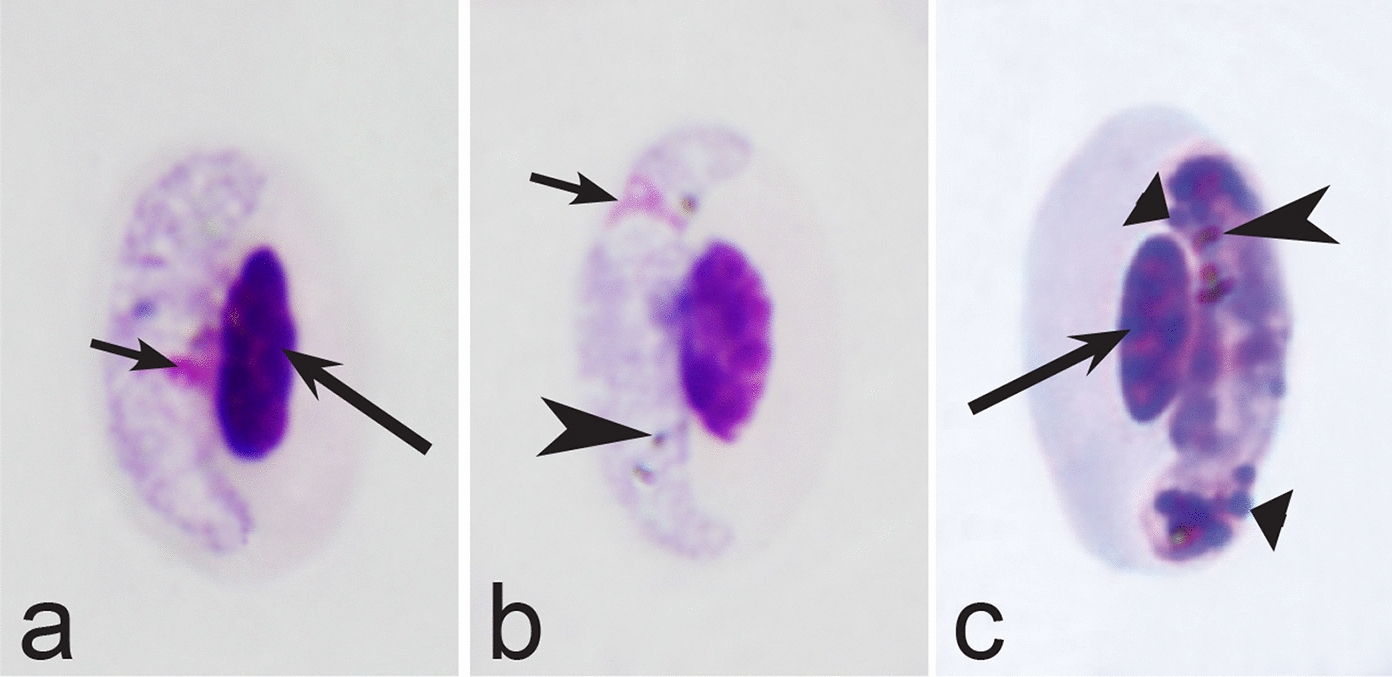
Morphological features of gametocytes, which are used for identification of *Haemoproteus* species parasitizing Musophagiformes birds. Macrogametocytes (**a-c**) of *Haemoproteus montezi* (**a**, **b**) and *H. minchini* (**c**). Note that both ends of advanced *H. montezi* gametocytes usually are more or less narrowed in comparison to the widths of the gametocytes (**b**), but this is not a case in *H. minchini* gametocytes, which both ends usually are approximately similarly rounded (**c**). Images **a**, **b** are from the type material, which is fading, resulting in pale staining and the poorly recognizable pigment granules and nuclei, however the overall form of the gametocytes is readily visible. Long simple arrows—host cell nuclei. Short simple arrows—parasite nucleus. Simple arrowheads—pigment granules. Triangle wide arrowheads—volutin granules. Other explanations are given in the text

**Fig. 20:**
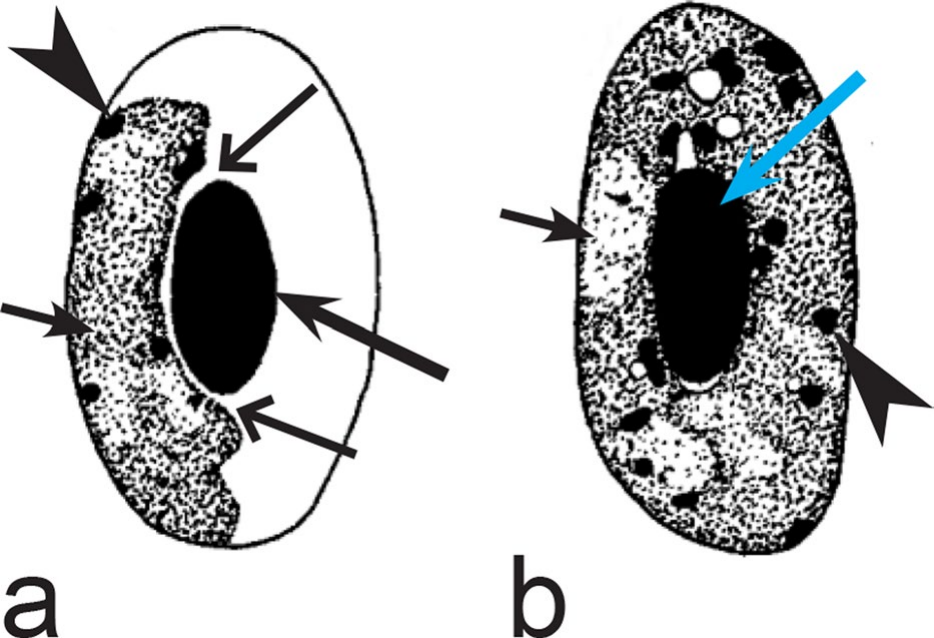
Morphological features of gametocytes, which are used for identification of *Haemoproteus* species parasitizing Otidiformes birds. Macrogametocytes of *Haemoproteus telfordi* (**a**, **b**). Note that the growing gametocytes are closely appressed to the erythrocyte envelope but usually do not touch the erythrocyte nuclei (**a**). Long simple arrow—host cell nucleus. Short simple arrows—parasite nuclei. Simple arrowheads—pigment granules. Simple wide long arrows—a space between gametocyte and nucleus of infected erythrocyte. Other explanations are given in the text

**Fig. 21:**
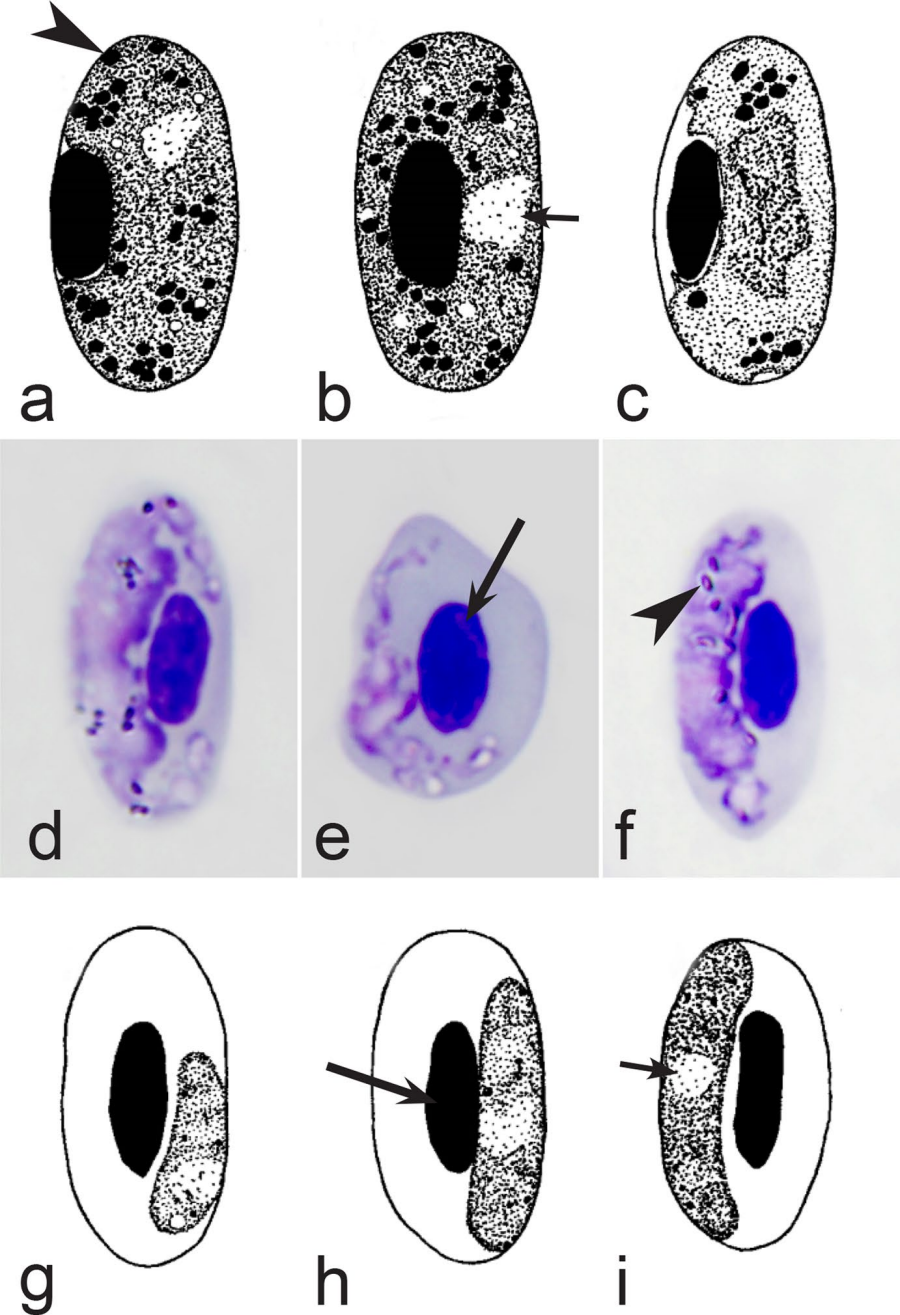
Morphological features of gametocytes, which are used for identification of *Haemoproteus* species parasitizing Pelecaniformes birds. Macrogametocytes (**a**, **b, d**–**i**) and microgametocytes (**c**) *Haemoproteus plataleae* (**a**–**c**), *H. pelouroi* (**d**–**f**) and *H. herodiadis* (**g**–**i**). Note the pleomorphic form of fully grown gametocytes of *H. plataleae* (**a**–**c**). Gametocyte outline is predominantly markedly irregular in *H. pelouroi* (**d**–**f**), but is smooth in *H. herodiadis* (**g**–**i**). Images **d**–**f** are from the type material, which is fading, resulting in pale staining and the poorly recognizable pigment granules and nuclei, however the overall form of the gametocytes is readily visible. Long simple arrows—host cell nuclei. Short simple arrows—parasite nuclei. Simple arrowheads—pigment granules. Other explanations are given in the text

**Fig. 22:**
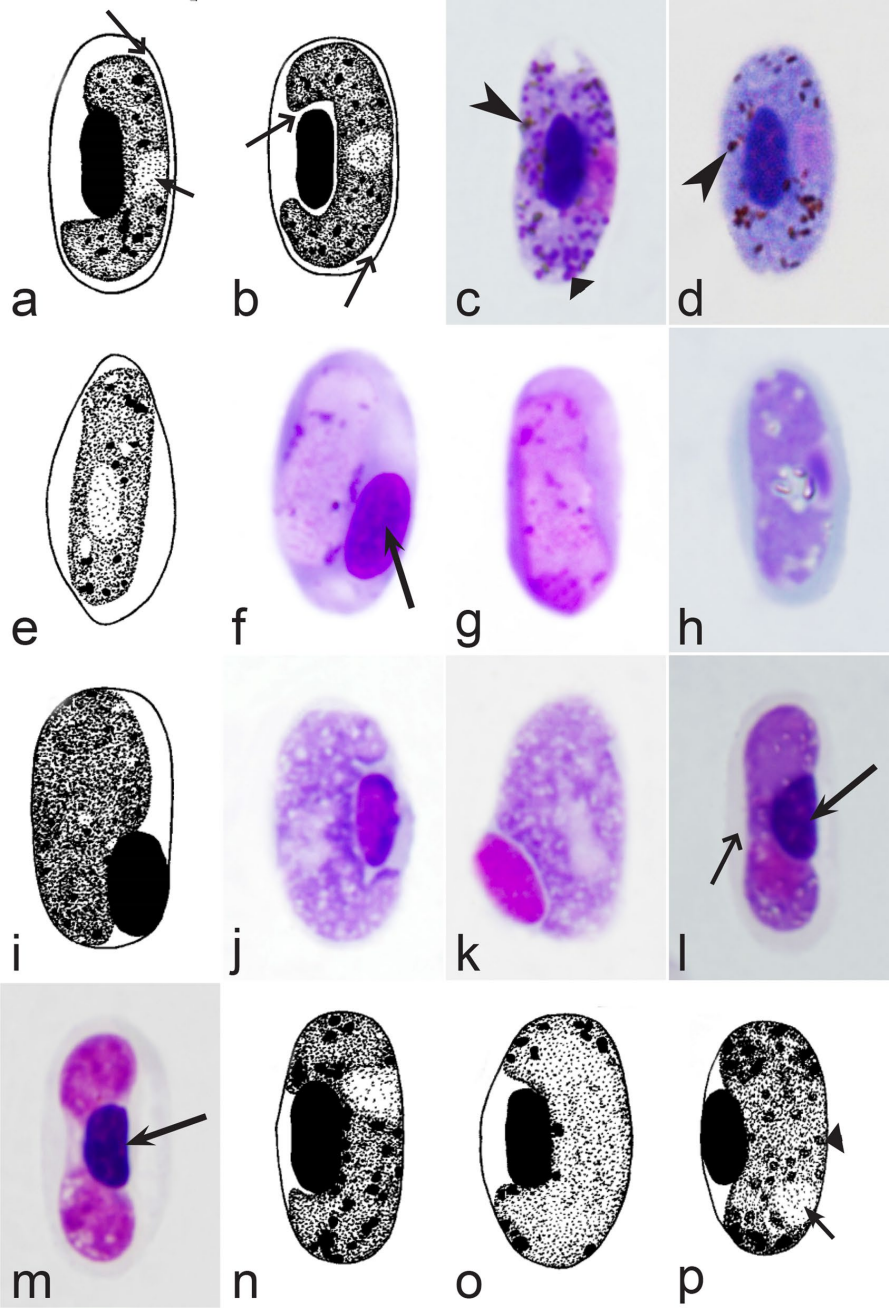
Morphological features of gametocytes, which are used for identification of *Haemoproteus* species parasitizing Piciformes birds. Macrogametocytes (**a**–**e**, **i**–**l**, **n**, **p**) and microgametocytes (**f**–**h**, **m**, **o**) of *Haemoproteus xantholaemae* (**a**), *H. cornuata* (**b**), *H. velans* (**c**), *H. homovelans* (**d**), *H. bennetti* (**e**–**g**), *H. bucconis* (**h**), *H. thereicerycis* (**i**–**k**), *H. bilobata* (**l**, **m**), *H. indicator* (**n**, **o**) and *H. borgesi* (**p**). Note that *H. velans* gametocytes contain numerous prominent volutin granules and/or distinct volutin clumps (**c**), but this is not the case in *H. homovelans* (**d**). The advanced gametocytes of *H. bennetti*, *H. bucconi*s and *H. thereicerycis* markedly displace nuclei of infected erythrocytes to the erythrocyte poles (**f**, **i**, **k**) and can enucleate the infected erythrocytes (**e**, **g**, **h**). Fully grown gametocytes of *H. bilobata* assume a unique dumbbell-like or bilobed form (**l**, **m**) and cause a readily visible flattening of nuclei of infected erythrocytes at the nuclei side, which is opposite to the gametocytes (**l**, **m**). Images **f**–**h**, **j**–**m** are from the type material, which is fading, resulting in pale staining and the poorly recognizable pigment granules and nuclei, however the overall form of the gametocytes is readily visible. Long simple arrows—host cell nuclei. Short simple arrows—parasite nuclei. Simple arrowheads—pigment granules. Simple wide long arrows—unfilled spaces between gametocytes and the envelope of infected erythrocytes or/and between gametocytes and nuclei of the erythrocytes. Triangle wide arrowhead—volutin granule. Other explanations are given in the text

**Fig. 23:**
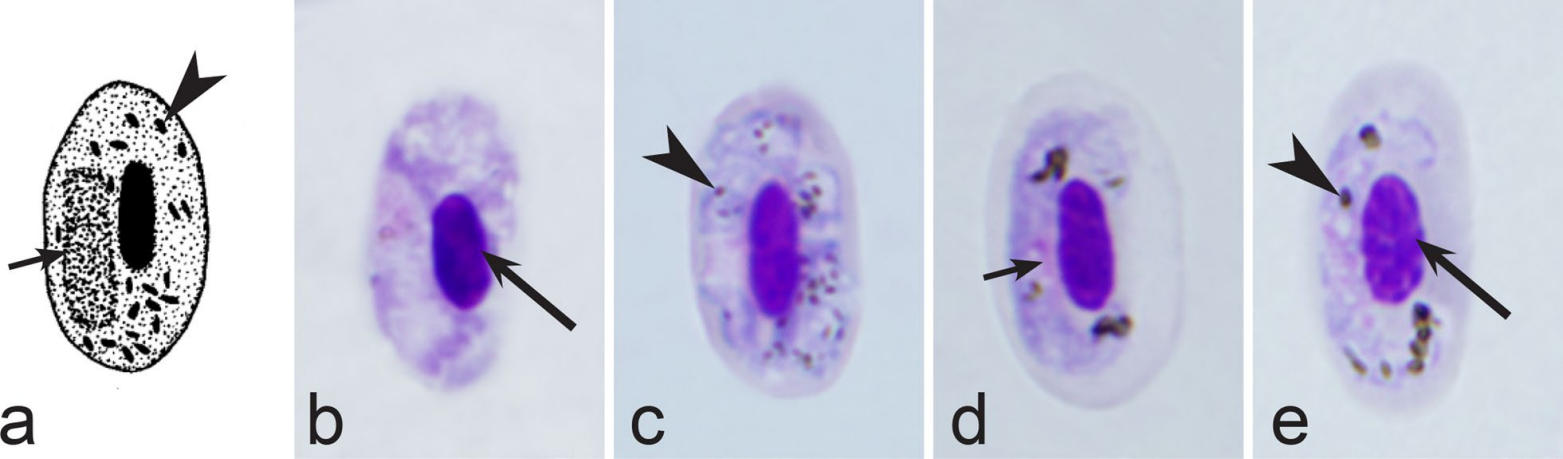
Morphological features of fully grown gametocytes, which are used for identification of *Haemoproteus* species parasitizing Psittaciformes birds. Microgametocyte (**a**) and macrogametocytes (**b**–**e**) of *Haemoproteus handai* (**a**, **b**), *H. homohandai* (**c**) and *H. psittaci* (**d**, **e**). Note the markedly different form, size and number of pigment granules in gametocytes of *H. handai* (**a**), *H. homohandai* (**c**) and *H. psittaci* (**e**). Image **b** is from the type material, which is fading, resulting in pale staining and the poorly recognizable pigment granules and nuclei, however the overall form of the gametocyte is readily visible. Long simple arrows—host cell nuclei. Short simple arrows—parasite nuclei. Simple arrowheads—pigment granules. Other explanations are given in the text

**Fig. 24:**
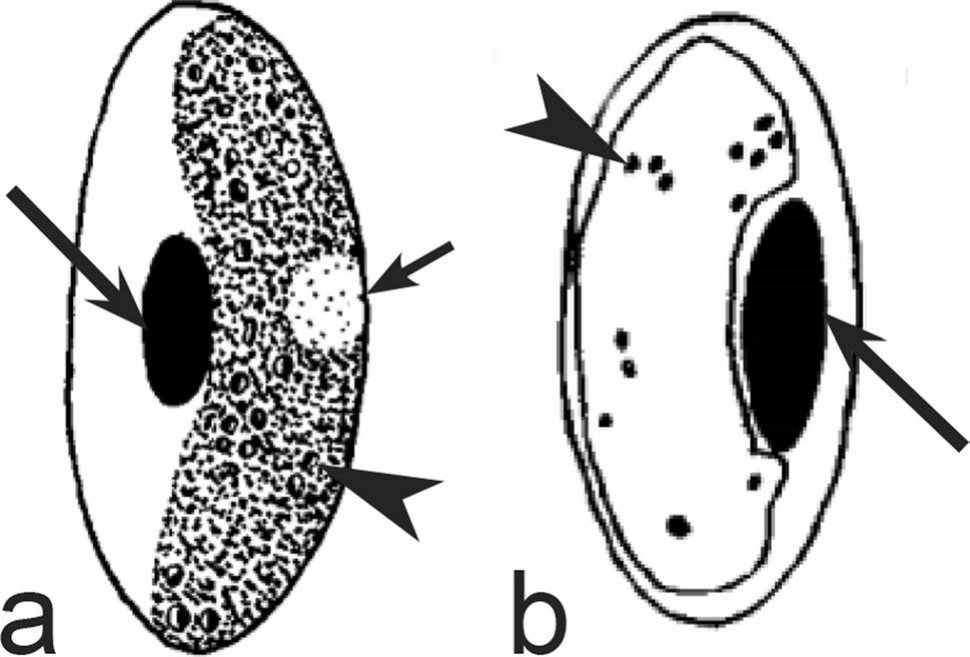
Morphological features of gametocytes, which are used for identification of *Haemoproteus* species parasitizing Pterocliformes birds. Macrogametocytes of *Haemoproteus krylovi* (**a**) and *H. pteroclis* (**b**). Long simple arrows—host cell nuclei. Short simple arrow—parasite nucleus. Simple arrowheads—pigment granules. Other explanations are given in the text

**Fig. 25:**
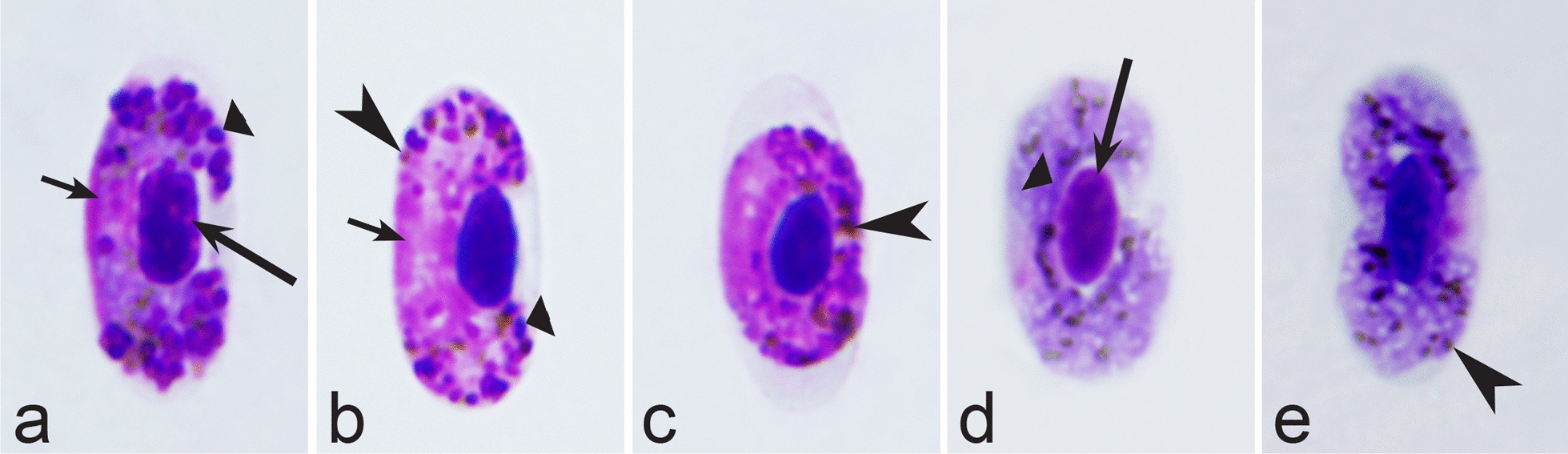
Morphological features of gametocytes, which are used for identification of *Haemoproteus* species parasitizing Strigiformes birds. Macrogametocytes (**a**, **d**, **e**) and microgametocytes (**b**, **c**) of *Haemoproteus syrnii* (**a**–**c**) and *H. noctuae* (**d**, **e**). Note that volutin in *H. syrnii* gametocytes is arranged in compact roundish or circular granules (**a**, **b**), but this is not a case in *H. noctuae* (**d**, **e**). Long simple arrows—host cell nuclei. Short simple arrows—parasite nuclei. Simple arrowheads—pigment granules. Triangle wide arrowheads—volutin. Other explanations are given in the text

**Fig. 26:**
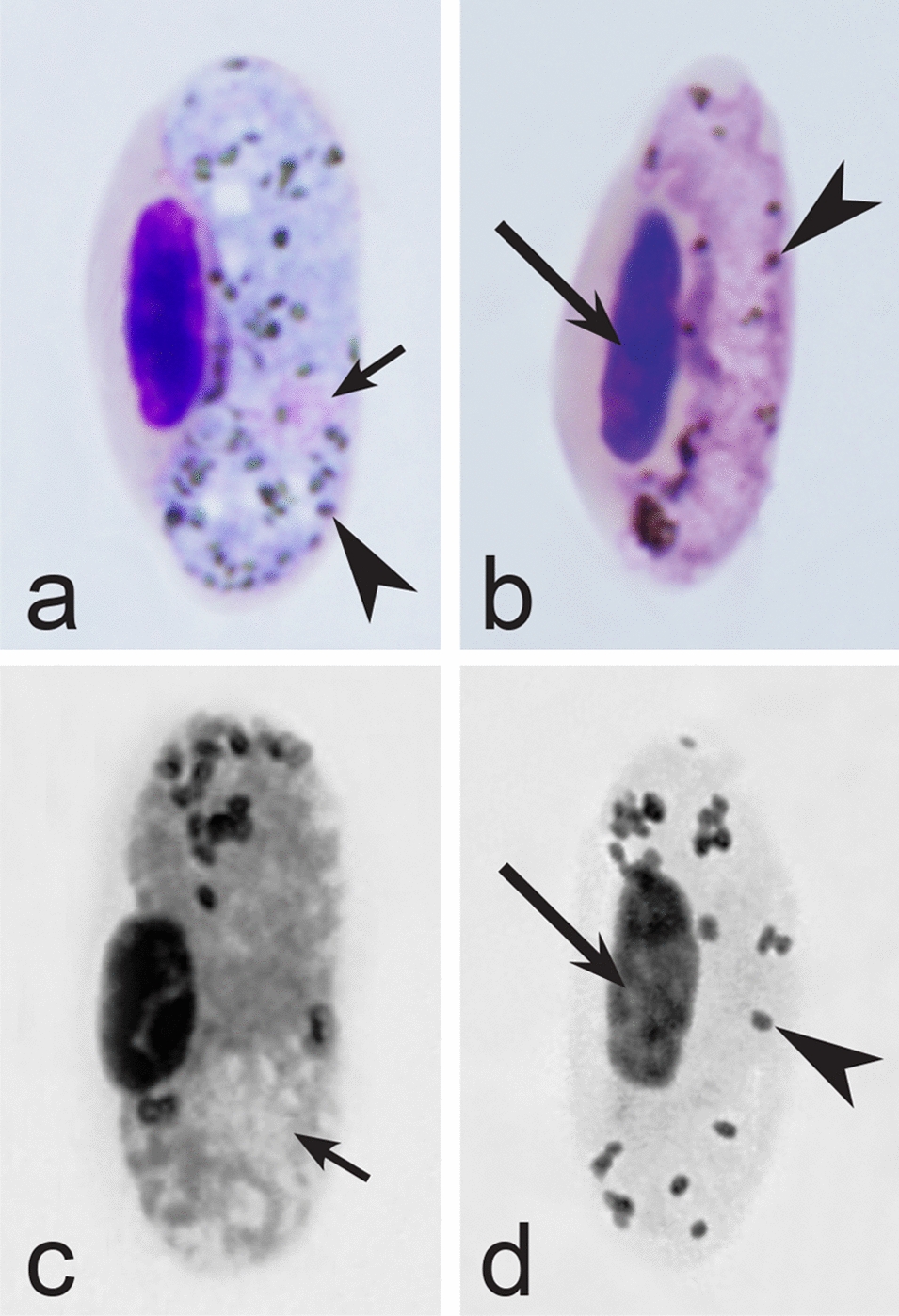
Morphological features of fully grown gametocytes, which are used for identification of *Haemoproteus* species parasitizing Suliformes birds. Macrogametocytes (**a**, **c**) and microgametocytes (**b**, **d**) of *Haemoproteus iwa* (**a**, **b**) and *H. valkiunasi* (**c**, **d**). Note that the number of pigment granules in fully grown *H. iwa* macrogametocytes (**a**) is at least twice that in fully grown microgametocytes (**b**), but this is not the case in *H. valkiunasi* (**c**, **d**). Long simple arrows—host cell nuclei. Short simple arrows—parasite nuclei. Simple arrowheads—pigment granules. Other explanations are given in the text

**Fig. 27:**
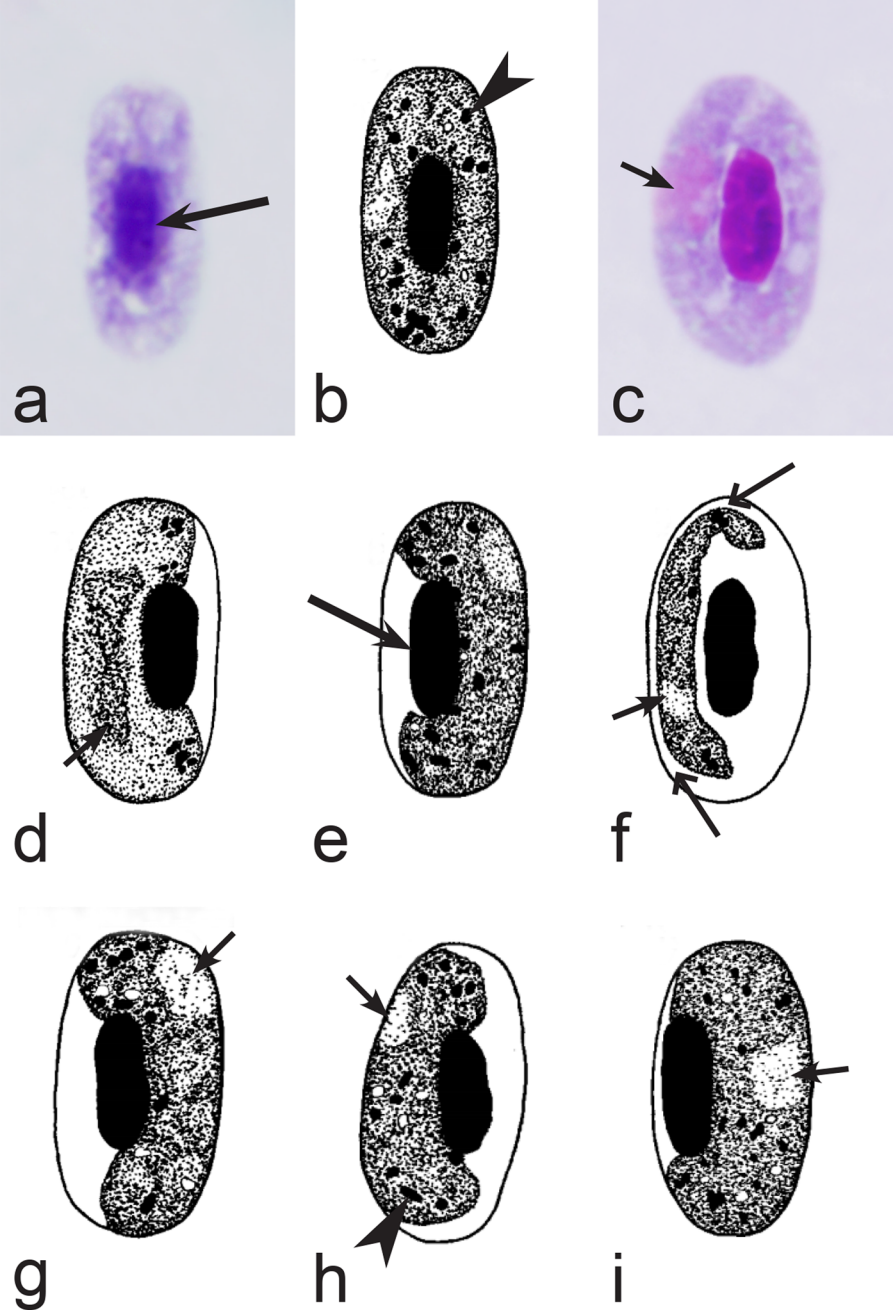
Morphological features of gametocytes, which are used for identification of *Haemoproteus* species parasitizing passeriform birds (suborder Tyranni) of the families Eurylaimidae, Furnariidae, Pittidae, Thamnophilidae, Tyrannidae. Macrogametocytes (**a**–**c, e**–**i**) and microgametocytes (**d**) of *H. circumnuclearis* (**a**, **b**), *H. pittae* (**c**, **d**), *H. tyranni* (**e**), *H. furnarius* (**f**, **g**), *H. formicarius* (**h**), *H. eurylaimus* (**i**). Note that attenuated (snake-like) advanced gametocytes (**f**) develop in *H. furnarius.* Image **a**, **c** are from the type material, which is fading, resulting in pale staining and the poorly recognizable pigment granules and nuclei, however the overall form of gametocytes is readily visible. Long simple arrows—host cell nuclei. Short simple arrows—parasite nuclei. Simple arrowheads—pigment granules. Simple wide long arrows—space between developing gametocyte and envelope of infected erythrocyte. Other explanations are given in the text

**Fig. 28:**
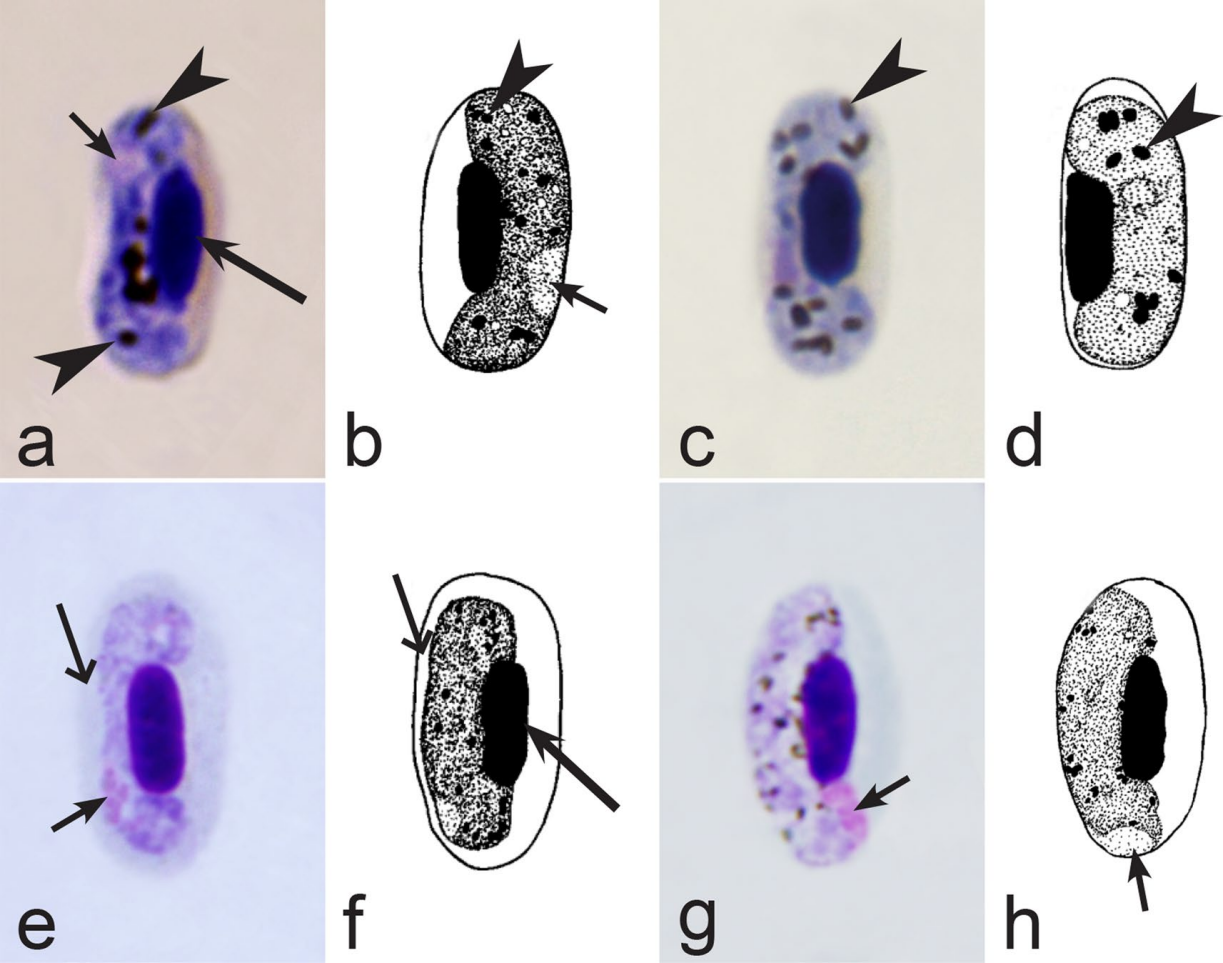
Morphological features of gametocytes, which are used for identification of *Haemoproteus* species parasitizing passeriform birds (suborder Passeri) of the families Meliphagidae, Oriolidae, Pachycephalidae, Vireonidae birds. Macrogametocytes of *H. vireonis* (**a**, **b**), *H. ptilotis* (**c**, **d**), *H. pachycephalus* (**e**, **f**) and *H. orioli* (**g**, **h**). Note that advanced growing gametocytes of *H. pachycephalus* (size greater than length of erythrocyte nuclei) do not touch the envelope of infected erythrocytes along their entire margin (**e**, **f**). Pigment granules are small in gametocytes of *H. orioli*, and nuclei assume terminal position in macrogametocytes of this parasite (**g**, **h**). Images **a**, **c**, **e** are from the type material, which is fading, resulting in pale staining (**e**) and the poorly recognizable nuclei (**a**, **c**, **e**) and pigment granules (**e**), however the overall form of the gametocytes is readily visible. Long simple arrows—host cell nuclei. Short simple arrows—parasite nuclei. Simple arrowheads—pigment granules. Simple wide long arrows—space between developing gametocyte and envelope of infected erythrocyte. Other explanations are given in the text

**Fig. 29:**
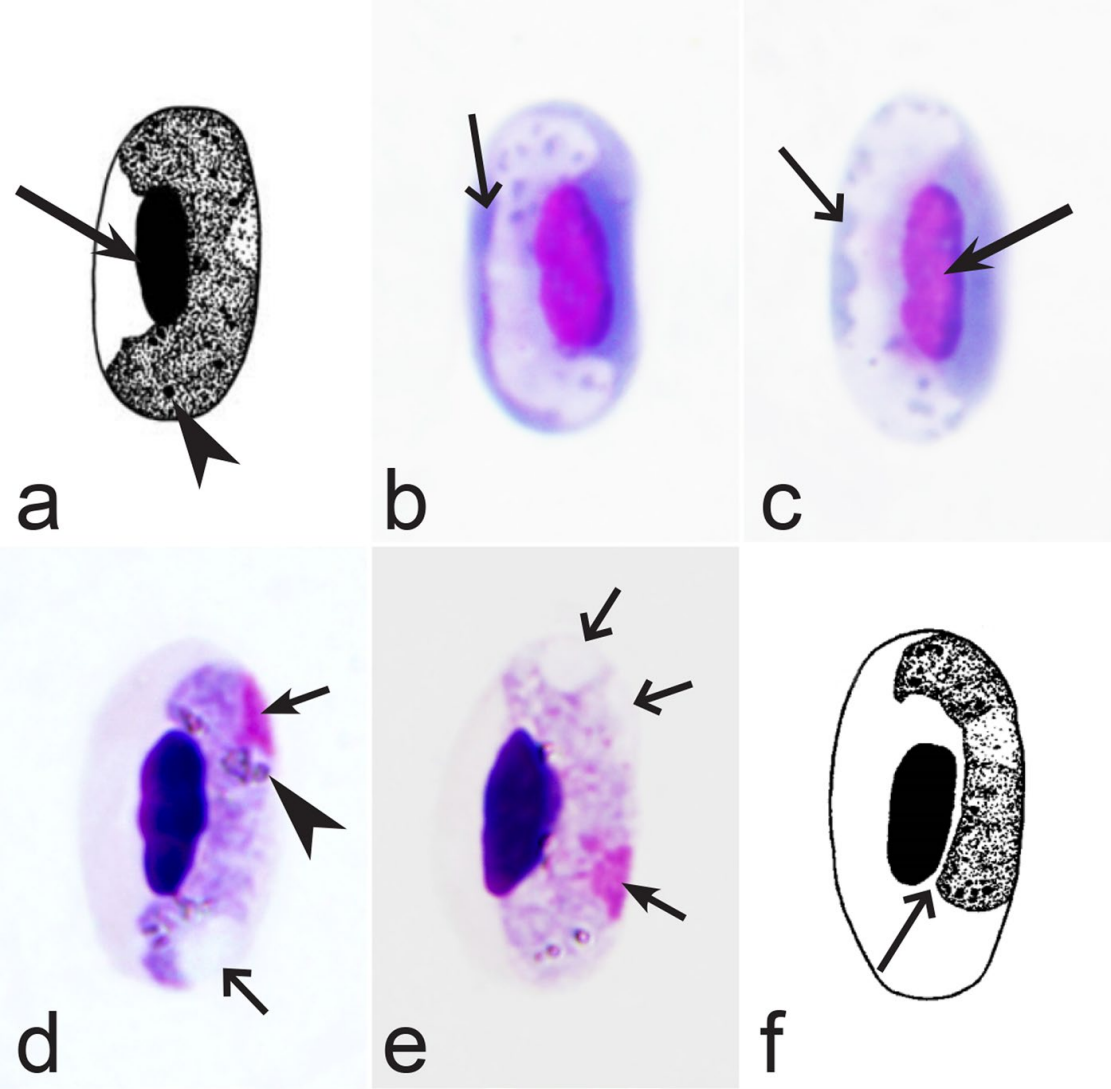
Morphological features of gametocytes, which are used for identification of *Haemoproteus* species parasitizing passeriform birds (suborder Passeri) of the families Aegithinidae, Artamidae, Malaconotidae, Vangidae birds. Macrogametocytes (**a**, **b**, **d**–**f**) and microgametocytes (**c**) of *H. aegithinae* (**a**–**c**), *H. bukaka* (**d**, **e**) and *H. cublae* (**f**). Note that advanced growing gametocytes of *H. aegithinae* are closely appressed to nuclei of infected erythrocytes but do not touch envelope of the erythrocytes along their entire margin (**b**, **c**). On the opposite, the advanced growing gametocytes of *H. cublae* do not touch the nuclei of infected erythrocytes along their entire margin but are closely appressed to erythrocyte envelope (**f**). Macrogametocytes of *H. bukaka* contain large vacuoles (**d**, **e**). Images **b**, **c** are from the type material, which is fading, resulting in pale staining and the poorly recognizable pigment granules and nuclei, however the overall form of gametocytes is readily visible. Long simple arrows—host cell nuclei. Short simple arrows—parasite nuclei. Simple arrowheads—pigment granules. Simple wide long arrows—space between developing gametocytes and envelope or nucleus of infected erythrocytes. Simple wide short arrows—vacuoles. Other explanations are given in the text

**Fig. 30:**
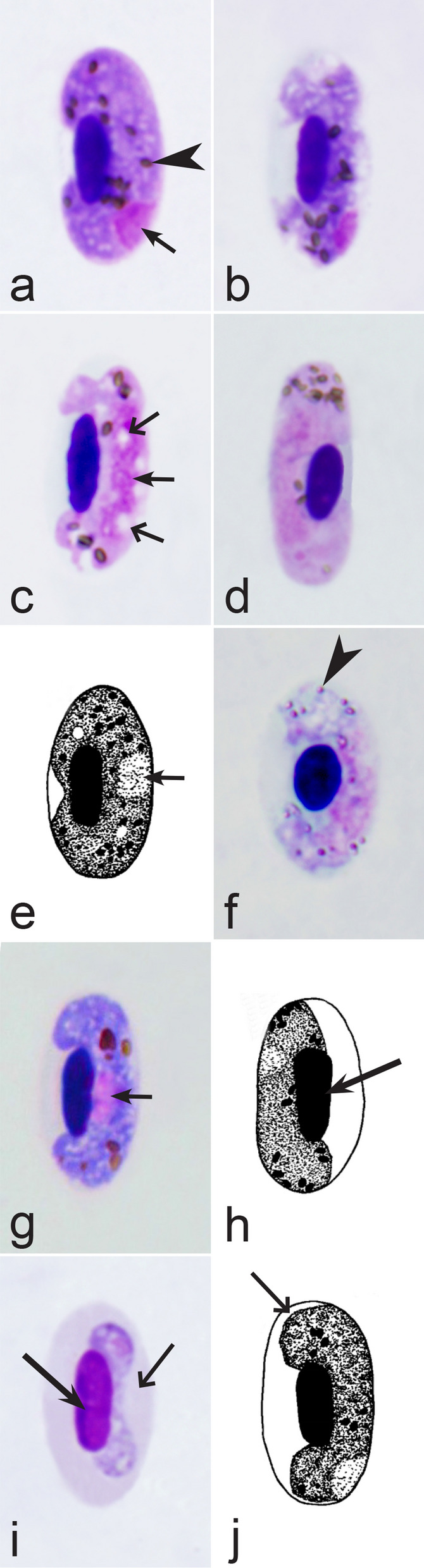
Morphological features of gametocytes, which are used for identification of *Haemoproteus* species parasitizing passeriform birds (suborder Passeri) of the families Corvidae, Dicruridae, Laniidae, Monarchidae birds. Macrogametocytes (**a**, **b**, **e**, **g**–**j**) and microgametocytes (**c**, **d**, **f**) of *H. homopicae* (**a**–**c**), *H. picae* (**d**), *H. danilewskii* (**e**, **f**), *H. lanii* (**g**), *H. dicruri* (**h**) and *H. monarchus* (**i**, **j**). Note that the cytoplasm is markedly vacuolated (**c**) in growing and fully grown microgametocytes of *H. homopicae*, but this is not a case (**d**) in microgametocytes of *H. picae.* Macrogametocyte nucleus is closely appressed to the nucleus of infected erythrocyte (**g**) in *H. lanii*. Growing gametocyte of *H. monarchus* is dumbbell-shaped and does not touch the envelope of erythrocyte along its entire margin (**i**). Image **i** is from the type material, which is fading, resulting in pale staining and the poorly recognizable pigment granules and nucleus, however the overall form of the gametocyte is readily visible. Long simple arrows—host cell nuclei. Short simple arrows—parasite nuclei. Simple arrowheads—pigment granules. Simple wide long arrows—spaces between gametocytes and envelope of infected erythrocytes. Simple wide short arrows—vacuoles. Other explanations are given in the text

**Fig. 31:**
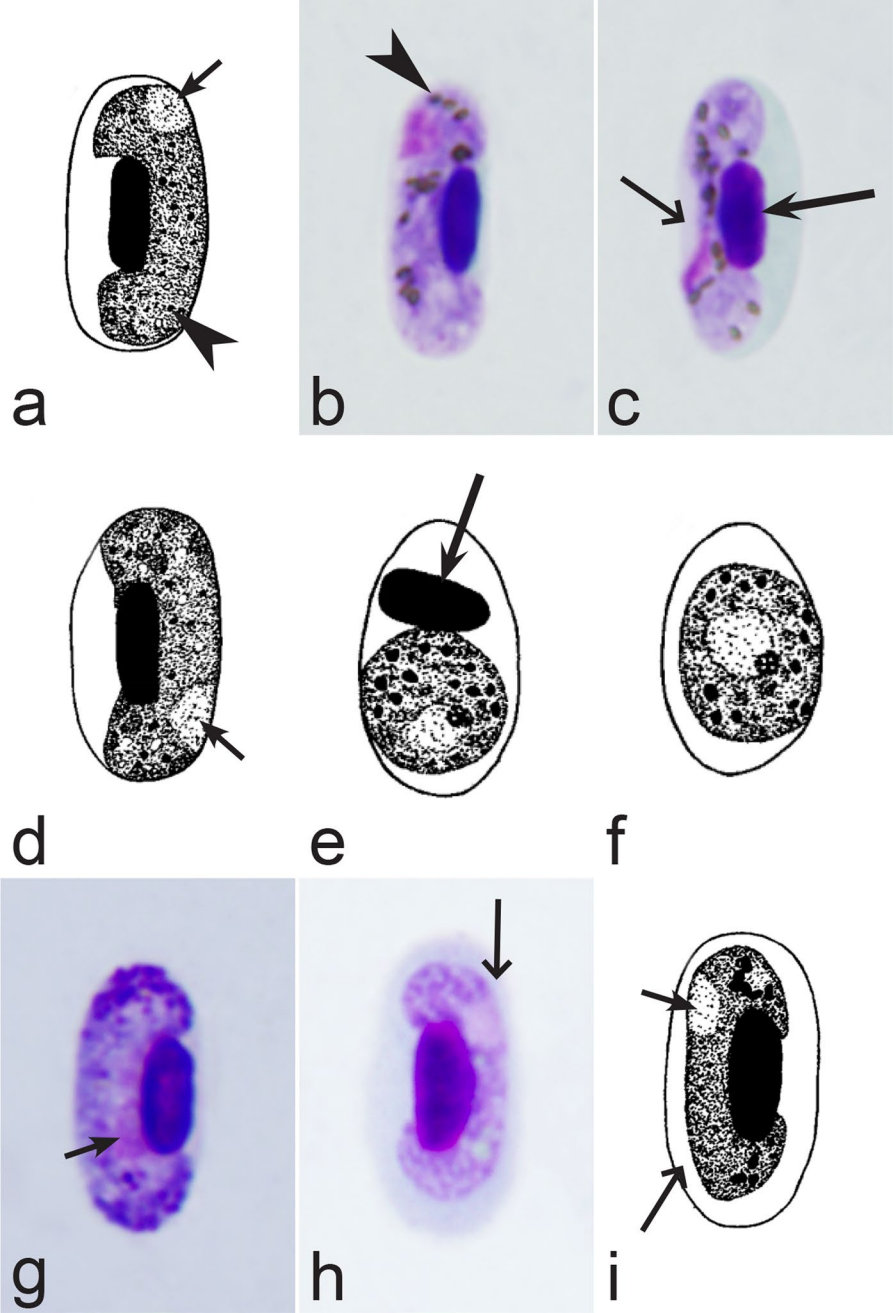
Morphological features of gametocytes, which are used for identification of *Haemoproteus* species parasitizing passeriform birds (suborder Passeri) of the families Alaudidae, Cisticolidae, Melanocharitidae, Paridae birds. Macrogametocytes of *H. wenyoni* (**a**), *H. majoris* (**b**, **c**), *H. alaudae* (**d**), *H. parus* (**e**, **f**), *H. calandrellae* (**g**), *H. nucleophilus* (**h**, **i**). Note that the growing gametocyte of *H. majoris* assume dumbbell-like shape (**c**)*.* Fully grown gametocytes of *H. parus* are roundish in form (**e**, **f**). Macrogametocyte nucleus assume median positions and adheres to the infected erythrocyte nucleus (**g**) in *H. calandrellae*. Fully grown gametocytes are closely appressed to the nuclei of erythrocytes but do not touch the envelope of the erythrocytes along their entire margin (**h, i**) in *H. nucleophilus.* Image **h** is from the type material, which is fading, resulting in pale staining and the poorly recognizable pigment granules and nucleus, however the overall form of the gametocyte is readily visible. Long simple arrows—host cell nuclei. Short simple arrows—parasite nuclei. Simple arrowheads—pigment granules. Simple wide long arrows—spaces between gametocytes and envelope of infected erythrocytes. Other explanations are given in the text

**Fig. 32:**
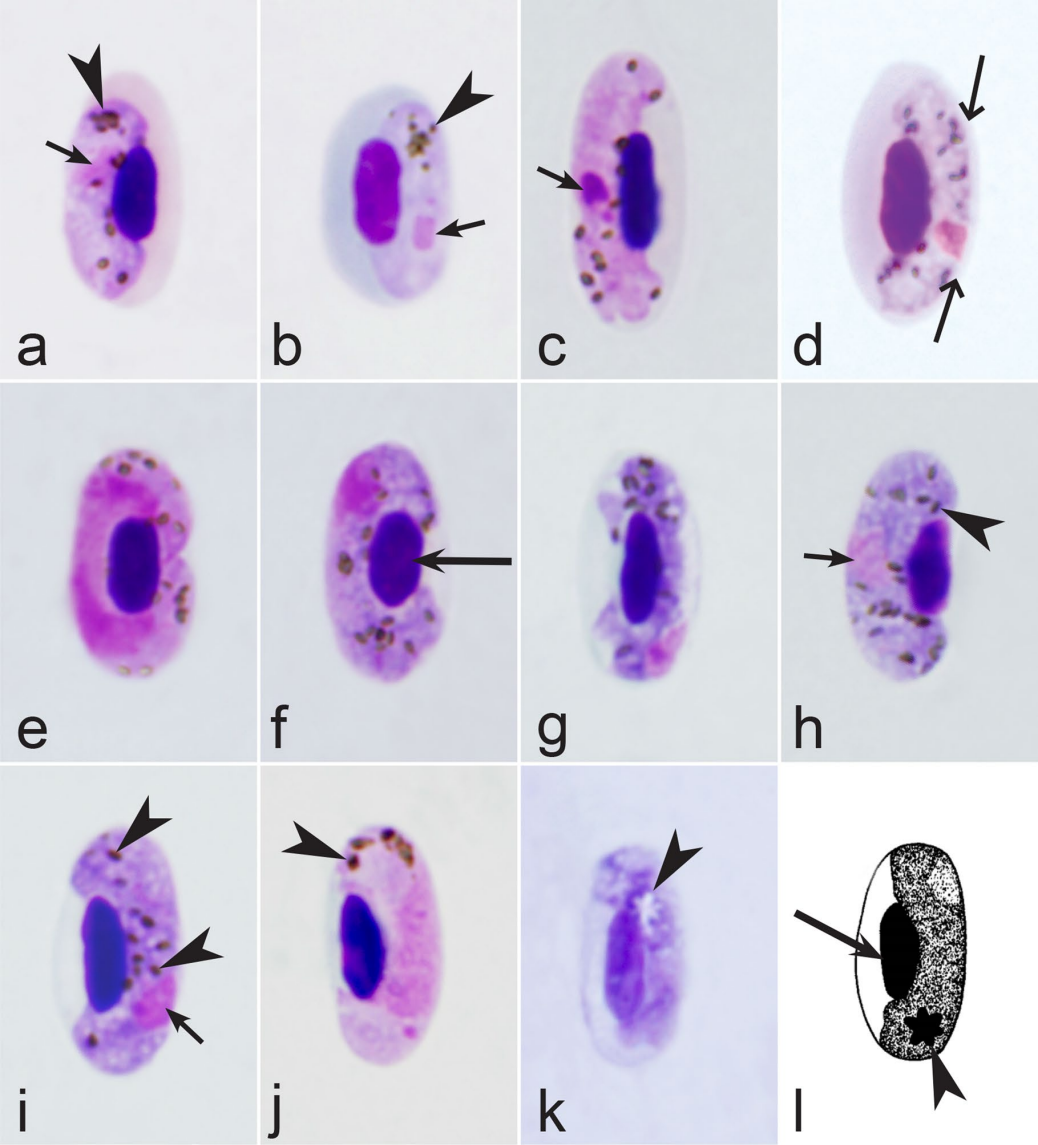
Morphological features of gametocytes, which are used for identification of *Haemoproteus* species parasitizing passeriform birds (suborder Passeri) of the families Acrocephalidae and Hirundinidae birds. Macrogametocytes (**a**, **d, f**–**i**, **k**, **l**) microgametocytes (**b**, **c**, **e**, j) of *H. payevskyi* (**a**, **b**), *H. nucleocondensus* (**c**, **d**), *H. belopolskyi* (**e**–**g**), *H. parahirundinis* (**h**), *H. hirundinis* (**i**, **j**) and *H. stellaris* (**k**, **l**). Note the markedly condensed nuclear material in fully grown microgametocytes of *H. payevskyi* (**b**) and *H. nucleocondensus* (**c**), resulting in similar size of the nuclei in microgametocytes (**b, c**) and macrogametocytes (**a**, **d**), a rare character in *Haemoproteus* parasites. Macrogametocytes nucleus is median in position (**h**) in *H. parahirundinis,* but this is not a case in *H. hirundinis* (**i**). Gigantic pigment granules (**k**, **l**) are present in fully grown gametocytes of *H. stellaris;* the granules might be arranged like stars (**k**, **l**). Image **k** is from the type material, which is fading, resulting in pale staining and the poorly recognizable pigment granules and nucleus, however the overall form of the gametocyte and pigment granules are readily visible. Long simple arrows—host cell nuclei. Short simple arrows—parasite nuclei. Simple arrowheads—pigment granules. Simple wide long arrows—spaces between gametocytes and envelope of infected erythrocytes. Other explanations are given in the text

**Fig. 33:**
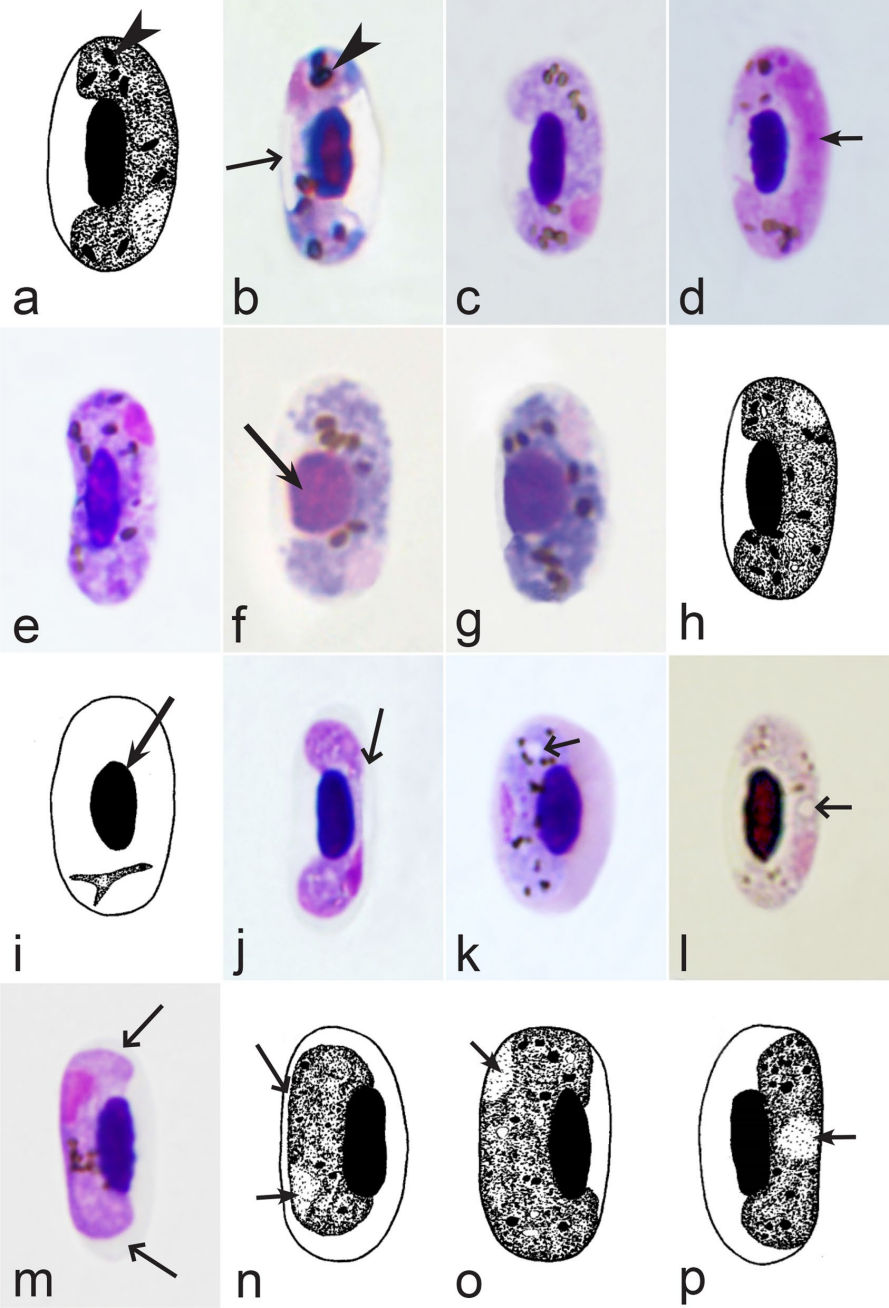

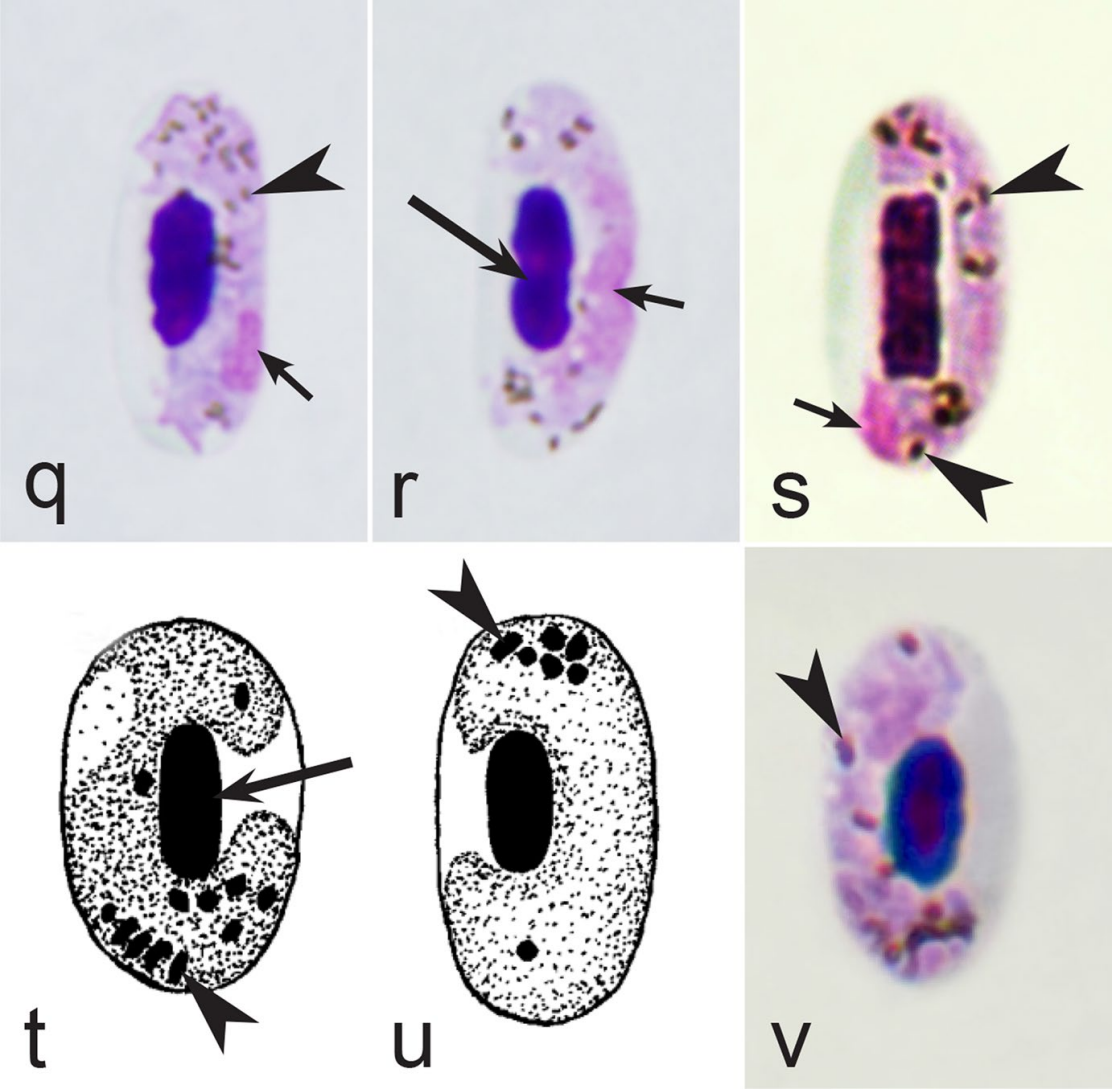
Morphological features of gametocytes, which are used for identification of *Haemoproteus* species parasitizing passeriform birds (suborder Passeri) of the families Leiothrichidae, Phylloscopidae, Pycnonotidae, Sylviidae and Zosteropidae birds. Macrogametocytes (**a**–**c**, **e**–**h**, **j**–**q**, **s**, **t**, **v**), microgametocytes (**d**, **r**, **u**) and young gametocyte (**i**) of *H. killangoi* (**a**, **b**), *H. parabelopolskyi* (**c**–**e**), *H. leiothrichus* and *H. homoleiothrichus* (**f**, **g**), *H. timalus* (**h**, **i**), *H. philippinensis* (**j**), *H. vacuolatus* (**k**), *H. palloris* (**l**), *H. homogeneae* (**m**), *H. otocompsae* (**n**, **o**), *H. sanguinis* (**p**), *H. pallidulus* (**q**, **r**), *H. homopalloris* (**s**) and *H. zosteropis* (**t-v**). Note that dumbbell-shaped gametocyte does not touch envelope of infected erythrocyte along its entire margin (**j**) in *H. philippinensis.* The majority of advanced macrogametocytes contain a clear roundish discrete vacuole (**k, l**) in *H. vacuolatus* and *H. palloris.* The cytoplasm is homogenous in appearance (**m**) in macrogametocytes of *H. homogeneae.* Advanced growing macrogametocytes of *H. otocompsae* and *H. sanguinis* are closely appressed to the nuclei of infected erythrocytes but do not touch the envelope of the erythrocytes along their entire margin (**n**). The macrogametocyte cytoplasm is relatively pale-stained (**q**) and is similar to microgametocyte (**r**) based this character. Fully grown gametocytes of *H. killangoi* and *H. zosteropis* contain large size (greater than 1 µm) pigment granules (**a**, **b**, **t**–**v**). Images **j**, **v** are from the type material, which is fading, resulting in pale staining and the poorly recognizable pigment granules and nuclei, however the overall form of the gametocytes is readily visible. Long simple arrows—host cell nuclei. Short simple arrows—parasite nuclei. Simple arrowheads—pigment granules. Simple wide long arrows—spaces between gametocytes and envelope of infected erythrocytes. Simple wide short arrows—vacuoles. Other explanations are given in the text

**Fig. 34:**
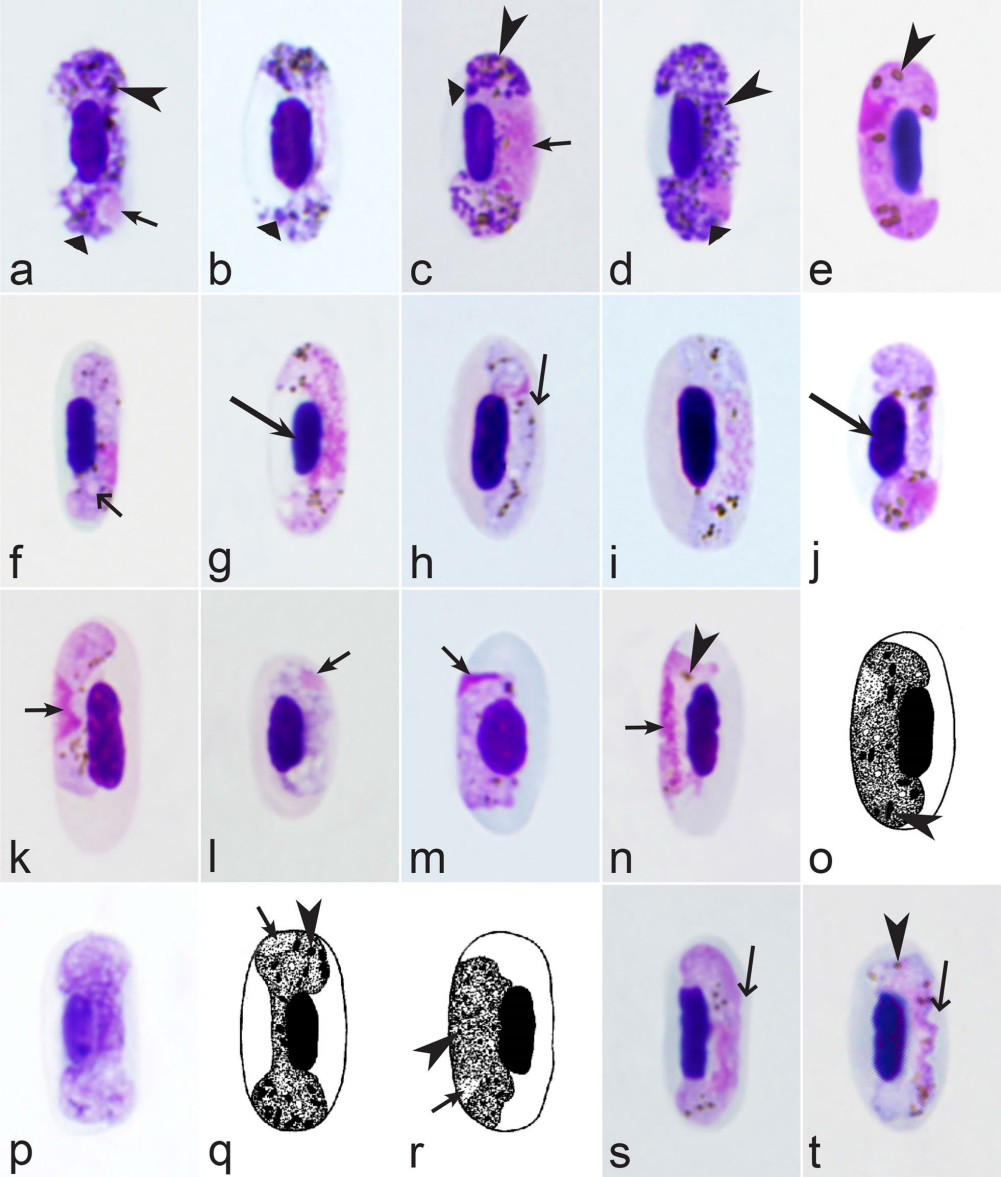
Morphological features of gametocytes, which are used for identification of *Haemoproteus* species parasitizing passeriform birds (suborder Passeri) of the families Mimidae, Muscicapidae, Sittidae, Sturnidae and Turdidae birds. Macrogametocytes (**a**, **d**–**f**, **h**, **j**–**m**, **o**–**r**) and microgametocytes (**b**, **c**, **g**, **i**, **n**, **s**, **t**) of *H. attenuatus* (**a**, **b**), *H. balmorali* (**c**, **d**), *H. pastoris* (**e**), *H. homominutus* (**f**, **g**), *H. kairullaevi* (**h**, **i**), *H. sittae* (**j**), *H. asymmetricus* (**k**), *H. fallisi* (**l**), *H. minutus* (**m**, **n**), *H. beckeri* (**o**), *H. neseri* (**p**, **q**), *H. nipponensis* (**r**) and *H. pallidus* (**s**, **t**). Note that advanced growing microgametocytes of *H. attenuatus* are markedly narrow (attenuated) in width (**b**). Gametocytes of *H. attenuatus* and *H. balmorali* are overfilled with volutin granules (**a**–**d**). The asymmetrical position of advanced growing gametocytes in regard of erythrocyte nuclei (**k**) is a characteristic feature of *H. asymmetricus*. Advanced growing gametocytes of *H. neseri* (**p**, **q**) have well-pronounces dumbbell-like form (**p**, **q**). Fully grown gametocytes of *H. pallidus* are closely appressed to the nuclei of infected erythrocytes, but do not touch the erythrocyte envelope along their entire margin (**s**, **t**). Images **l**, **p** are from the type material, which is fading, resulting in pale staining and the poorly recognizable pigment granules and nucleus, however the overall form of the gametocytes is readily visible. Long simple arrows—host cell nuclei. Short simple arrows—parasite nuclei. Simple arrowheads—pigment granules. Simple wide long arrows—spaces between gametocytes and envelope of infected erythrocytes. Triangle wide arrowheads—volutin granules. Simple wide short arrow—vacuole. Other explanations are given in the text

**Fig. 35:**
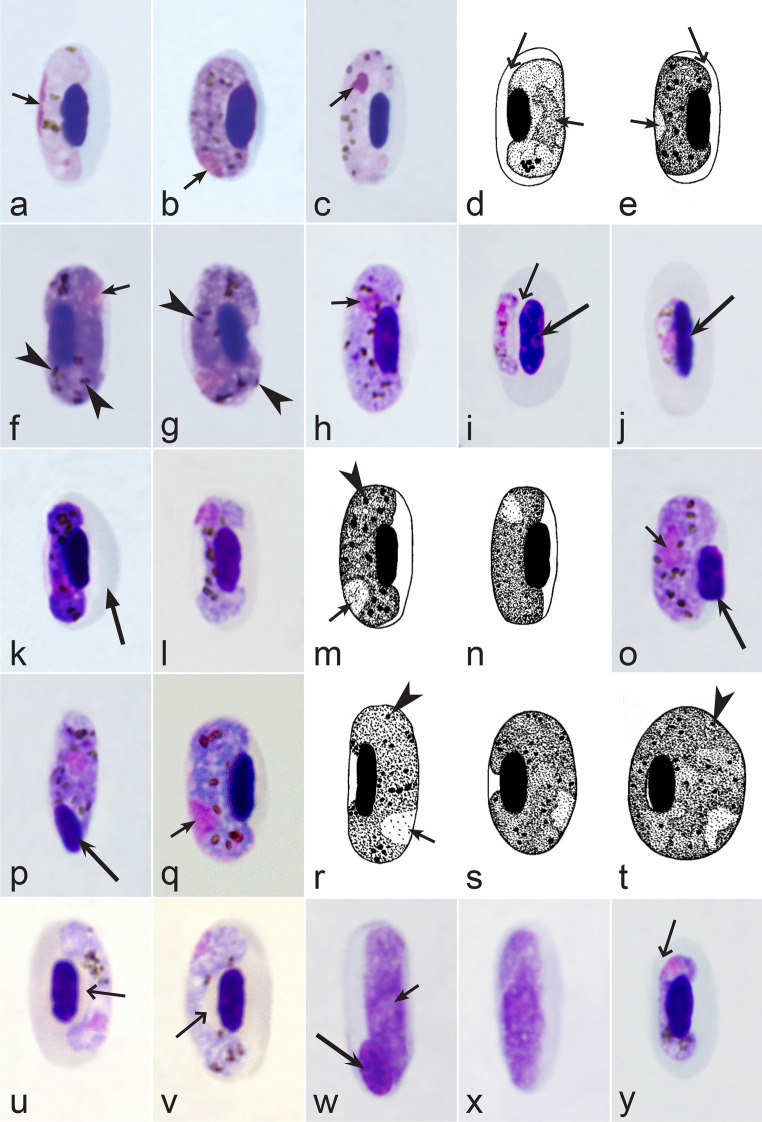

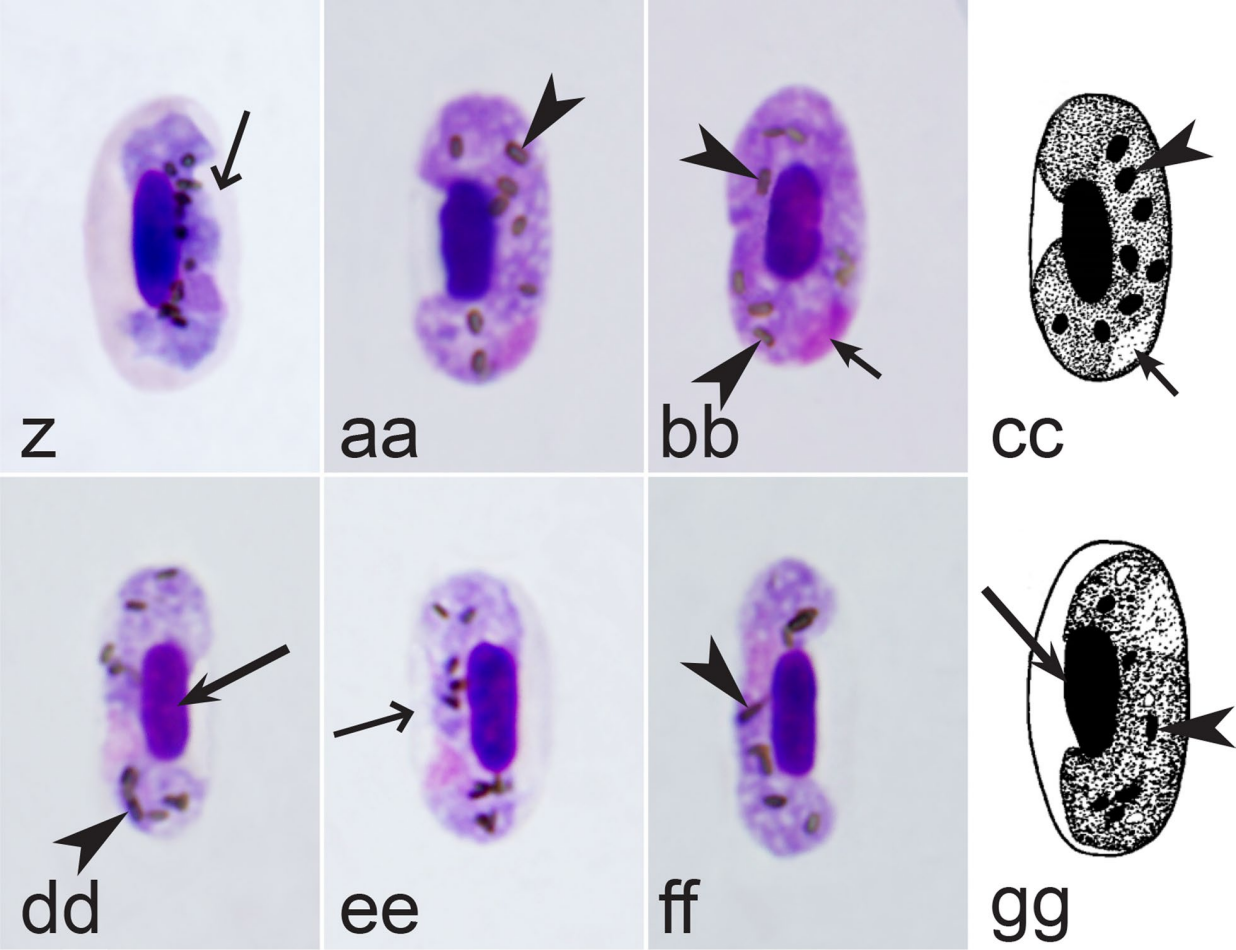
Morphological features of gametocytes, which are used for identification of *Haemoproteus* species parasitizing passeriform birds (suborder Passeri) of the families Dicaeidae, Estrildidae, Fringillidae, Motacillidae, Nectariniidae, Passeridae and Ploceidae birds. Macrogametocytes (**b**, **e**–**h**, **k**–**y**, **z**–**gg**), microgametocytes (**a**, **c**, d), and young gametocytes (**i**, **j**) of *H. nucleofascialis* (**a**, **b**), *H. micronuclearis* (**c**), *H. africanus* (**d**, **e**), *H. homobelopolskyi* (**f**, **g**), *H. passeris* (**h**, **i**), *H. fringillae* (**j**, **k**), *H. dolniki* (**l**), *H. queleae* (**m**), *H. dicaeus* (**n**), *H. tartakovskyi* (**o**, **p**), *H. anthi* (**q**), *H. orizivorae* (**r**), *H. globulosus* (**s**, **t**), *H. concavocentralis* (**u**, **v**), *H. uraeginthus* (**w, x**), *H. paranucleophilus* (**y**), *H. cyanomitrae* (**z**), *H. magnus* (**aa**, **bb**), *H. macropigmentatus* (**cc**), *H. motacillae* (**dd**–**ff**) and *H. bubalornis* (**gg**). Note that fully grown microgametocytes of *H. nucleofascialis* and *H. micronuclearis* contain markedly compressed (not diffuse) nuclei (**a**, **c**). Nucleus of fully grown *H. africanus* macrogametocyte locates centrally (**e**). The rod-like (thin) pigment granules (**f**, **g**) are common in advanced and fully grown gametocytes of *H. homobelopolskyi*. During growth, the young gametocytes of *H. passeris* first adhere to envelope of erythrocytes and then grow towards the erythrocyte nuclei (**i**), but the opposite pattern of growth (**j**) is characteristic in *H. fringillae.* Growing advanced macrogametocyte of *H. fringillae* is dumbbell-shaped and assume a distinct linear form; the parasite deforms infected erythrocytes by causing the envelop protrusion, which is located in the non-invaded cytoplasmic region of the erythrocyte (**k**). A readily distinguishable space is present between the growing advanced gametocyte of *H. concavocentralis* and the nucleus of infected erythrocyte, resulting in the gametocyte concave form (**u**, **v**). Fully grown gametocytes of *H. uraeginthus* often assume rhabdosomal form (**w**, **x**) and enucleate infected erythrocytes (**x**). Fully grown gametocytes of *H. paranucleophilus* are closely appressed to the nuclei of infected erythrocytes but do not touch the envelope of the erythrocytes along their entire margin (**y**). Advanced growing gametocytes of *H. cyanomitrae* are closely appressed to the nuclei of infected erythrocytes but often do not touch the envelope of the erythrocytes along their entire margin (**z**); the similar feature is characteristic of *H. sequeirae* (not shown). Fully grown gametocytes of *H. magnus*, *H. macropigmentatus*, *H. motacillae* and *H. bubalornis* contain the large-size pigment granules (**aa**–**cc**, **ff**, **gg**). Images **w**, **x** are from the type material, which is fading, resulting in pale staining and the poorly recognizable pigment granules and nucleus, however the overall form of the gametocytes is readily visible. Long simple arrows—host cell nuclei. Short simple arrows—parasite nuclei. Simple arrowheads—pigment granules. Simple wide long arrows—spaces between gametocytes and structures of infected erythrocytes. Simple wide short arrows—vacuoles. Triangle long arrow—protrusion of the erythrocyte envelope. Other explanations are given in the text

**Fig. 36:**
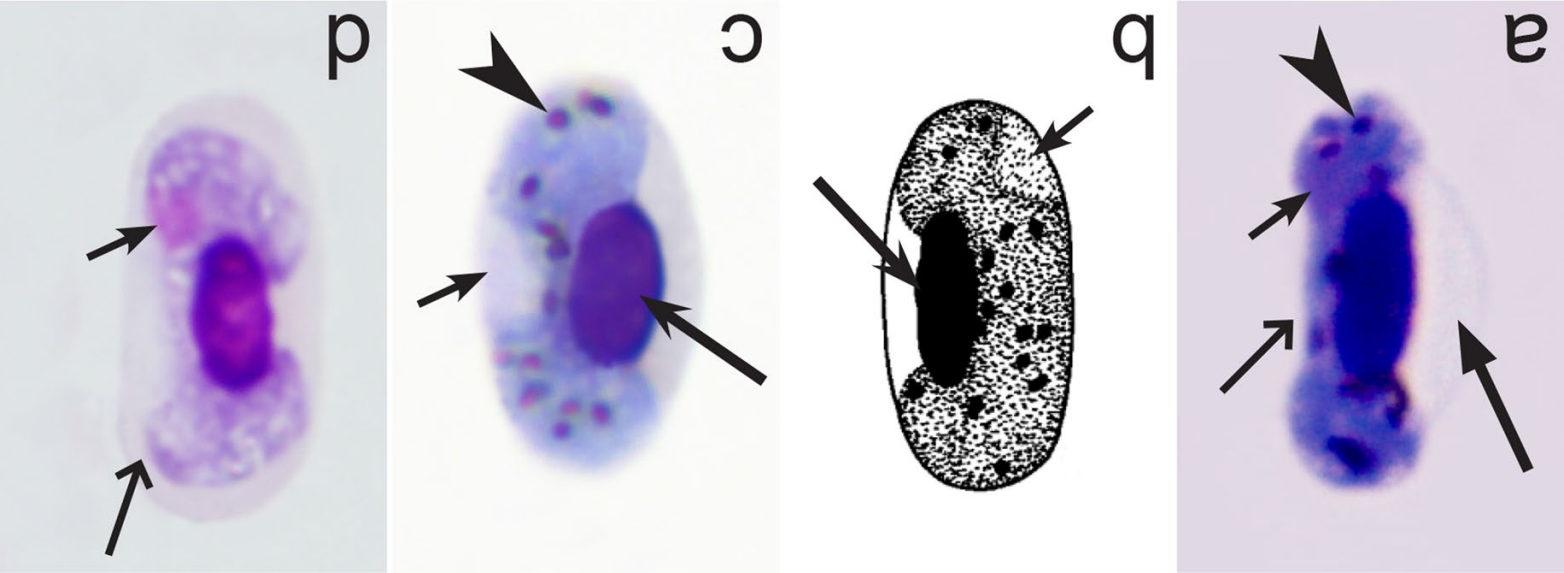
Morphological features of gametocytes, which are used for identification of *Haemoproteus* species parasitizing passeriform birds (suborder Passeri) of the families Emberizidae, Icteridae, Parulidae, Passerellidae and Thraupidae birds. Macrogametocytes of *H. erythrogravidus* (**a**), *H. coatneyi* (**b**), *H. nucleocentralis* (**c**) and *H. quiscalus* (**d**). Note that the fully grown gametocyte of *H. erythrogravidus* markedly deform the host cell by causing a balloon-like protrusion (**a**), which is located in the non-invaded cytoplasmic region of the erythrocytes. The nuclei assume predominantly central position (**c**) in fully grown macrogametocytes of *H. nucleocentralis*. Advanced dumbbell-shaped macrogametocytes, which do not touch envelope of erythrocytes along their entire margin (**d**) is a distinctive character of *H. quiscalus.* Image **d** is from the type material, which is fading, resulting in pale staining and the poorly recognizable pigment granules and nucleus, however the overall form of the gametocyte is readily visible. Long simple arrows—host cell nuclei. Short simple arrows—parasite nuclei. Simple arrowheads—pigment granules. Simple wide long arrows—spaces between gametocytes and the envelop of erythrocytes. Triangle long arrow—protrusion of the erythrocyte envelope. Other explanations are given in the text

Third, the morphometric characters of *Haemoproteus* gametocytes and their host cells can be similar and might overlap in some parasite species. Thus, they should be carefully considered during parasite identification and description. It is important to note that morphometric characters are functions of gametocyte size (age). In other words, the length and shape of gametocytes as well as number of pigment granules of the same parasite species can change markedly as the gametocytes grow and mature. As a result, the morphometric data are considered taxonomically valuable only if the gametocytes measurements are standardized and accessed only in fully grown gametocytes, which selection requires some experience and good quality preparations. Minor morphometric differences might be a result of incorrect measurements and are usually of low taxonomic value [3]. Furthermore, it is important to note that measurement of parasites is time consuming and requires subsequent statistical evaluation. This is why the use of morphometric characters was minimized in the keys. However, some readily distinguishable morphometric data (the nuclear displacement ratio, number of pigment granules and their size, etc.) were essential for some species identifications, but they were provided only for fully grown gametocytes in the keys. Measurements of the growing gametocytes will distort taxonomic values of the morphometric characters and might abate their applicability during species identifications and thus should be discouraged.

**Fig. 37:**
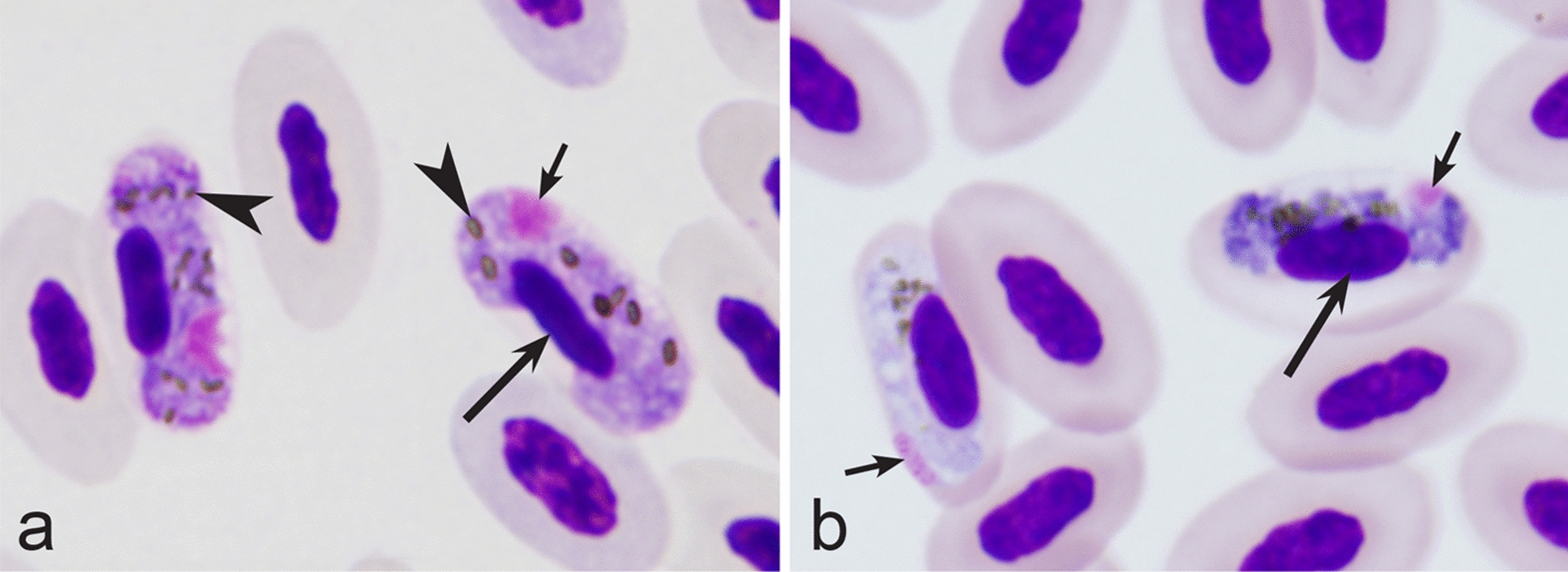
Examples of co-infections of two different species of *Haemoproteus* in same blood films. Co-infection of *Haemoproteus fringillae* (**a**, on the left) and *Haemoproteus magnus* (**a**, on the right) from the blood of common chaffinch *Fringilla coelebs*. Co-infection of *Haemoproteus pallidus* (**b**, on the left) and *Haemoproteus balmorali* (**b**, on the right) from the blood of European pied flycatcher *Ficedula hypoleuca.* Macrogametocytes of these species were shown. Note that *H. fringillae* and *H. magnus* can be readily distinguished due to different form and size of pigment granules (**a**). The cytoplasm of *H. balmorali* macrogametocyte is densely stained and contains numerous volutin granules, which are not a case in *H. pallidus* (**b**). Long simple arrows—host cell nuclei. Short simple arrows—parasite nuclei. Simple arrowheads—pigment granules. Other explanations are given in the text

Fourth, the co-infections of *Haemoproteus* and *Plasmodium* species often occur in naturally infected birds and require some experience to distinguish between co-existing parasites [35, 38, 39, 182]. The co-infections of several morphologically similar species belonging to the same genus might be particularly difficult to distinguish; such co-infections also often are hardly distinguishable by commonly used PCR-based methods, which apply general primers for the parasite detection [38, 40]. However, the co-infections of many *Haemoproteus* and other haemosporidian species belonging to same genus are readily distinguishable using morphological characters of blood stages (for example see Fig. 37a, b).

**Fig. 38:**
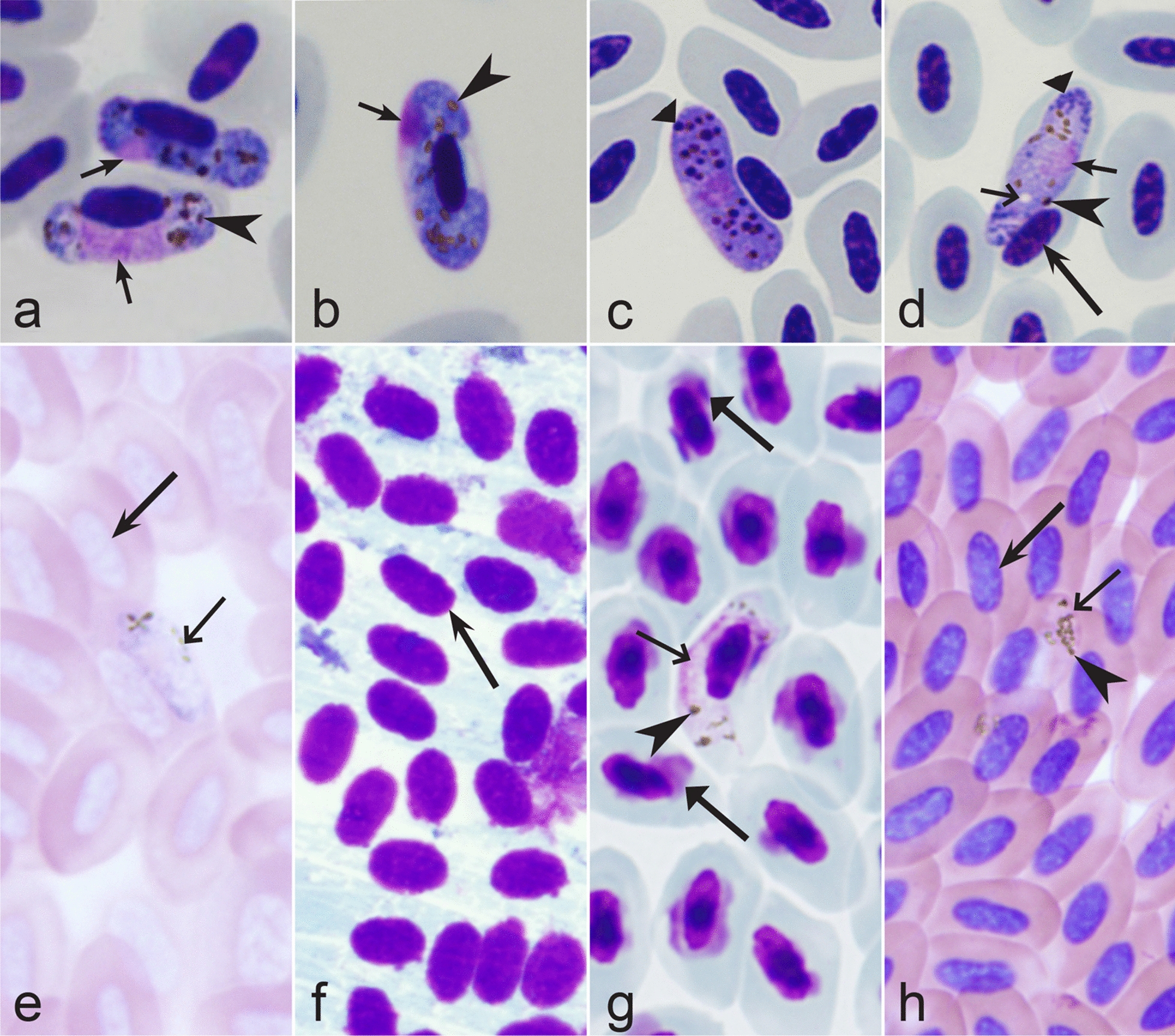
Gametocytes of *Haemoproteus* parasites as they are seen in good-quality (**a-d**) and bad-quality (**e**–**h**) blood films. The gametocyte structures (nuclei, pigment granules, volutin granules and position of the parasites in erythrocytes) are readily visible in good-quality blood films (**a**–**d**), but are hardly visible in preparations affected by incorrect procedures of staining (**e**), fixation (**f**, **g**) and blood film preparation (**h**). Note thick blood film (**h**), bad staining (**e**–**h**) and fixation (**f**, **g**), resulting in hardly visible parasite structures (**e**, **g**, **h**), destroyed host cell nuclei (**g**) and envelops (**f**). Bad-quality preparations are useless for haemosporidian species identification. Long simple arrows—host cell nuclei. Short simple arrows—parasite nuclei. Simple arrowheads—pigment granules. Simple wide short arrow—vacuole. Triangle wide arrowheads—volutin granules. Simple wide long arrows—*Haemoproteus* sp. parasites as they seen in bad-quality blood films. Triangle wide long arrows—artefacts of fixation, resulting in destroyed erythrocyte nuclei. Other explanations are given in the text

Fifth, the good quality of blood films is essential for the visualization of gametocyte morphological characters, which are needed for identification (Fig. 38a–d), but this is hardly possible in thick blood films (Fig. 38h) or in preparations affected by incorrect procedures of their drying, fixation, staining or storage [188]. Insufficient quality of preparations (Fig. 37e–h) is a significant obstacle in haemosporidian parasite species identification and description. It is essential to master methods of blood film preparation and storage before sample collection. These procedures are simple, relatively cheap and can be accomplished in any laboratory by careful application of well-described routine protocols [1, 3, 188, 189].

Sixth, the mature gametocytes of *Haemoproteus* parasites change morphology (round up and tend to escape from host cells) within one minute after the exposure to air [3, 148, 172]. This process naturally occurs in vectors before the exflagellation and gametogenesis [1–3, 7]. The form of gametocytes and their position in erythrocytes changes dramatically during this process, which readily occurs in vitro, including slowly drying blood films. As a result, such material can present distorted parasites and host cells and be unsuitable for species identification, and thus should be eliminated from the taxonomic work. The use of fans is recommended to quicken blood film drying, particularly during fieldwork in humid environments.

Molecular markers (barcodes) were developed and can be used for the detection and identification of approximately 42% of the named species (Table 36). Partial sequences of the mitochondrial cytochrome *b* gene (*cytb*) are easy to obtain using blood samples; they are known for many *Haemoproteus* species, and consequently are convenient and often used for haemoproteid and other haemosporidian parasite species delimitation (barcoding) [38]. However, most detected *Haemoproteus* parasite lineages remain non-characterized at the parasite species levels. Molecular characterization is best developed for the parasites of passeriform birds breeding and migrating in Europe, and it is weakest for the organisms inhabiting tropical bird species, which are more difficult to access for blood sampling [7, 13]. Parasites of most non-passeriform birds remained non-characterized molecularly for the same reason. This is a prominent obstacle in developing generalizations in biodiversity research of *Haemoproteus* and other haemosporidian parasites. It is important to note that some parasite molecular characterizations were based on incorrect species identifications and thus should be treated with caution. Several such cases were reported in Table 36. The development of molecular markers for the diagnosis of agents of haemoproteosis and other haemosporidioses is an important undertaking of current parasitology studies.

Approximately 177 described species of avian *Haemoproteus* currently can be distinguished using morphological characters of their blood stages (Tables 2, 3, 4, 5, 6, 7, 8, 9, 10, 11, 12, 13, 14, 15, 16, 17, 18, 19, 20, 21, 22, 23, 24, 25, 26, 27, 28, 29, 30, 31, 32, 33, 34, 35). The molecular data supported the validity of most comprehensive morphological species descriptions, which were based on a package of distinct morphological characters (Table 36).

**Table 2:** Key to the *Haemoproteus* species of Accipitriformes birds

Step	Features and species	
1 (6)	Fully grown gametocytes, which markedly displace nuclei of infected erythrocytes laterally, often close to the erythrocyte envelope, develop and are common (Fig. 3a, b, d)	
2 (3)	Both broadly halteridial (Fig. 3a, d) and circumnuclear (Fig. 3c, f) fully grown gametocytes occur simultaneously	
	………………………………………………………………………………………………………………….	***H. janovyi*** (Fig. 3a–c) [3, 50]
3 (2)	Fully grown gametocytes predominantly are broadly halteridial (Fig. 3a, d). Circumnuclear fully grown gametocytes (Fig. 3c, f) are absent or occur only occasionally	
4 (5)	Fully grown gametocytes fill the infected erythrocytes up to their poles (Fig. 3d)	
	………………………………………………………………………………………………………………….	***H. elani*** (Fig. 3d) [3, 51, 52]
5 (4)	Fully grown gametocytes predominantly do not fill the infected erythrocytes up to their poles (Fig. 3e)	
	………………………………………………………………………………………………………………….	***H. buteonis*** (Fig. 3e) [3, 53]
6 (1)	Fully grown gametocytes, which markedly displace nuclei of erythrocytes laterally, often close to the erythrocyte envelope (Fig. 3a, b, d), are absent or occur only occasionally. The majority of gametocytes grow around nuclei of the infected erythrocytes (Fig. 3f) and usually do not markedly displace the nuclei laterally (NDR is close to 1). Circumnuclear gametocytes (Fig. 3c, f) predominate among the fully grown gametocytes	
	………………………………………………………………………………………………………………….	***H. nisi*** (Fig. 3f) [3, 53, 54]

**Table 3:** Key to the *Haemoproteus* species of Anseriformes birds

Step	Features and species	
1 (2)	Macrogametocytes often contain one or several large (2.5 μm and bigger) vacuoles (Fig. 4a, b)	
	………………………………………………………………………………………………………………….	***H. macrovacuolatus*** (Fig. 4a, b) [55]
2 (1)	Macrogametocytes do not contain large (2.5 μm and bigger) vacuoles (Fig. 4a, b)	
3 (4)	Fully grown gametocytes predominantly grow around nuclei of infected erythrocytes and do not displace or slightly displace (Fig. 4c, d) the nuclei laterally; they markedly enclose the nuclei with their ends and can completely encircle the nuclei (Fig. 4c). Gametocytes markedly displacing erythrocyte nuclei laterally might occur occasionally during high parasitemia, but never predominate	
	………………………………………………………………………………………………………………….	***H. greineri*** (Fig. 4c, d) [3, 56]
4 (3)	Fully grown gametocytes predominantly markedly displace the nuclei of infected erythrocytes laterally (Fig. 4e, f); they usually slightly enclose the nuclei with their ends but usually do not encircle the nuclei completely. Circumnuclear fully grown gametocytes might occur occasionally during high parasitemia, but never predominate	
	………………………………………………………………………………………………………………….	***H. nettionis*** (Fig. 4e, f) [3, 57, 58]

**Table 4:** Key to the *Haemoproteus* species of Apodiformes birds

Step	Features and species	
1 (2)	Advanced gametocytes grow around nuclei of infected erythrocytes and markedly enclose the nuclei with their ends; fully grown gametocytes can completely encircle the nuclei and occupy all available cytoplasmic space in the erythrocytes (Fig. 5a, b)	
	………………………………………………………………………………………………………………….	***H. archilochus*** (Fig. 5a, b) [3, 59]
2 (1)	Advanced gametocytes grow along the nuclei of infected erythrocytes, but only slightly enclose the nuclei with their ends (Fig. 5c-h). Fully grown gametocytes do not completely encircle the nuclei of infected erythrocytes	
3 (4)	The average number of pigment granules in fully grown gametocytes is greater than 18	
	………………………………………………………………………………………………………………….	***H. witti*** (Fig. 5c, d) [3, 60–63]
4 (3)	The average number of pigment granules in fully grown gametocytes is less than 18	
5 (6)	Growing gametocytes of dumbbell-like shape (Fig. 5e, f) are present	
	………………………………………………………………………………………………………………….	***H. apodus*** (Fig. 5e, f) [3, 64]
6 (5)	Growing gametocytes of dumbbell-like shape (Fig. 5e, f) are absent	
	………………………………………………………………………………………………………………….	***H. trochili*** Fig. (Fig. 5g, h) [3, 60]

**Table 5:** Key to the *Haemoproteus* species of Bucerotiformes birds

Step	Features and species	
1 (2)	Nuclei assume predominantly central or subcentral position in the fully grown macrogametocytes (Fig. 6a); subterminal position of nuclei is not characteristic. Pigment granules tend to aggregate in conspicuous groups (Fig. 6a)	
	………………………………………………………………………………………………………………….	***H. upupae*** (Fig. 6a) [3, 51] ^a^
2 (1)	Nuclei assume predominantly subterminal position in the fully grown macrogametocytes (Fig. 6b); central or subcentral position of nuclei is not characteristic. Pigment granules are randomly scattered (Fig. 6b); they do not tend to aggregate in conspicuous groups	
	………………………………………………………………………………………………………………….	***H. bucerotis*** (Fig. 6b) [3, 65]

^a^Redescription of *H. upupae* is needed. Information about this parasite is scanty

**Table 6:** Key to the *Haemoproteus* species of Caprimulgiformes birds

Step	Features and species	
1	One species has been described. The parasite with pleomorphic gametocytes (Fig. 7a–c). Fully grown gametocytes usually slightly enclose the nuclei of infected erythrocytes with their ends and markedly displace the nuclei laterally, but do not encircle them completely (Fig. 7b). However, the gametocytes sometimes also completely encircle the nuclei of erythrocytes and occupy all available cytoplasmic space in the host cells (Fig. 7c). Advanced growing gametocytes often do not adhere to nuclei of erythrocytes (Fig. 7a). The average number of pigment granules is about 20 in fully grown gametocytes	
	………………………………………………………………………………………………………………….	***H. caprimulgi*** (Fig. 7a–c) [3, 66]

**Table 7:** Key to the *Haemoproteus* species of Cariamiformes birds

Step	Features and species	
1	One species has been described. Advanced growing gametocytes are closely appressed to the envelop of erythrocytes, but often do not touch nuclei of the erythrocytes (Fig. 8b). Form of fully grown gametocytes is close to circumnuclear (Fig. 8a). Fully grown gametocytes cause rounding of the erythrocyte nuclei, which become roundish or even circular in shape (Fig. 8a, b). The average number of pigment granules in fully grown macrogametocytes is greater than in microgametocytes	
	………………………………………………………………………………………………………………….	***H. pulcher*** (Fig. 8a, b) [67]

**Table 8:** Key to the *Haemoproteus* species of Cathartiformes birds

Step	Features and species	
1	One species has been described. Gametocytes are thick, halteridial, with entire margins; they displace host cell nuclei laterally. Macrogametocyte nucleus is close to central position. Growing gametocytes usually do not adhere to erythrocyte nuclei. The number of pigment granules is close to 25 in fully grown gametocytes; the pigment granules are of medium size, scattered throughout the cytoplasm (Fig. 9a, b)	
	………………………………………………………………………………………………………………….	***H. catharti*** (Fig. 9a, b) [68, 69]

**Table 9:** Key to the *Haemoproteus* species of Charadriiformes

Step	Features and species	
1 (2)	Fully grown gametocytes, which rotate erythrocyte nuclei 45° to 90° to the normal axis (Fig. 10a–c), are common	
	………………………………………………………………………………………………………………….	***H. rotator*** (Fig. 10a–c) [3, 70]
2 (1)	Fully grown gametocytes, which rotate erythrocyte nuclei 45° to 90° to the normal axis (Fig. 10a–c), are absent or appear only incidentally	
3 (9)	Fully grown gametocytes, which completely encircle the nuclei of erythrocytes, are present (Fig. 10d, e, h). Close to circumnuclear (Fig. 10f, g, i) and completely circumnuclear (Fig. 10d, e, h) forms are common at final stage of gametocyte growth	
4 (12)	Fully grown circumnuclear macrogametocytes and microgametocytes are closely appressed to the nuclei of infected erythrocytes and occupy all available cytoplasmic space in the erythrocytes (Fig. 10d, h)	
5 (6)	Nucleoli are readily visible in nuclei of advanced and fully grown macrogametocytes (Fig. 10d)	
	………………………………………………………………………………………………………………….	***H. scolopaci*** (Fig. 10d, e) [3, 71]
6 (5)	Nucleoli (Fig. 10d) are invisible in nuclei advanced and fully grown macrogametocytes	
7 (8)	Fully grown gametocytes contain predominantly roundish or slightly oval, of approximately uniform size and shape pigment granules (Fig. 10f, g). Elongate rod-like (thin) pigment granules (Fig. 10h, i) usually are absent	
	………………………………………………………………………………………………………………….	***H. jenniae*** (Fig. 10f, g) [72]
8 (7)	Fully grown gametocytes contain variable in size and shape pigment granules; roundish, oval and elongate rod-like (thin) pigment granules develop, but oval and rod-like pigment granules readily predominate (Fig. 10h, i)	
	………………………………………………………………………………………………………………….	***H. larae*** (Fig. 10h, i) [3, 73, 74]
9 (3)	Fully grown gametocytes, which completely encircle the nuclei of erythrocytes (Fig. 10d, e, h), are absent. Advanced and fully grown gametocytes are halteridial (Fig. 10j, k)	
10 (11)	Fully grown gametocytes contain large (1.0 to 1.5 µm) pigment granules (Fig. 10 j)	
	………………………………………………………………………………………………………………….	***H. abdusalomovi*** (Fig. 10j) [3, 75]
11 (10)	Fully grown gametocytes do not contain large (1.0 to 1.5 µm) pigment granules	
	………………………………………………………………………………………………………………….	***H. burhini*** (Fig. 10k) [3, 65]
12 (4)	Fully grown circumnuclear macrogametocytes are not closely appressed to the nuclei of infected erythrocytes and do not occupy all available cytoplasmic space in the erythrocytes (Fig. 10m, o, p). More or less evident unfilled space is present between fully grown circumnuclear macrogametocytes and nuclei of infected erythrocytes (Fig. 10m, o, p). Shape of fully grown macrogametocytes is markedly irregular (wriggled) (Fig. 10m, o, p)	
13 (14)	Cytoplasm of advanced and fully grown gametocytes is markedly vacuolated, with several large (bigger than 1 µm) vacuoles often present (Fig. 10l, n). Fully grown microgametocytes, which adhere to the nuclei of infected erythrocytes (Fig. 10n), are common	
	………………………………………………………………………………………………………………….	***H. skuae*** (Fig. 10l–n) [76]
14 (13)	Cytoplasm of advanced and fully grown gametocytes is not markedly vacuolated; large (bigger than 1 µm in diameter) vacuoles (Fig. 10l, n) usually are absent, but a few small vacuoles might occur. Fully grown gametocytes, which adhere to the nuclei of infected erythrocytes (Fig. 10n), are absent or occur only occasionally	
	………………………………………………………………………………………………………………….	***H. contortus*** (Fig. 10o, p) [3, 70]

**Table 10:** Key to the *Haemoproteus* species of Ciconiiformes birds

Step	Features and species	
1 (2)	Fully grown gametocytes are broadly halteridial (Fig. 11a). Nuclei of fully grown macrogametocytes are median in position, often ribbon-like in form, closely appressed to the erythrocyte nuclei (Fig. 11a). One or several circular large (about 1 µm and bigger) vacuoles are often present in the macrogametocyte cytoplasm (Fig. 11a)	
	………………………………………………………………………………………………………………….	***H. crumenium*** (Fig. 11a) [3, 77]
2 (1)	Fully grown gametocytes are halteridial (Fig. 11b). Nuclei of fully grown macrogametocytes are median or submedian in position, markedly variable in form and position, usually do not closely appressed to the erythrocyte nuclei (Fig. 11b). Circular conspicuous (about 1 µm and bigger) vacuoles (Fig. 11a) usually are absent from the macrogametocyte cytoplasm, but small vacuoles (less than 0.5 µm) might be present	
	………………………………………………………………………………………………………………….	***H. ciconiae*** (Fig. 11b) [78]

**Table 11:** Key to the *Haemoproteus* species of Coliiformes birds

Step	Features and species	
1	One species has been described. Advanced gametocytes are wavy (undulated) or ameboid in outline (Fig. 12a); they grow around the nuclei of infected erythrocytes and markedly enclose them with their ends, but do not displace or only slightly displace the nuclei laterally (Fig. 12b). Growing gametocytes are usually appressed to the envelope of erythrocytes but do not touch the nuclei of the erythrocytes along their entire margin (Fig. 12a, b). Circumnuclear gametocytes occur	
	………………………………………………………………………………………………………………….	***H. undulatus*** (Fig. 12a, b) [3, 79]

**Table 12:** Key to the *Haemoproteus* species of Columbiformes birds ^a^

Step	Features and species	
1 (12)	Fully grown gametocytes are predominantly halteridial in form (Fig. 13a–i); they outwardly are not similar to gametocytes of *Leucocytozoon* sp*.*	
2 (9)	Volutin is prominent and readily visible in fully grown gametocytes (Fig. 13a–f) ^b^	
3 (8)	Volutin is arranged as discrete granules (volutin granules) (Fig. 13a–e), but does not overfill more or less evenly the entire gametocyte cytoplasm	
4 (5)	Large (1 µm and bigger) discrete volutin granules are present in fully grown microgametocytes (Fig. 13b). Pigment granules (hemozoin) are present inside volutin granules; this is particularly well visible in microgametocytes (Fig. 13b)	
	………………………………………………………………………………………………………………….	***H. columbae***^c^ (Fig. 13a, b) [3, 45, 62, 80–83]
5 (4)	Large (1 µm and bigger) volutin granules (Fig. 13b) are absent from fully grown microgametocytes. Pigment granules (hemozoin) are absent from volutin granules (Fig. 13c–e)	
6 (7)	Fully grown macrogametocytes and microgametocytes markedly displace nuclei of infected erythrocytes laterally (Fig. 13c, d). NDR is less than 0.5	
	………………………………………………………………………………………………………………….	***H. turtur***^d^ (Fig. 13c, d) [3, 45, 82–85]
7 (6)	Fully grown macrogametocytes and microgametocytes do not displace or only slightly displace nuclei of infected erythrocytes laterally (Fig. 13e). NDR is greater than 0.5	
	………………………………………………………………………………………………………………….	***H. palumbis***^c^ (Fig. 13e) [3, 82, 83, 86]
8 (3)	Volutin (Fig. 13a-e) is not arranged as discrete granules (volutin granules); it is arranged as prominent irregular clamps, which often overlap and usually overfill more or less evenly the entire cytoplasm in fully grown gametocytes (Fig. 13f)	
	………………………………………………………………………………………………………………….	***H. multivolutinus***^c^ (Fig. 13f) [82]
9 (2)	Volutin is absent from fully grown gametocytes or difficult to visualize (Fig. 13g, i) ^b^	
10 (11)	The average number of pigment granules in fully grown macrogametocytes and microgametocytes is greater than 30. The earlies young gametocytes look like elongate slender bodies (Fig. 13h)	
	………………………………………………………………………………………………………………….	***H. multipigmentatus***^c^ (Fig. 13g, h) [45, 82, 83]
11 (10)	The average number of pigment granules in fully grown macrogametocytes and microgametocytes is less than 30. The earlies young gametocytes look like broadly ovoid bodies (Fig. 13j)	
	………………………………………………………………………………………………………………….	***H. paramultipigmentatus***^c^ (Fig. 13i, j) [82]
12 (1)	Fully grown gametocytes are highly pleomorphic; many of them are outwardly similar to gametocytes of *Leucocytozoon* (Fig. 13k, l); they cause marked deformation of the infected erythrocytes (Fig. 13k, l). Pigment granules are few (less than 10 on average), tiny (often dust-like in appearance, see Fig. 13l)	
	………………………………………………………………………………………………………………….	***H. sacharovi***^d^ (Fig. 13k, l) [3, 45, 80, 82, 83, 87]

^a^Species of subgenera *Haemoproteus* and *Parahaemoproteus* parasitize birds of order Columbiformes. Parasites of these two subgenera cannot be distinguished based on morphological characters of their gametocytes. To facilitate identification of all species parasitizing columbiform birds, the parasites of both subgenera were given in this key

^b^Visualisation and evaluation of volutin might be difficult in poorly stained blood films or fading preparations. Identification of parasite species should be avoided in poorly stained blood film preparations

^c^Species of subgenus *Haemoproteus*

^d^Species of subgenus *Parahaemoproteus*

**Table 13:** Key to the *Haemoproteus* species of Coraciiformes birds ^a^

Step	Features and species	
1 (6)	Fully grown gametocytes, which displace the nuclei of infected erythrocytes towards one pole of the erythrocytes (Fig. 14a, e) are present; some fully grown gametocytes can enucleate the erythrocytes (Fig. 14b–d)	
2 (5)	The average number of pigment granules in gametocytes is greater than 11. Macrogametocytes usually do not possess a conspicuous large (bigger than 1 µm in diameter) vacuole (Fig. 14e). Microgametocytes with a highly ameboid outline (Fig. 14f) are not characteristic	
3 (4)	Erythrocytes with fully grown gametocytes are significantly atrophied in width in comparison to uninfected erythrocytes	
	………………………………………………………………………………………………………………….	***H. lairdi*** (Fig. 14a, b) [3, 88]
4 (3)	Erythrocytes with fully grown gametocytes are not significantly atrophied in width (Fig. 14c) in comparison to uninfected erythrocytes	
	………………………………………………………………………………………………………………….	***H. enucleator*** (Fig. 14c, d) [3, 89]
5 (2)	The average number of pigment granules in gametocytes is less than 11. Macrogametocytes frequently possess one conspicuous large (greater than 0.5 µm in diameter) vacuole (Fig. 14e). Microgametocytes with a highly ameboid outline (Fig. 14f) are common	
	………………………………………………………………………………………………………………….	***H. gavrilovi*** (Fig. 14e, f) [3, 90, 91]
6 (1)	Fully grown gametocytes, which displace the nuclei of infected erythrocytes toward one pole of the erythrocytes (Fig. 14a, e) are absent; fully grown gametocytes do not enucleate the erythrocytes	
7 (10)	Fully grown gametocytes markedly enclose the nuclei of infected erythrocytes with their ends (Fig. 14g–k) and finally can completely encircle the nuclei (Fig. 14g, i). Circumnuclear gametocytes develop (Fig. 14g, h)	
8 (9)	Circumnuclear fully grown gametocytes are common (Fig. 14i). Advanced growing gametocytes usually do not adhere to nuclei of infected erythrocytes (Fig. 14h). The average number of pigment granules in gametocytes is greater than 20	
	………………………………………………………………………………………………………………….	***H. fuscae*** (Fig. 14g–i) [3, 92, 93]
9 (8)	Fully grown gametocytes markedly enclose the nuclei of infected erythrocytes with their ends (Fig. 14j, k) and can completely encircle the nuclei of erythrocytes, but the circumnuclear fully grown gametocytes are uncommon and might appear only at final stages of gametocyte maturation. Advanced growing gametocytes are closely appressed to nuclei of the infected erythrocytes (Fig. 14j). The average number of pigment granules in gametocytes is less than 20	
	………………………………………………………………………………………………………………….	***H. coraciae*** (Fig. 14j, k) [3, 90, 94]
10 (7)	Fully grown gametocytes only slightly enclose the nuclei of infected erythrocytes with their ends (Fig. 14l–t) and never encircle them completely. Fully grown gametocytes are halteridial or broadly halteridial (Fig. 14l–t). Circumnuclear gametocytes (Fig. 14g, i) do not develop	
11 (16)	Nuclei of microgametocytes are diffuse (non-condensed) (Fig. 14p, r); size of microgametocyte nuclei is greater than in macrogametocytes (compare Fig. 14p, r with Fig. 14q)	
12 (13)	The average number of pigment granules in gametocytes is greater than 15	
	………………………………………………………………………………………………………………….	***H. eurystomae*** (Fig. 14l–n) [3, 95]
13 (12)	The average number of pigment granules in gametocytes is less than 15	
14 (15)	Fully grown gametocytes are broadly halteridial (Fig. 14o, p). The cytoplasm of advanced macrogametocytes often contains conspicuous small (usually less than 1 µm in diameter) circular vacuoles (Fig. 14o). The average width of fully grown gametocytes is greater than 4 µm. The average NDR is 0.5 or less	
	………………………………………………………………………………………………………………….	***H. manwelli*** (Fig. 14o, p) [3, 88, 91]
15 (14)	Fully grown gametocytes are halteridial (Fig. 14q, r). The cytoplasm of advanced macrogametocytes usually do not contains conspicuous small circular vacuoles (Fig. 14o). The average width of fully grown gametocytes is less than 4 µm. The average NDR is greater than 0.5	
	………………………………………………………………………………………………………………….	***H. meropis*** (Fig. 12q, r) [3, 88]
16 (11)	Nuclei of microgametocytes are small (condensed) (Fig. 14t); size of nuclei in macrogametocytes Fig. 14s) and microgametocytes (Fig. 14t) are similar	
	………………………………………………………………………………………………………………….	***H. halcyonis*** (Fig. 14s, t) [3, 92]

^a^*Haemoproteus goodmani* and *H. forresteri* were described from the pitta-like ground-roller *Atelornis pittoides* and the rufous-headed ground-roller *Atelornis crossleyi*, respectively [96]. These birds belong to the Brachypteraciidae, the endemic to Madagascar bird family of Coraciiformes. Descriptions of these two parasite species were based on morphological characters of gametocytes from the pale-stained blood films, which are fading. It is clear from the original description that the fully grown gametocytes of *H. goodmani* are broadly halteridial with variable margins. This parasite is different from *H. forresteri,* whose fully grown gametocytes are circumnuclear. However, further taxonomically important details of gametocyte morphology of both species were scarcely addressed in the original description. Due to limited available information about morphology of gametocytes, *H. goodmani* and *H. forresteri* were not included in the key. Redescription of both parasites from their type hosts is needed

**Table 14:** Key to the *Haemoproteus* species of Cuculiformes birds

Step	Features and species^a^	
1 (2)	The average number of pigment granules in fully grown macrogametocytes is greater than 20	
	………………………………………………………………………………………………………………….	***H. cuculis*** (Fig. 15a) [97]
2 (1)	The average number of pigment granules in fully grown gametocytes is less than 20	
3(4)	Pigment granules in fully grown gametocytes tend to aggregate into compact large masses or loose clumps (Fig. 15c, e). Volutin granules were not reported	
	………………………………………………………………………………………………………………….	***H. centropi*** (Fig. 15b-e) [3, 97, 98]
4 (3)	Pigment granules in fully grown gametocytes are not aggregated into compact large masses or loose clumps (Fig. 15c, e). Volutin granules usually present	
	………………………………………………………………………………………………………………….	***H. clamatori*** (Fig. 15f) [97]

^a^Original descriptions of *H. centropi, H. cuculis* and *H. clamatori* were incomplete, however, these parasites can be distinguished due to differences in number and morphology of pigment granules in fully grown gametocytes. Fully grown gametocytes of these parasites are broadly halteridial and markedly displace nuclei of infected erythrocytes laterally. Details of morphology of growing gametocytes remain insufficiently described. Redescription of these parasites is needed

**Table 15:** Key to the *Haemoproteus* species of Falconiformes birds^a^

Step	Features and species	
1 (2)	Gametocytes grow around the nuclei of infected erythrocytes (Fig. 16a–c). Advanced growing gametocytes are closely appressed to envelope of the infected erythrocytes (Fig. 16a). Growing gametocytes markedly displace the erythrocyte nuclei laterally (Fig. 16a). Outline of growing gametocytes vary from even to amoeboid. Fully grown gametocytes can completely encircle erythrocyte nuclei (Fig. 16b, c) and occupy all available cytoplasmic space in the erythrocytes, but broadly halteridial fully grown gametocytes also are present. Erythrocytes containing fully-grown circumnuclear gametocytes often assume roundish form (Fig. 16c)	
	………………………………………………………………………………………………………………….	***H. tinnunculi*** (Fig. 16a–c)[3, 99, 100]
2 (1)	Gametocytes grow around the nuclei of infected erythrocytes (Fig. 16d–f). Advanced growing gametocytes often do not adhere to envelope of erythrocytes (Fig. 16d). Growing gametocytes do not displace or only slightly displace the erythrocyte nuclei laterally (Fig. 16d, e). Growing gametocytes usually are highly irregular (Fig. 16d) or amoeboid in outline, with readily visible prominent and variable in form outgrowths (Fig. 16e). Fully grown gametocytes nearly completely (Fig. 16f) or even completely encircle the nuclei of infected erythrocytes, they usually do not occupy all available cytoplasmic space in the erythrocytes (Fig. 16f). Infected erythrocytes do not assume roundish form (compare Fig. 16c with Fig. 16f)	
	………………………………………………………………………………………………………………….	***H. brachiatus*** (Fig. 16d–f) [3, 100]

^a^The authors examined 12 single infections of *H. tinnunculi* from Eurasian hobby *Falco subbuteo* and Common kestrel *Falco* *tinnunculus* sampled in Eurasia and came to the conclusion that *Haemoproteus obainae* and *Haemoproteus deharoi* parasites, whose were described [101] in same avian hosts are morphological variants of *H. tinnunculi*. Gametocytes indistinguishable from *H. obainae* were present in each infected bird; they have similar cell structure (morphology of pigment, position of nuclei in macrogametocytes and appearance of the cytoplasm) and represent the final stage of development of *H. tinnunculi* in avian hosts. Description of *H. deharoi* shows rounded gametocytes, which appear within several minutes in most *Haemoproteus* infections as a result of preparation of mature gametocytes to exflagellation after exposure of blood to air [3]. This might occur if blood films were prepared or/and dried slowly after withdrawal of the blood from avian hosts. Both *H. obainae* and *H. deharoi* were considered as synonyms of *H. tinnunculi*

**Table 16:** Key to the *Haemoproteus* species of Galliformes birds

Step	Features and species	
1 (16)	Advanced growing and fully grown macrogametocytes do not contain one large (bigger than 1 µm in diameter) circular vacuole (Fig. 17j–l)	
2 (7)	Gametocytes grow around nuclei of infected erythrocytes; they markedly enclose the nuclei with their ends (Fig. 17a–d) and finally encircle the nuclei completely. Fully grown gametocytes are circumnuclear (Fig. 17a, b, d)	
3 (6)	Fully grown gametocytes usually do not occupy all available cytoplasmic space in infected erythrocytes. A more or less evident unfilled space (a ‘cleft’) usually is present between the fully grown gametocyte and the nucleus of erythrocyte (Fig. 17a–c)	
4 (5)	The average number of pigment granules in macrogametocytes is greater than 15	
	………………………………………………………………………………………………………………….	***H. lophortyx*** (Fig. 17a,b) [3, 102]
5 (4)	The average number of pigment granules in macrogametocytes is less than 15	
	………………………………………………………………………………………………………………….	***H. stableri*** (Fig. 17c) [3, 103]
6 (3)	Fully grown gametocytes occupy all available cytoplasmic space in infected erythrocytes (Fig. 17d). An unfilled space (a ‘cleft’) (Fig. 17a, b) is absent between the fully grown gametocyte and nucleus of the erythrocyte	
	………………………………………………………………………………………………………………….	***H. mansoni*** (probable syn. *H. meleagridis*) (Fig. 17d) [3, 104]
7 (2)	Fully grown gametocytes do not encircle the nuclei of infected erythrocytes completely. Fully grown gametocytes are halteridial (Fig. 17e–i)	
8 (14)	The average number of pigment granules in fully grown macrogametocytes is greater than 15	
9 (15)	The outline of fully grown gametocytes predominantly is even (Fig. 17e–g)	
10 (13)	Growing gametocytes with a highly constricted central portion, causing a ‘dip’ and presenting a dumbbell-like shape (Fig. 2e), are absent	
11 (12)	Fully grown macrogametocytes markedly displace the nuclei of infected erythrocytes laterally (Fig. 17e); the average NDR is less than 0.7	
	………………………………………………………………………………………………………………….	***H. pratasi*** (Fig. 17e) [3, 105]
12 (11)	Fully grown gametocytes do not displace or slightly displace the nuclei of infected erythrocytes laterally (Fig. 17f); the average NDR is greater than 0.7	
	………………………………………………………………………………………………………………….	***H. ammoperdix*** (Fig. 17f) [3]
13 (10)	Growing gametocytes with a highly constricted central portion, causing a ‘dip’ and presenting a dumbbell-like shape (Fig. 2e), are present	
	………………………………………………………………………………………………………………….	***H. megapodius***^a^ [3, 106]
14 (8)	The average number of pigment granules in fully grown macrogametocytes is less than 15	
	………………………………………………………………………………………………………………….	***H. rileyi*** (Fig. 17g) [3, 105]
15 (9)	The outline of fully grown and advanced gametocytes predominantly is highly ameboid (Fig. 17h, i)	
	………………………………………………………………………………………………………………….	***H. cracidarum*** (Fig. 17h, i) [3, 107]
16 (1)	Advanced growing and fully grown macrogametocytes often contain one large (bigger than 1 µm in diameter) circular vacuole (Fig. 17j–l)	
17 (18)	Fully grown gametocytes of roundish or various oval forms (Fig. 17k, l) are absent. Vacuole-like unstained spaces might be present on one or both ends of maturing and fully grown macrogametocytes (Fig. 17j). Gametocytes are overfilled with prominent distinct volutin granules (Fig. 17j)	
	………………………………………………………………………………………………………………….	***H. paraortalidum*** (Fig. 17j) [108]
18 (17)	Fully grown gametocytes of roundish or various oval forms (Fig. 17k, l) are present. Vacuole-like unstained spaces (Fig. 17j) are absent on both ends of growing or fully grown macrogametocytes. Gametocytes contain volutin, which is dispersed (Fig. 17k, l)	
	………………………………………………………………………………………………………………….	***H. ortalidum*** (Fig. 17k, l) [3, 109]

^a^Original *H. megapodius* description is incomplete, and the type material was not available for investigation. Redescription of this pathogen is needed

**Table 17:** Key to the *Haemoproteus* species of Gruiformes birds

Step	Features and species	
1 (6)	Growing dumbbell-shaped gametocytes (Fig. 18f, g) are absent	
2 (3)	Growing and fully grown gametocytes are elongate and slender (Fig. 18a, b); they do not display or only slightly display nuclei of the infected erythrocytes laterally (Fig. 18a, b). The average NDR is close to 1	
	………………………………………………………………………………………………………………….	***H. balearicae*** (Fig. 18a, b) [3, 110]
3 (2)	Growing and fully grown gametocytes are broadly halteridial (Fig. 18c, d); they do not assume slender form (Fig. 18a, b). Fully grown gametocytes markedly display nuclei of the infected erythrocytes laterally (Fig. 18d, e); the average NDR is less than 0.5	
4 (5)	Average number of pigment granules in macrogametocytes is greater than 25	
	………………………………………………………………………………………………………………….	***H. gallinulae*** (Fig. 18c, d) [3, 111, 112]
5 (4)	Average number of pigment granules in macrogametocytes is less than 25	
	………………………………………………………………………………………………………………….	***H. antigonis*** (Fig. 18e) [3, 113]
6 (1)	Growing dumbbell-shaped gametocytes (Fig. 18f, g) are common	
	………………………………………………………………………………………………………………….	***H. porzanae*** (Fig. 18f–h) [3, 112]

**Table 18:** Key to the *Haemoproteus* species of Musophagiformes birds

Step	Features and species	
1 (2)	Fully grown gametocytes are halteridial; they only slightly enclose nuclei of infected erythrocytes with their ends (Fig. 19a, b). Both ends of advanced gametocytes usually are more or less narrowed in comparison to the widths of the gametocytes (Fig. 19a, b). One or both ends of advanced gametocytes often are pointed (Fig. 19a, b)	
	………………………………………………………………………………………………………………….	***H. montezi*** (Fig. 19a, b) [3, 114]
2 (1)	Fully grown gametocytes are halteridial; they only slightly enclose nuclei of infected erythrocytes with their ends (Fig. 19c). Both ends of advanced gametocytes usually are not narrowed in comparison to the widths of the gametocytes (Fig. 19c). Both ends of advanced gametocytes usually are approximately similarly rounded (Fig. 19c)	
	………………………………………………………………………………………………………………….	***H. minchini*** (Fig. 19c) [115]

**Table 19:** Key to the *Haemoproteus* species of Otidiformes birds

Step	Features and species	
1	One species has been described. Growing gametocytes are closely appressed to the erythrocyte envelope but usually do not touch the erythrocyte nuclei, and as a result, a more or less evident unfilled space (a ‘cleft’) is present between the gametocytes and the erythrocyte nuclei (Fig. 20a). Fully grown gametocytes are closely appressed both to the nuclei and envelope of erythrocytes; they completely encircle the nuclei and occupy all available cytoplasmic space in the erythrocytes (Fig. 20b). The average number of pigment granules is about 17 in macrogametocytes	
	………………………………………………………………………………………………………………….	***H. telfordi*** (Fig. 20a, b) [3, 113]

**Table 20:** Key to the *Haemoproteus* species of Pelecaniformes birds

Step	Features and species	
1 (2)	Fully grown gametocytes occupy all or nearly all cytoplasmic space in infected erythrocytes, including poles of the erythrocytes (Fig. 21a, b); they vary from broadly halteridial (Fig. 21a, c) to circumnuclear (Fig. 21b) in form (the former form predominates). The average number of pigment granules in macrogametocytes is greater than 25, and it is approximately half as many in microgametocytes (compare Fig. 21a, b with Fig. 21c)	
	………………………………………………………………………………………………………………….	***H. plataleae*** (Fig. 21a–c) [3, 116]
2 (1)	Fully grown gametocytes usually do not occupy all or nearly all cytoplasmic space in infected erythrocytes, including poles of erythrocytes (Fig. 21d, f, i); they are thin halteridial in form (Fig. 21d–f, i). The average number of pigment granules in macrogametocytes is less than 25, and it is approximately the same in macrogametocytes and microgametocytes	
3 (4)	Markedly ameboid forms predominate among young gametocytes (Fig. 21e). Advanced growing and fully grown gametocytes are predominantly markedly irregular in outline (Fig. 21d, f)	
	………………………………………………………………………………………………………………….	***H. pelouroi*** (Fig. 21d–f) [3, 116, 117]
4 (3)	Ameboid forms do not predominate among young gametocytes; outline of growing gametocytes is predominantly even (Fig. 21g–i). Advanced growing (Fig. 21g, h) and fully grown gametocytes (Fig. 21i) are predominantly even in outline, but gametocytes with slightly wavy margins might occur occasionally	
	………………………………………………………………………………………………………………….	***H. herodiadis*** (Fig. 21g–i) [3, 118]

**Table 21:** Key to the *Haemoproteus* species of Piciformes birds

Step	Features and species	
1 (8)	Fully grown gametocytes do not encircle completely the nuclei of infected erythrocytes. Circumnuclear gametocytes (Fig. 22c, d) are absent	
2 (11)	Fully grown gametocytes do not enucleate the infected erythrocytes	
3 (16)	Clearly dumbbell-like or bilobed in shape (Fig. 22l, m) fully grown gametocytes are absent	
4 (17)	The average number of pigment granules in fully grown macrogametocytes and microgametocytes is similar	
5 (18)	Macrogametocyte nuclei are predominantly in median position (Fig. 22a, b). Fully grown gametocytes do not contain large compact volutin granules (Fig. 22p)	
6 (7)	Advanced growing gametocytes and fully grown gametocytes are closely appressed to nuclei of infected erythrocytes (Fig. 22a)	
	………………………………………………………………………………………………………………….	***H. xantholaemae*** (Fig. 22a) [3, 119]
7 (6)	Advanced growing gametocytes and fully grown gametocytes, which do not touch nuclei of infected erythrocytes (Fig. 22b) are common	
	………………………………………………………………………………………………………………….	***H. cornuata*** (Fig. 22b) [3, 119]
8 (1)	Fully grown gametocytes encircle completely the nuclei of infected erythrocytes (Fig. 22c, d). Growing gametocytes readily tend to grow around nuclei of the erythrocytes	
9 (10)	Advanced growing and fully grown gametocytes contain numerous prominent volutin granules and clearly distinct volutin clumps (Fig. 22c), which mask pigment granules^a^	
	………………………………………………………………………………………………………………….	***H. velans*** (Fig. 22c) [3, 87, 120, 121]
10 (9)	Advanced growing and fully grown gametocytes do not contain numerous prominent volutin granules and their clearly distinct clumps (Fig. 22c), which mask pigment granules^a^	
	……………………………………………….	***H. homovelans*** (Fig. 22d) [121]
11 (2)	Fully grown gametocytes enucleate the infected erythrocytes at final stage of development (Fig. 22e, g, h). Growing advanced gametocytes markedly displace nuclei of infected erythrocytes, often to erythrocyte poles (Fig. 22f, i, k)^b^	
12 (15)	The average number of pigment granules in fully grown gametocytes is less than 25	
13 (14)	Infected erythrocytes are hypertrophied (on average approximately 10%) in length in comparison to uninfected ones	
	………………………………………………………………………………………………………………….	***H. bennetti*** (Fig. 22e–g) [3, 122]
14 (13)	Infected erythrocytes do not change significantly on average in length in comparison to uninfected ones	
	………………………………………………………………………………………………………………….	***H. bucconis*** (Fig. 22h) [3, 64]^c^
15 (12)	The average number of pigment granules in fully grown gametocytes is greater than 25	
	………………………………………………………………………………………………………………….	***H. thereicerycis*** (Fig. 22i–k) [3, 119]
16 (3)	Clearly dumbbell-like or bilobed in shape (Fig. 22l, m) fully grown gametocytes are common	
	………………………………………………………………………………………………………………….	***H. bilobata*** (Fig. 22l, m) [3, 119]
17 (4)	The average number of pigment granules in fully grown macrogametocytes is approximately half as many as in fully grown microgametocytes (compare Fig. 22n and o)	
	………………………………………………………………………………………………………………….	***H. indicator*** (Fig. 22n, o) [3, 64]
18 (5)	Macrogametocyte nuclei are predominantly of sub-terminal position (Fig. 22p). Fully grown gametocytes contain large compact volutin granules	
	………………………………………………………………………………………………………………….	***H. borgesi*** (Fig. 22p) [3, 122]

^a^Volutin is always readily visible, but might be present markedly unequally in different infections

^b^Availability of gametocytes in enucleated erythrocytes is a function of the parasitemia stage. Such gametocytes might be rare or even absent in blood films, in which growing gametocytes predominate

^c^Redescription of *H. bucconis* is needed. Type material of this species is fading and many morphological characters are indistinguishable. This parasite is similar to *H. bennetti*. These two species can be distinguished mainly due to little differences in influence of fully grown gametocytes on the length of infected erythrocytes. The taxonomic value of this character needs clarification

**Table 22:** Key to the *Haemoproteus* species of Psittaciformes birds

Step	Features and species	
1 (4)	Fully grown gametocytes are circumnuclear; they markedly enclose the nuclei of infected erythrocytes with their ends and finally can completely encircle the nuclei (Fig. 23a–c). The average number of pigment granules in gametocytes is greater than 15	
2 (3)	Elongate (rod-like) pigment granules present and often predominate in advanced and fully grown gametocytes (Fig. 23a)	
	…………………………………………………………………………………………………….	***H. handai*** (Fig. 23a, b) [3, 123, 124]
3 (2)	Elongate (rod-like) pigment granules are rare, they do not predominate in advanced and fully grown gametocytes. Roundish or slightly oval pigment granules predominate (Fig. 23c)	
	………………………………………………………………………….	***H. homohandai*** (Fig. 23c) [125]
4 (1)	Fully grown gametocytes are halteridial, they do not encircle the nucleus of infected erythrocytes completely (Fig. 23d, e). The average number of pigment granules in gametocytes is less than 15	
	………………………………………………………………………….	***H. psittaci*** (Fig. 23d, e) [3, 126]

**Table 23:** Key to the *Haemoproteus* species of Pterocliformes birds

Step	Features and species^a^	
1 (2)	Nuclei of macrogametocytes are median in position (Fig. 24a)	
	………………………………………………………………………………………………………………….	***H. krylovi*** (Fig. 24a) [3]
2 (1)	Nuclei of macrogametocytes are subterminal in position^b^	
	………………………………………………………………………………………………………………….	***H. pteroclis* (**Fig. 24b) [3, 127]

^a^The original descriptions of the haemoproteids parasitizing Pterocliformes birds were fragmentary and incomplete. Redescription of *H. krylovi* and *H. pteroclis* is needed

^b^Subterminal position of nuclei in macrogametocytes of *H. pteroclis* was mentioned in the original parasite description, but it was not illustrated [127]

**Table 24:** Key to the *Haemoproteus* species of Strigiformes birds^a^

Step	Features and species	
1 (2)	Volutin is conspicuous in fully grown gametocytes; it is arranged as compact roundish or circular granules, which tend to gather close to gametocyte ends (Fig. 25a-c). Fully grown gametocytes are halteridial (Fig. 25b) or close to circumnuclear, occasionally circumnuclear (Fig. 25c); halteridial forms (Fig. 25b) usually common	
	………………………………………………………………………………………………………………….	***H. syrnii*** (Fig. 25a–c) [3, 128–130]^b^
2 (1)	Volutin is present, but is not conspicuous in fully grown gametocytes (Fig. 25d, e); it is dispersed more or less evenly (Fig. 25d, e), but not arranged as compact roundish or circular granules, which tend to gather close to gametocytes ends (Fig. 25a–c). Fully grown gametocytes are predominantly close to circumnuclear (Fig. 25d), often circumnuclear (Fig. 25e); halteridial forms (Fig. 25b) are rare	
	………………………………………………………………………………………………………………….	***H. noctuae*** (Fig. 25d, e) [3, 130]

^a^*Haemoproteus ilanpapernai* [129] was considered as a *species inquirenda*. This haemoproteid probably belongs to *H. syrnii* or the same group of closely related species or subspecies. The original *H. ilanpapernai* description shows markedly rounded gametocytes, which likely represent changes due to preparation to the exflagellation in slowly drying blood films; this is a common phenomenon in avian haemoproteids preparations, which dry slowly in humid environments. Confirmation of existence of this organism is needed by examination of fresh material from type vertebrate host

^b^Well-stained blood films are needed for the evaluation of morphology of volutin in gametocytes

**Table 25:** Key to the *Haemoproteus* species of Suliformes birds^a^

Step	Features and species	
1 (2)	The average number of pigment granules is greater than 40 in fully grown macrogametocytes (Fig. 26a). The average number of pigment granules in fully grown macrogametocytes is at least twice that in fully grown microgametocytes (compare Fig. 26a and b)	
	………………………………………………………………………………………………………………….	***H. iwa***^b^ (Fig. 26a, b) [3, 131, 132]
2 (1)	The average number of pigment granules is less than 40 in fully grown macrogametocytes (Fig. 26c). The average number of pigment granules in fully grown macrogametocytes is not twice that in fully grown microgametocytes (Fig. 26c and d)	
	………………………………………………………………………………………………………………….	***H. valkiunasi***^c^ (Fig. 26c, d) [46]

^a^Based on phylogenetic analysis [46], the species of subgenera *Haemoproteus* and *Parahaemoproteus* parasitize birds of order Suliformes. Parasites of these two subgenera cannot be distinguished based on morphological characters of their gametocytes. To facilitate identification of all species parasitizing Suliformes birds, the parasites of both subgenera were given in this key

^b^Species of subgenus *Haemoproteus*

^c^Species of subgenus *Parahaemoproteus*

**Table 26:** Key to the *Haemoproteus* species of Passeriformes birds (suborder Tyranni) of the families Eurylaimidae, Furnariidae, Pittidae, Thamnophilidae, Tyrannidae^a^

Step	Features and species	
1 (4)	Gametocytes tend to grow around nuclei of infected erythrocytes and advanced forms markedly enclose the nuclei with their ends. Fully grown gametocytes finally completely encircle the nuclei of infected erythrocytes (Fig. 27a–c) and can occupy all available cytoplasmic space in the erythrocytes (Fig. 27b)	
2 (3)	Close to circumnuclear and circumnuclear gametocytes readily predominate among advanced and fully grown both macrogametocytes and microgametocytes (Fig. 27a, b). Halteridial gametocytes, which markedly displace nuclei of infected erythrocytes are absent or appear only occasionally	
	………………………………………………………………………………………………………………….	***H. circumnuclearis*** (Fig. 27a, b) [3, 133]
3 (2)	Circumnuclear gametocytes readily predominate among advanced and fully frown macrogametocytes (Fig. 27c), but do not predominate among advanced and fully frown microgametocytes, which often assume broadly halteridial forms and markedly displace host cell nuclei (Fig. 27d)	
	………………………………………………………………………………………………………………….	***H. pittae*** (Fig. 27c, d) [3, 134]
4 (1)	Gametocytes do not tend to grow around nuclei of infected erythrocytes, they do not markedly enclose the nuclei with their ends (Fig. 27e–i). Fully grown gametocytes are halteridial; they do not completely encircle nuclei of infected erythrocytes and never occupy all available cytoplasmic space in the erythrocytes (Fig. 27e, g–i)	
5 (6)	Advanced growing gametocytes, which length is bigger than the length of erythrocyte nuclei, are closely appressed to the nuclei and envelope of infected erythrocytes (Fig. 27e)	
	………………………………………………………………………………………………………………….	***H. tyranni*** (Fig. 27e) [3, 133]
6 (5)	Advanced growing gametocytes, which length is bigger than the length of erythrocyte nuclei, predominantly do not touch the envelope of infected erythrocytes along their entire margins (Fig. 27f)	
7 (8)	The average number of pigment granules in gametocytes is close to 10 (Fig. 27g). Nuclei assume predominantly subterminal position in macrogametocytes (Fig. 27f, g). Attenuated (snake-like) advanced gametocytes develop (Fig. 27f)	
	………………………………………………………………………………………………………………….	***H. furnarius*** (Fig. 27f, g) [3, 135]
8 (7)	The average number of pigment granules in gametocytes is close to 15 or greater (Fig. 27h, i). Macrogametocyte nuclei assume predominantly median position (Fig. 27i) or their position is variable (median, submedian and subterminal) in different cells (Fig. 27h, i). Attenuated (snake-like) advanced gametocytes (Fig. 27f) do not develop	
9 (10)	The average number of pigment granules in fully grown gametocytes is close to 15; medium size pigment granules (0.5–1 µm) predominate, but large (greater than 1 µm) pigment granules also might occur (Fig. 27h)	
	………………………………………………………………………………………………………………….	***H. formicarius*** (Fig. 27h) [3, 135]
10 (9)	The average number of pigment granules in fully grown gametocytes is close to 20; small (usually less than 0.5 µm) pigment granules predominate, and large (greater than 1 µm) pigment granules do not occur (Fig. 27i)	
	………………………………………………………………………………………………………………….	***H. eurylaimus*** (Fig. 27i) [3, 134]

^a^*Haemoproteus souzalopesi* was described in species of the Tyrannidae [133]. This parasite has roundish gametocytes, which are similar to gametocytes of *Plasmodium (Haemamoeba)* species [3]. Examination of the hapantotype of this species (Queensland Museum accession number G440399, IRCAH accession number 83024) showed presence of numerous roundish gametocytes, but also numerous developing meronts of *P. (Haemamoeba)* species, suggesting that co-occurring gametocytes also belong to *Plasmodium* parasite. Species *souzalopesi* likely belong to genus *Plasmodium*. Further studies are needed to clarify this taxonomical uncertainty. *Haemoproteus souzalopesi* was considered as *species inquirenda* and not included in the key for identification

**Table 27:** Key to the *Haemoproteus* species of Passeriformes birds (suborder Passeri) of the families Meliphagidae, Oriolidae, Pachycephalidae, Vireonidae

Step	Features and species	
1 (5)	Growing advanced gametocytes (size greater than length of erythrocyte nuclei), which do not touch the envelope of infected erythrocytes along their entire margin (Fig. 28e, f), are absent	
2 (6)	Nuclei predominantly assume subterminal or submedian position in fully grown macrogametocytes (Fig. 28a, b)	
3 (4)	Fully grown macrogametocytes and microgametocytes are halteridial (Fig. 28b). Pigment granules are markedly variable in size and form in advanced gametocytes (Fig. 28a). Small (less than 0.5 μm) and medium (0.5 to 1.0 μm) size pigment granules occur approximately equally in fully grown gametocytes (Fig. 28a, b)	
	………………………………………………………………………………………………………………….	***H. vireonis*** (Fig. 28a, b) [3, 62, 135, 136]
4 (3)	Fully grown macrogametocytes are halteridial (Fig. 28d), but microgametocytes are more pleomorphic (halteridial, close to circumnuclear and occasionally even circumnuclear forms occur). Pigment granules are usually more or less uniform in size and form in advanced gametocytes (Fig. 28c, d). Pigment granules in fully grown gametocytes are usually of medium size (0.5 to 1.0 μm). Small (less than 0.5 μm) pigment granules are not present or are uncommon in fully grown gametocytes (Fig. 28d)	
	………………………………………………………………………………………………………………….	***H. ptilotis*** (Fig. 28c, d) [3, 137, 138]
5 (1)	Growing advanced gametocytes (size greater than length of erythrocyte nuclei), which do not touch the envelope of infected erythrocytes along their entire margin (Fig. 28e, f), are common	
	………………………………………………………………………………………………………………….	***H. pachycephalus*** (Fig. 28e, f) [3, 139]
6 (2)	Nuclei predominantly assume terminal or close to the terminal position in fully grown macrogametocytes (Fig. 28g, h)	
	………………………………………………………………………………………………………………….	***H. orioli*** (Fig. 28g, h) [3, 140]

**Table 28:** Key to the *Haemoproteus* species of Passeriformes birds (suborder Passeri), of the families Aegithinidae, Artamidae, Malaconotidae, Vangidae

Step	Features and species	
1 (6)	Growing and fully grown macrogametocytes do not contain large (greater that 1.5 µm in diameter) vacuoles (Fig. 29d, e)	
2 (7)	Fully grown gametocytes are halteridial; they reach poles of infected erythrocytes and occupy the poles completely (Fig. 29a)	
3 (8)	Advanced growing gametocytes are closely appressed to nuclei of infected erythrocytes and often do not touch envelope of the erythrocytes along their entire margin (Fig. 29b, c)	
4 (5)	The average number of pigment granules in fully grown gametocytes is greater than 12	
	………………………………………………………………………………………………………………….	***H. aegithinae*** (Fig. 29a–c) [3, 141]
5 (4)	The average number of pigment granules in fully grown gametocytes is less than 12	
	………………………………………………………………………………………………………………….	***H. vangii***^a^ [142]
6 (1)	Advanced growing and fully grown macrogametocytes often contain one large (greater that 1.5 µm in diameter) vacuole, which is of sub-terminal position in gametocytes and adheres to the erythrocyte envelope; the vacuole usually locates at gametocyte end, which is opposite in location to the gametocyte nucleus (Fig. 29d, e). Occasionally, up to three or even more small vacuoles can be present	
	………………………………………………………………………………………………………………….	***H. bukaka*** (Fig. 29d, e) [143]
7 (2)	Fully grown gametocytes are microhalteridial; they usually do not reach poles of infected erythrocytes	
	………………………………………………………………………………………………………………….	***H. madagascariensis***^a^ [142]
8 (3)	Advanced growing gametocytes often do not touch nuclei of infected erythrocytes along their entire margin, but are closely appressed to erythrocyte envelope (Fig. 29f)	
	………………………………………………………………………………………………………………….	***H. cublae*** (Fig. 29f) [3, 144]

^a^Images of *H. vangii* and *H. madagascariensis* are not available. Type preparations of these parasites are markedly fading, and the provided diagnostic characters are based on the original description [142]. Redescription of these species is needed for better knowledge about other delicate diagnostic characters

**Table 29:** Key to the *Haemoproteus* species of Passeriformes birds (suborder Passeri) of the families Corvidae, Dicruridae, Laniidae, Monarchidae

Step	Features and species	
1 (7)	The average number of pigment granules in fully grown gametocytes is less than 20. Circumnuclear gametocytes (Fig. 30e) are absent or occur only occasionally among fully grown macrogametocytes	
2 (8)	The nuclei of macrogametocytes are in subterminal (Fig. 30a, b) or close to terminal (Fig. 30j) positions and, as a rule, they are not closely appressed to the nuclei of infected erythrocytes (Fig. 30g)	
3 (9)	Dumbbell-shaped growing gametocytes (Fig. 30b, i) are common	
4 (10)	Dumbbell-shaped growing gametocytes, which do not touch the envelope of erythrocytes along their entire margin (Fig. 30i), are absent. Fully grown gametocytes fill the infected erythrocytes up to their poles (Fig. 30a, d)	
5 (6)	The cytoplasm is markedly vacuolated in growing and fully grown microgametocytes, with several distinct large (greater than 1 µm in diameter) vacuoles often present (Fig. 30c)	
	………………………………………………………………………………………………………………….	***H. homopicae*** (Fig. 30a–c) [100]
6 (5)	The cytoplasm is not vacuolated or only slightly vacuolated in growing and fully grown microgametocytes (Fig. 30d); it does not contain distinct large (greater than 1 µm in diameter) vacuoles (Fig. 30c)	
	………………………………………………………………………………………………………………….	***H. picae*** (Fig. 30d) [3, 87, 145]
7 (1)	The average number of pigment granules in fully grown gametocytes is 20 or greater. Circumnuclear gametocytes (Fig. 30e) are common among fully grown macrogametocytes	
	………………………………………………………………………………………………………………….	***H. danilewskii*** (Fig. 30e, f) [3, 118, 146]
8 (2)	The nuclei of macrogametocytes usually are in median (Fig. 30g) position and, as a rule, they are closely appressed to the nuclei of infected erythrocytes (Fig. 30g)	
	………………………………………………………………………………………………………………….	***H. lanii* (**Fig. 30g) [3, 23, 85, 118, 147–150]
9 (3)	Dumbbell-shaped growing gametocytes (Fig. 30b, i) are absent or appear only occasionally	
	………………………………………………………………………………………………………………….	***H. dicruri*** (Fig. 30h) [3, 151]
10 (4)	Dumbbell-shaped growing gametocytes, which do not touch the envelope of erythrocytes along their entire margin (Fig. 30i), are common and predominate. Fully grown gametocytes usually do not fill the infected erythrocytes up to their poles (Fig. 30j)	
	………………………………………………………………………………………………………………….	***H. monarchus*** (Fig. 30i, j) [3, 139]

**Table 30:** Key to the *Haemoproteus* species of Passeriformes birds (suborder Passeri) of the families Alaudidae, Cisticolidae, Melanocharitidae, Paridae

Step	Features and species	
1 (8)	Roundish fully grown gametocytes (Fig. 31e, f) are absent	
2 (9)	The nuclei of macrogametocytes usually are in subterminal position and do not locate close to the erythrocyte nuclei (Fig. 31a, b, d, i)	
3 (10)	Fully grown gametocytes, which are closely appressed to the nuclei of erythrocytes but do not touch the envelope of the erythrocytes along their entire margin (Fig. 31h, i), are absent	
4 (5)	The average number of pigment granules in fully grown gametocytes is greater than 15 (Fig. 31a)	
	………………………………………………………………………………………………………………….	***H. wenyoni*** (Fig. 31a) [3, 139]
5 (4)	The average number of pigment granules in fully grown gametocytes is less than 15 (Fig. 31b)	
6 (7)	Dumbbell-shaped gametocytes are present among growing advanced macrogametocytes (Fig. 31c), and such forms are common	
	………………………………………………………………………………………………………………….	***H. majoris***^a^ **(**Fig. 31b, c) [3, 28, 43, 91, 152, 153]
7 (6)	Dumbbell-shape gametocytes (Fig. 31c) are absent	
	………………………………………………………………………………………………………………….	***H. alaudae*** (Fig. 31d) [3, 141]
8 (1)	Fully grown gametocytes are roundish; they markedly deform infected erythrocytes, markedly displace their nuclei, and can even enucleate the host cells (Fig. 31e, f)	
	………………………………………………………………………………………………………………….	***H. parus***^b^ (Fig. 31e, f) [3, 154]
9 (2)	The nuclei of macrogametocytes are median or submedian in positions; they usually adhere to the erythrocyte nuclei (Fig. 31g)	
	………………………………………………………………………………………………………………….	***H. calandrellae*** (Fig. 31g) [3, 155]
10 (3)	Fully grown gametocytes are closely appressed to the nuclei of erythrocytes but do not touch the envelope of the erythrocytes along their entire margin (Fig. 31h, i)	
	………………………………………………………………………………………………………………….	***H. nucleophilus*** (Fig. 31h, i) [3, 156]

^a^Gametocytes of *H. majoris* were occasionally seen in birds of the Sylviidae, Phylloscopidae, Fringillidae, Muscicapidae and Turdidae in Eurasia [28, 43, 91]. This opportunity should be considered during identification of haemoproteids found in birds of these families. See Table 30 for identification of *H. majoris*

^b^Intensity of parasitemia was low in the hapantotype of *H. parus*. Gametocytes of this parasite are similar to gametocytes *Plasmodium (Haemamoeba*) spp. [3, 154]. However, the erythrocytic meronts were not observed in the type material, and molecular characterization of this parasite is not available. It is possible that *H. parus* belongs to *Plasmodium* genus. Further studies of this infection, preferably in samples from the type host and type locality, are needed to unravel this taxonomic uncertainty

**Table 31:** Key to the *Haemoproteus* species of Passeriformes birds (suborder Passeri) of the families Acrocephalidae and Hirundinidae^a^

Step	Features and species	
1 (10)	Gametocytes do not contain gigantic pigment granules (greater than 1.5 μm) (Fig. 32k, l). The average number of pigment granules in fully grown gametocytes is greater than five	
2 (5)	The nuclear material is condensed in fully grown microgametocytes (Fig. 32b, c); the size of the nuclei in microgametocytes does not exceed that of the nuclei in macrogametocytes (compare Fig. 32a and Fig. 32b)	
3 (4)	Fully grown gametocytes are microhalteridial; they usually do not reach poles of infected erythrocytes (Fig. 32a, b). Growing advanced gametocytes, which do not touch envelope of infected erythrocytes along their entire margins (Fig. 32d), are absent. Pigment granules in fully grown gametocytes tend to clamp in one or two louse groups, which often locate close to the gametocyte ends (Fig. 32b), however, the gametocytes with scattered pigment granules are also present (Fig. 32a)	
	………………………………………………………………………………………………………………….	***H. payevskyi*** (Fig. 32a, b) [3, 147, 148, 157]
4 (3)	Fully grown gametocytes are halteridial (Fig. 32c); they usually reach poles of infected erythrocytes (Fig. 32c). Advanced growing gametocytes, which do not touch envelope of infected erythrocytes along their entire margins, are present (Fig. 32d). Pigment granules in fully grown gametocytes are usually scattered (Fig. 32c, d), they do not tend to clamp in one or two louse groups, which locate close to the gametocyte ends (Fig. 32b)	
	………………………………………………………………………………………………………………….	***H. nucleocondensus*** (Fig. 32c, d) [148, 149, 158]
5 (2)	The nuclear material is diffuse (not condensed) in fully grown microgametocytes (Fig. 32e, j); the size of the nuclei in microgametocytes markedly exceeds that of the nucleus in macrogametocytes (compare Fig. 32e and Fig. 32f)	
6 (7)	Gametocytes grow around nuclei of infected erythrocytes and only slightly (if at all) displace the nuclei laterally (Fig. 32g). Fully grown gametocytes markedly enclose the erythrocyte nuclei with their ends and are predominantly close to circumnuclear in form (Fig. 32f); completely circumnuclear forms might occur (Fig. 32e), but do not predominate. Ends of advanced growing gametocytes predominantly are more or less irregular or ameboid in outline (Fig. 32g)	
	………………………………………………………………………………………………………………….	***H. belopolskyi*** (Fig. 32e–g) [3, 147–149, 159, 160]
7 (6)	Gametocytes grow along nuclei of infected erythrocytes and markedly displace the nuclei laterally (Fig. 32h–j). Fully grown gametocytes are broadly halteridial (Fig. 32h, j); they only slightly enclose the erythrocyte nuclei with their ends (Fig. 32h, j); circumnuclear or close to circumnuclear gametocytes (Fig. 32e, f) are absent. Ends of advanced growing gametocytes predominantly are more or less even in outline (Fig. 32i)	
8 (9)	The nuclei of fully grown macrogametocytes are predominantly median or submedian in position (Fig. 32h). Fully grown gametocytes contain the pigment granules of more or less uniform size, and oval to slightly elongate granules of medium size (0.5–1 μm) predominate (Fig. 32h). Large (1–1.5 μm) pigment granules are absent	
	………………………………………………………………………………………………………………….	***H. parahirundinis*** (Fig. 32h) [161]
9 (8)	The nuclei of fully grown macrogametocytes are subterminal in position (Fig. 32i). Fully grown gametocytes contain pigment granules of markedly variable size, and small (< 0.5 μm), medium (0.5–1 μm) and sometimes even large (1–1.5 μm) pigment granules might occur simultaneously in same gametocytes (Fig. 32i, j)	
	………………………………………………………………………………………………………………….	***H. hirundinis*** (Fig. 32i, j) [3, 149, 161]
10 (1)	Gigantic pigment granules are present in fully grown gametocytes (Fig. 32k, l). The average number of pigment granules in fully grown gametocytes is less than five	
	………………………………………………………………………………………………………………….	***H. stellaris*** (Fig. 32k, l) [3, 161, 162]

^a^Gametocytes of *H. majoris* and *H. parabelopolskyi* were occasionally seen in birds of the Acrocephalidae. This opportunity should be considered during identification of haemoproteids found in birds of this family. See Tables 30, 32 for identification of these two species

**Table 32:** Key to the *Haemoproteus* species of Passeriformes birds (suborder Passeri) of the families Leiothrichidae, Phylloscopidae, Pycnonotidae, Sylviidae and Zosteropidae^a^

Step	Features and species	
1 (12)	Advanced dumbbell-shaped gametocytes, which do not touch envelope of infected erythrocytes along their entire margin and have highly constricted (attenuated) central portion and readily thickened roundish ends (Fig. 33j), are absent	
2 (13)	The majority of advanced macrogametocytes do not contain one clear roundish discrete vacuole, which is close to 1 µm in diameter (Fig. 33k, l)	
3 (16)	Fully grown gametocytes are halteridial (Fig. 33a, f, h, g) or close to circumnuclear (Fig. 33c–e); they reach poles of infected erythrocytes and usually occupy poles of the erythrocytes completely (Fig. 33a, c–h)	
4 (17)	Advanced growing macrogametocytes, which are closely appressed to the nuclei of infected erythrocytes but do not touch the envelope of the erythrocytes along their entire margin (Fig. 33n), are absent. Advanced growing gametocytes touch envelope of infected erythrocytes entirely (Fig. 33p) or in one/several points (Fig. 33b)	
5 (20)	Advanced growing gametocytes, whose pellicle in the centre does not extend to the envelope of infected erythrocytes causing a ‘dip’ and giving a dumbbell-like appearance (Fig. 33b), are present and usually common	
6 (7)	Fully grown gametocytes contain large (1–1.5 µm) elongate pigment granules (Fig. 33a), which are common and might predominate in some cells	
	………………………………………………………………………………………………………………….	***H. killangoi*** (Fig. 33a, b) [3, 93, 163, 164]
7 (6)	Fully grown gametocytes usually do not contain large (1–1.5 µm) elongate pigment granules (Fig. 33a); such pigment granules can occur only occasionally in some cells	
8 (9)	Fully grown gametocytes markedly enclose nuclei of infected erythrocytes with their end and tend to assume circumnuclear form (Fig. 33c–e); circumnuclear gametocytes sometimes occur (Fig. 33e), more often among microgametocytes. Fully grown gametocytes are predominantly close to circumnuclear in form (Fig. 33d)	
	………………………………………………………………………………………………………………….	***H. parabelopolskyi***^b^ (Fig. 33c–e) [100, 128, 148, 159]
9 (8)	Fully grown gametocytes slightly enclose nuclei of infected erythrocytes with their ends (Fig. 33f–h), but do not tend to assume circumnuclear form. Fully grown gametocytes are predominantly halteridial (Fig. 33f, g, h)	
10 (11)	Growing and fully grown gametocytes markedly displace nuclei of infected erythrocytes laterally (Fig. 33f, g). Fully grown gametocytes often displace erythrocyte nuclei close to the erythrocyte envelope (Fig. 33g). The average NDR is less than 0.7	
	………………………………………………………………………………………………………………….	***H. leiothrichus, H. homoleiothrichus***^c,d^ (Fig. 33f, g) [7, 165]
11 (10)	Growing and fully grown gametocytes do not markedly displace nuclei of infected erythrocytes laterally (Fig. 33h). Fully grown gametocytes usually do not displace erythrocyte nuclei close to the erythrocyte envelope (Fig. 33g). The average NDR is 0.7 or greater. Early gametocytes often are markedly ameboid in outline (Fig. 33i)	
	………………………………………………………………………………………………………………….	***H. timalus***^d^ (Fig. 33h, i) [3, 139]
12 (1)	Advanced dumbbell-shaped gametocytes, which do not touch envelope of infected erythrocytes along their entire margin and have highly constricted (attenuated) central portion and readily thickened roundish ends (Fig. 33j), are common	
	………………………………………………………………………………………………………………….	***H. philippinensis*** (Fig. 33j) [3, 166]
13 (2)	The majority of advanced macrogametocytes contain a clear roundish discrete vacuole, which is close to 1 µm in diameter (Fig. 33k, l)	
14 (15)	The average number of pigment granules in fully grown gametocytes is greater than 15	
	………………………………………………………………………………………………………………….	***H. vacuolatus*** (Fig. 33k) [63, 100, 167, 168]
15 (14)	The average number of pigment granules in fully grown gametocytes is less than 15	
	………………………………………………………………………………………………………………….	***H. palloris*** (Fig. 33l) [63, 91, 100, 168]
16 (3)	Fully grown gametocytes are microhalteridial, they do not reach poles of infected erythrocytes and do not occupy poles of the erythrocytes (Fig. 33m)	
	………………………………………………………………………………………………………………….	***H. homogeneae*** (Fig. 33m) [100]
17 (4)	Advanced growing macrogametocytes, which are closely appressed to the nuclei of infected erythrocytes but do not touch the envelope of the erythrocytes along their entire margin (Fig. 33n), are common	
18 (19)	Nuclei of fully grown macrogametocytes are subterminal in position (Fig. 33)	
	………………………………………………………………………………………………………………….	***H. otocompsae*** (Fig. 33n, o) [3, 166]
19 (18)	Nuclei of fully grown macrogametocytes are median in position (Fig. 33p)	
	………………………………………………………………………………………………………………….	***H. sanguinis*** (Fig. 33p) [3, 166]
20 (5)	Advanced growing gametocytes, whose pellicle in the centre does not extend to the envelope of infected erythrocytes causing a ‘dip’ and giving a dumbbell-like appearance (Fig. 33b), are absent or appear only occasionally. Advanced growing gametocytes are closely appressed both to the nuclei and envelop of infected erythrocytes (Fig. 33v)	
21 (24)	Fully grown gametocytes do not contain large (greater than 1 µm) pigment granules (Fig. 33t–v). The macrogametocyte cytoplasm is relatively pale-stained and is similar to microgametocytes based this character (compare Fig. 33q and r)	
22 (23)	Fully grown gametocytes contain roundish, small (less than 0.5 µm) pigment granules (Fig. 33q, r). Pigment granules do not change size and shape significantly as parasite matures	
	………………………………………………………………………………………………………………….	***H. pallidulus*** (Fig. 33q, r) [63, 100, 168, 169]
23 (22)	Fully grown gametocytes contain roundish and oval pigment granules (Fig. 33s), which are of medium size (0.5–1.0 μm). Pigment granules readily increase in size as parasite matures	
	………………………………………………………………………………………………………………….	***H. homopalloris*** (Fig. 33s) [63, 168]
24 (21)	Fully grown gametocytes contain large (greater than 1 µm) pigment granules (Fig. 33t–v). Macrogametocytes are readily distinguishable from microgametocytes based on intensity of staining of their cytoplasm (Fig. 33t, u)	
	………………………………………………………………………………………………………………….	***H. zosteropis*** (Fig. 33t–v) [3, 163, 164]

^a^This group of *Haemoproteus* species is difficult to identify due to similar form of growing and mature gametocytes in many species. Visualisation of all gametocyte development stages (young, growing and fully grown) is essential for final conclusion about species identification

^b^*Haemoproteus parabelopolskyi* is common in birds of this group, however *Haemoproteus belopolskyi* might occur in some species of the Sylviidae and Phylloscopidae occasionally. The latter parasite is similar to *H. parabelopolskyi*. These two parasite species can be distinguished due to differences in size of nuclei of fully grown gametocytes [159]; the average area of the nuclei is greater than 3 µm^2^ in *H. belopolskyi* (Fig. 32f), but is less than 3 µm^2^ in *H. parabelopolskyi* (Fig. 33e)

^c^*Haemoproteus majoris* was seen in some species of the Sylviidae and Phylloscopidae. This parasite is morphologically similar to *H. leiothrichus* and *H. homoleiothrichus*. The latter two parasites seem to be of tropical distribution; transmission of *H. majoris* predominantly occurs in countries with temperate and cold climates

^d^*Haemoproteus leiothrichus* and *H. homoleiothrichus* seem to be cryptic species based on the original description of their gametocytes [165]. Taxonomic status of these species requires confirmation with regard to *H. timalus*. Gametocytes of *H. leiothrichus* and *H. homoleiothrichus* are similar morphologically [165], and they are barely distinguishable from gametocytes of *H. timalus* because of overlapping most diagnostic characters. Additionally, all three parasites develop in closely related birds of the family Leiothrichidae. Molecular characterization of *H. timalus* has not been done. Because *H. timalus* has priority in nomenclature, either *H. leiothrichus* or *H. homoleiothrichus* might be synonyms of *H. timalus*. Molecular characterization of *H. timalus* is needed to answer this question. More detail examination of these three parasites is needed

**Table 33:** Key to the *Haemoproteus* species of Passeriformes birds (suborder Passeri) of the families Mimidae, Muscicapidae, Sittidae, Sturnidae and Turdidae^a^

Step	Features and species	
1 (20)	Fully grown gametocytes do not contain large (1–1.5 µm) rod-like, thin pigment granules (Fig. 34o)	
2 (21)	The average number of pigment granules in fully grown gametocytes is less than 20	
3 (24)	Fully grown gametocytes are closely appressed to the nuclei and envelope of infected erythrocytes (Fig. 34c–g). Fully grown gametocytes, which do not touch the erythrocyte envelope along their entire margin (Fig. 34s, t), are absent	
4 (15)	Fully grown macrogametocytes and/or microgametocytes are halteridial (Fig. 34c–e, g); they reach poles of infected erythrocytes and can occupy the poles completely (Fig. 34c–e, g)	
5 (8)	Volutin is present and abundant in gametocytes (Fig. 34a–d). Advanced and fully grown gametocytes are overfilled with volutin granules, which obscure visualization of pigment granules (Fig. 34a–d)	
6 (7)	Advanced growing microgametocytes, which (i) fill the infected erythrocytes up to their poles and (ii) have the pronounced dumbbell-like shape with the portion of the parasite adjacent to the erythrocyte nucleus markedly narrowed (the width of the parasite at this portion is close to 1 µm) (Fig. 34b), are present	
	………………………………………………………………………………………………………………….	***H. attenuatus*** (Fig. 34a, b)^b^ [3, 46, 170]
7 (6)	Advanced growing microgametocytes, which (i) fill the infected erythrocytes up to their poles and (ii) have the pronounced dumbbell-like shape with the portion of the parasite adjacent to the erythrocyte nucleus markedly narrowed (the width of the parasite at this portion is close to 1 µm) (Fig. 34a, b), are absent	
	………………………………………………………………………………………………………………….	***H. balmorali*** (Fig. 34c, d)^b^ [3, 82, 153, 171, 172]
8 (5)	Volutin is absent or scanty. Advanced and fully grown gametocytes are not overfilled with volutin granules (Fig. 34a–d), which obscure visualization of pigment granules	
9 (14)	The dumbbell-shaped macrogametocytes (Fig. 34j) are absent or occur only occasionally among growing advanced macrogametocytes	
10 (11)	The medium size (0.5–1 µm) pigment granules predominate in fully grown gametocytes (Fig. 34e)	
	………………………………………………………………………………………………………………….	***H. pastoris*** (Fig. 34e) [3, 128, 146, 173]
11 (10)	The medium size (0.5–1 µm) pigment granules (Fig. 34e) are absent or occur only occasionally in fully grown gametocytes; small (less than 0.5 µm) pigment granules (Fig. 34f-i) readily predominate in fully grown gametocytes	
12 (13)	Nearly fully grown gametocytes, which do not touch the envelope of infected erythrocytes along their entire margin (Fig. 34h), are absent. Nearly fully grown gametocytes are closely appressed to the envelope of infected erythrocytes (Fig. 34f)	
	………………………………………………………………………………………………………………….	***H. homominutus*** (Fig. 34f, g) [63, 100]
13 (12)	Nearly fully grown gametocytes, which do not touch the envelope of infected erythrocytes along their entire margin (Fig. 34h), are common and predominate	
	………………………………………………………………………………………………………………….	***H. kairullaevi*** (Fig. 34h, i) [3, 174]
14 (9)	The dumbbell-shaped macrogametocytes (Fig. 34j) are common and predominate among growing advanced macrogametocytes	
	………………………………………………………………………………………………………………….	***H. sittae*** (Fig. 34j) [3]
15 (4)	Fully grown gametocytes are microhalteridial (Fig. 34l–n); they do not reach poles of infected erythrocytes and do not occupy the poles completely (Fig. 34l–n)	
16 (19)	The average number of pigment granules in fully grown gametocytes is greater than 10	
17 (18)	Macrogametocyte nuclei predominantly assume the central or subcentral position (Fig. 34k). The asymmetrical position of advanced growing gametocytes in regard of erythrocyte nuclei (Fig. 34k) is characteristic	
	………………………………………………………………………………………………………………….	***H. asymmetricus*** (Fig. 34k) [63]
18 (17)	Macrogametocyte nuclei predominantly assume the subterminal position (Fig. 34l). The asymmetrical position of advanced growing gametocytes in regard of erythrocyte nuclei (Fig. 34k) is not characteristic	
	………………………………………………………………………………………………………………….	***H. fallisi*** (Fig. 34l) [3, 63, 175]
19 (16)	The average number of pigment granules in fully grown gametocytes is less than 10. Macrogametocyte nuclei assume predominantly terminal or close to terminal position (Fig. 34m). The asymmetrical position of advanced growing gametocytes in regard of erythrocyte nuclei (Fig. 34k) is not characteristic	
	………………………………………………………………………………………………………………….	***H. minutus*** (Fig. 34m, n) [3, 16, 63, 82, 100, 147, 173]
20 (1)	Fully grown gametocytes contain large (1–1.5 µm) rod-like, thin pigment granules (Fig. 34o)	
	………………………………………………………………………………………………………………….	***H. beckeri*** (Fig. 34o) [3, 176]
21 (2)	The average number of pigment granules in fully grown gametocytes is greater than 20	
22 (23)	Advanced growing gametocytes of dumbbell-shape (Fig. 34p, q) are present and common	
	………………………………………………………………………………………………………………….	***H. neseri*** (Fig. 34p, q) [3, 79]
23 (22)	Advanced growing gametocytes of dumbbell-shape (Fig. 34p, q) are absent	
	………………………………………………………………………………………………………………….	***H. nipponensis*** (Fig. 34r) [3, 139]
24 (3)	Fully grown gametocytes are closely appressed to the nuclei of infected erythrocytes, but do not touch the erythrocyte envelope along their entire margin (Fig. 34s, t)	
	………………………………………………………………………………………………………………….	***H. pallidus*** (Fig. 34s, t) [3, 63, 100, 147, 148, 153, 168, 173]

^a^Gametocytes of *Haemoproteus majoris* were occasionally reported in birds of the Muscicapidae and Turdidae. This opportunity should be considered during identification of haemoproteids found in birds of these families (see keys to the parasites of these bird families)

^b^Fully grown gametocytes of *H. attenuatus* and *H. balmorali* (Fig. 34c, d) are very similar. However, these species are readily distinguishable due to the different mode of the gametocyte growth. Mainly, the attenuated growing gametocytes develop in *H. attenuatus* (Fig. 34b) but do not develop in *H. balmorali*. During species identification, this feature worth attention because co-infection of these parasites was reported

**Table 34:** Key to the *Haemoproteus* species of Passeriformes birds (suborder Passeri) of the families Dicaeidae, Estrildidae, Fringillidae, Motacillidae, Nectariniidae, Passeridae and Ploceidae

Step	Features and species^a^	
1 (30)	The readily distinguishable sickle-shaped space is absent between the growing advanced gametocyte and the nucleus of infected erythrocyte (Fig. 35u, v); advanced growing gametocytes never assume concave shapes (Fig. 35u, v)	
2 (31)	Fully grown gametocytes usually do not assume rhabdosomal form (Fig. 35w, x); if rhabdosomal gametocytes develop occasionally, they never predominate. The fully grown gametocytes usually do not enucleate infected erythrocytes (Fig. 35x)	
3 (6)	Fully grown microgametocytes contain markedly compressed (not diffuse) nuclei (Fig. 35a, c). The area of microgametocyte nuclei (Fig. 35a, c) is similar or even less than the area of nuclei of fully grown macrogametocytes (Fig. 35b)	
4 (5)	Fully grown microgametocytes contain markedly compressed nuclei, which usually assume band-like shapes and are closely associated with the envelope of infected erythrocytes (Fig. 35a)	
	………………………………………………………………………………………………………………….	***H. nucleofascialis*** (Fig. 35a, b) [177]
5 (4)	Fully grown microgametocytes contain markedly compressed nuclei, which do not assume band-like shapes and usually are not associated with the envelope of infected erythrocytes, but locate free in the cytoplasm (Fig. 35c)	
	………………………………………………………………………………………………………………….	***H. micronuclearis*** (Fig. 35c) [177]
6 (3)	Fully grown microgametocytes contain large diffuse nuclei (Fig. 35d). The area of microgametocyte nuclei is greater that the area of nuclei of fully grown macrogametocytes (compare Fig. 35d and e)	
7 (32)	Fully grown gametocytes, which are closely appressed to the nuclei of infected erythrocytes but do not touch the envelope of the erythrocytes along their entire margin (Fig. 35y), are absent	
8 (33)	Advanced growing gametocytes (size significantly greater than erythrocyte nuclei, Fig. 35z), which are closely appressed to the nuclei of infected erythrocytes but do not touch the envelope of the erythrocytes along their entire margin (Fig. 35z), are absent	
9 (36)	Fully grown gametocytes usually do not contain large (1–1.5 µm) pigment granules (Fig. 35aa–cc, ff, gg). Such pigment granules might occur only occasionally in fully grown gametocytes	
10 (11)	Fully grown gametocytes are microhalteridial (Fig. 35d, e) or close to microhalteridial; they usually do not reach poles of infected erythrocytes (Fig. 35d, e) and do not occupy the poles of erythrocytes completely (Fig. 35f). Nuclei of fully grown macrogametocytes are predominantly of median position or close to the median position (Fig. 35e)	
	………………………………………………………………………………………………………………….	***H. africanus*** (Fig. 35d, e) [3, 178]
11 (10)	Fully grown gametocytes are halteridial (Fig. 35h, m, n, q, r, aa) or close to circumnuclear (Fig. 35f, g); they reach poles of infected erythrocytes and occupy the poles of erythrocytes completely. Nuclei of fully grown macrogametocytes are predominantly of subterminal position (Fig. 35f, h, m, r)	
12 (21)	Dumbbell-shaped macrogametocytes (Fig. 35g, k, l) are common and often predominate among growing advanced macrogametocytes	
13 (14)	Fully grown gametocytes markedly enclose the nuclei of infected erythrocytes with their ends (Fig. 35f) and tend to assume circumnuclear form (Fig. 35g). Circumnuclear gametocytes develop occasionally. The rod-like (thin) pigment granules are common in advanced and fully grown gametocytes (Fig. 35f, g)	
	………………………………………………………………………………………………………………….	***H. homobelopolskyi*** (Fig. 35f, g) [177]
14 (13)	Fully grown gametocytes only slightly enclose nuclei of infected erythrocytes with their ends (Fig. 35h); they do not tend to assume circumnuclear form. Circumnuclear gametocytes do not develop. Pigment granules in fully grown gametocytes predominantly are roundish or oval in form (Fig. 35h); rod-like (thin) pigment granules (Fig. 35f, g) are not characteristic	
15 (16)	Young gametocytes (size of nuclei of erythrocytes) first adhere to the envelope of erythrocytes and then grow towards erythrocyte nuclei (Fig. 35i)	
	………………………………………………………………………………………………………………….	***H. passeris*** (Fig. 35h, i) [3, 145]
16 (15)	Young gametocytes (size of nuclei of erythrocytes) first adhere to the erythrocyte nuclei and then grow towards erythrocyte envelope (Fig. 35j)	
17 (18)	Advanced growing macrogametocytes often are dumbbell-shaped and assume a distinct linear form (Fig. 35k), they deform infected erythrocytes by causing envelop protrusions, which are located in the non-invaded cytoplasmic region of the erythrocytes (Fig. 35k)	
	………………………………………………………………………………………………………………….	***H. fringillae*** (Fig. 35j, k) [3, 172]
18 (17)	Advanced macrogametocytes often are dumbbell-shaped, but do not assume a distinct linear form (Fig. 35k) and do not deform infected erythrocytes by causing envelop oval protrusions, which are located in the non-invaded cytoplasmic region of the erythrocytes (Fig. 35k)	
19 (20)	The average number of pigment granules in fully grown gametocytes is less than 15. Markedly attenuated growing advanced dumbbell-shaped gametocytes (Fig. 35l) are common	
	………………………………………………………………………………………………………………….	***H. dolniki*** (Fig. 35l) [3]
20 (19)	The average number of pigment granules in fully grown gametocytes is greater than 15. Markedly attenuated growing dumbbell-shaped gametocytes (Fig. 35l) are not characteristic	
	………………………………………………………………………………………………………………….	***H. queleae*** (Fig. 35m) [3, 178]
21 (12)	Dumbbell-shaped macrogametocytes (Fig. 35g k, l) are absent or occur only occasionally; they never predominate among growing macrogametocytes	
22 (27)	The average number of pigment granules in fully grown gametocytes is less than 18	
23 (24)	Fully grown gametocytes predominantly contain small pigment granules (less than 0.5 µm), which are dust-like in appearance; medium-size (0.5–1 µm) pigment granules might occur only occasionally	
	………………………………………………………………………………………………………………….	***H. dicaeus*** (Fig. 35n) [3, 156]
24 (23)	Fully grown gametocytes predominantly contain medium-size (0.5–1 µm) pigment granules	
25 (26)	The average number of pigment granules in fully grown macrogametocytes is greater than 12. The nuclei of fully grown broadly halteridial macrogametocytes (Fig. 35o) often lie free in the cytoplasm and do not adhere to the envelope of infected erythrocytes (Fig. 35o). Fully grown gametocytes markedly displace nuclei of infected erythrocytes both laterally (Fig. 35o) and sometimes also towards one pole of erythrocytes (Fig. 35p); they occasionally can enucleate the host cells, but gametocytes in enucleated erythrocytes are always rare and might occur only in preparations where fully grown mature forms predominate	
	………………………………………………………………………………………………………………….	***H. tartakovskyi*** (Fig. 35o, p) [3, 148, 150, 172, 179]
26 (25)	The average number of pigment granules in fully grown macrogametocytes is less than 12. The nuclei of fully grown macrogametocytes always adhere to the envelope of infected erythrocytes (Fig. 35q). Fully grown gametocytes markedly displace nuclei of infected erythrocytes laterally (Fig. 35q), but never towards one pole of erythrocytes (Fig. 35p), they never enucleate the host cells	
	………………………………………………………………………………………………………………….	***H. anthi*** (Fig. 35q) [3]
27 (22)	The average number of pigment granules in fully grown gametocytes is greater than 18 (Fig. 35r–t)	
28 (29)	Fully grown gametocytes are broadly halteridial (Fig. 35r); circumnuclear (Fig. 35t) fully grown gametocytes do not occur	
	………………………………………………………………………………………………………………….	***H. orizivorae*** (Fig. 35r) [3, 178]
29 (28)	Fully grown gametocytes are markedly pleomorphic, and both broadly halteridial (Fig. 35s) and circumnuclear (Fig. 35t) forms might occur simultaneously	
	………………………………………………………………………………………………………………….	***H. globulosus*** (Fig. 35s, t) [3]
30 (1)	A readily distinguishable sickle-shaped space often is present between the growing advanced gametocyte and the nucleus of infected erythrocyte (Fig. 35u, v); due to this feature growing advanced gametocytes often assume concave shapes (Fig. 35u, v)	
	………………………………………………………………………………………………………………….	***H. concavocentralis*** (Fig. 35u, v) [63, 100, 121, 168]
31 (2)	Fully grown gametocytes often assume rhabdosomal form (Fig. 35w, x). Fully grown gametocytes often enucleate infected erythrocytes (Fig. 35x)	
	………………………………………………………………………………………………………………….	***H. uraeginthus*** (Fig. 35w, x) [3, 178]
32 (7)	Fully grown gametocytes, which are closely appressed to the nuclei of infected erythrocytes but do not touch the envelope of the erythrocytes along their entire margin (Fig. 35y), are common. Spaces between the fully grown gametocytes and envelope of infected erythrocytes are conspicuous (Fig. 35y)	
	………………………………………………………………………………………………………………….	***H. paranucleophilus*** (Fig. 35y) [177]
33 (8)	Advanced growing gametocytes (size significantly greater than erythrocyte nuclei, Fig. 35z), which are closely appressed to the nuclei of infected erythrocytes but do not touch the envelope of the erythrocytes along their entire margin (Fig. 35z), are common	
34 (35)	Dumbbell-shaped forms (Fig. 35l, z) are common and predominate among growing advanced gametocytes	
	………………………………………………………………………………………………………………….	***H. cyanomitrae*** (Fig. 35z) [180]
35 (34)	Dumbbell-shaped forms (Fig. 35l, z) usually are absent or occur only occasionally among growing advanced gametocytes	
	………………………………………………………………………………………………………………….	***H. sequeirae*** [3]
36 (9)	Fully grown gametocytes contain large (1–1.5 µm) pigment granules (Fig. 35aa–dd, ff, gg)	
37 (40)	Fully grown gametocytes markedly enclose nuclei of infected erythrocytes with their ends and tend to encircle the nuclei completely (Fig. 35aa–cc). Circumnuclear gametocytes might occur (Fig. 35bb), but usually are rare	
38 (39)	The large (1–1.5 µm) elongate pigment granules (Fig. 35aa, bb) are present in fully grown gametocytes; the large (1–1.5 µm) roundish (pea-like in shape) pigment granules (Fig. 35cc) are absent or occur only occasionally	
	………………………………………………………………………………………………………………….	***H. magnus*** (Fig. 35aa, bb) [3, 85]
39 (38)	The large (1–1.5 µm) roundish (pea-like in shape) pigment granules (Fig. 35cc) are present and predominate in fully grown gametocytes	
	………………………………………………………………………………………………………………….	***H. macropigmentatus*** (Fig. 35cc) [3]
40 (37)	Fully grown gametocytes slightly enclose nuclei of infected erythrocytes with their ends (Fig. 35dd, gg), but do not tend to encircle the nuclei completely (Fig. 35bb). Circumnuclear gametocytes (Fig. 35bb) are absent	
41 (42)	Dumbbell-shaped growing gametocytes (Fig. 35ee) are common. One or both ends of fully grown gametocytes are more or less rounded (Fig. 35dd, ff)	
	………………………………………………………………………………………………………………….	***H. motacillae*** (Fig. 35dd–ff) [3, 16, 153, 181]
42 (41)	Dumbbell-shaped growing gametocytes (Fig. 35ee) are absent or occur only occasionally. Both ends of fully grown gametocytes usually are more or less pointed (Fig. 35gg)	
	………………………………………………………………………………………………………………….	***H. bubalornis*** (Fig. 35gg) [3, 178]

^a^Gametocytes of *H. majoris* were occasionally found in birds of the Fringillidae. This opportunity should be considered during identification of haemoproteids in birds of this family. See Table 30 for identification of *H. majoris*

**Table 35:** Key to the *Haemoproteus* species of Passeriformes birds (suborder Passeri) of the families Emberizidae, Icteridae, Parulidae, Passerellidae and Thraupidae

Step	Features and species	
1 (5)	The nuclei assume predominantly subterminal or terminal position in fully grown macrogametocytes (Fig. 36a, b, d). Dumbbell-shaped growing gametocytes (Fig. 36a, d) are common	
2 (6)	Advanced dumbbell-shaped gametocytes, which do not touch envelope of erythrocytes along their entire margin (Fig. 36d), are absent. Advanced dumbbell-shaped gametocytes adhere to the envelope of erythrocytes by their ends (Fig. 36a). Dumbbell-shaped microgametocytes are common	
3 (4)	Advanced and fully grown gametocytes markedly deform infected erythrocytes by causing balloon-like protrusions which are located in the non-invaded cytoplasmic region of the erythrocytes (Fig. 36a); as a result of this deformation, the infected erythrocytes assume a ‘gravid’ shape (Fig. 36a)	
	………………………………………………………………………………………………………………….	***H. erythrogravidus*** (Fig. 36a) [182]
4 (3)	Advanced and fully grown gametocytes do not deform infected erythrocytes by causing balloon-like protrusions which are located in the non-invaded cytoplasmic region of the erythrocytes (Fig. 36a). Infected erythrocytes do not assume a ‘gravid’ shape (Fig. 36b)	
	………………………………………………………………………………………………………………….	**Group of *H. coatneyi*** ^a^ (Fig. 36b) [3, 62, 182]
5 (1)	The nuclei assume predominantly median position in fully grown macrogametocytes (Fig. 36c). Dumbbell-shaped growing gametocytes (Fig. 36a, d) are absent	
	…………………………………………………………………………………………………………………	***H. nucleocentralis*** (Fig. 36c) [183]
6 (2)	Advanced dumbbell-shaped macrogametocytes, which do not touch envelope of erythrocytes along their entire margin (Fig. 36d), are present. Dumbbell-shaped microgametocytes are absent or develop only occasionally	
	………………………………………………………………………………………………………………….	***H. quiscalus*** (Fig. 36d) [3, 59]

^a^Based on the available information [3, 184] and examination of the type and voucher preparations, gametocytes of *H. coereba*, *H. paruli* and *H. thraupi* are morphologically similar to *H. coatneyi*. Judging by the scattered distribution of *H. coatneyi* in phylogenetic trees, this morphospecies might be a complex of several cryptic species [3, 62, 182]. Gametocytes of these four parasites are similar morphologically, indicating possible existence of cryptic speciation, however, more detail analysis of their blood stages in type vertebrate hosts is preferable for confirmation of their taxonomic status. The examination of type material showed that the available specimens are insufficient to answer this question; additional samples with single infections of *H. coereba*, *H. paruli, H. thraupi* and *H. coatneyi* from their type vertebrate hosts are needed to solve this taxonomical uncertainty. This is an important question due to broad distribution of parasites of *H. coatneyi* group in American birds. Molecular characterizations of *H. paruli* (GenBanc accession AF465563, lineage TABI02 in MalAvi database) and *H. thraupi* (AF465583, PIOLI01) were not supported by gametocyte morphological data [185]. Furthermore, DNA sequences were obtained from non-type avian hosts, so the available barcodes of these two parasites remain questionable and need support by investigation of these pathogens in their type vertebrate hosts

**Table 36:** Mitochondrial cytochrome *b* gene sequences, which have been developed for molecular detection and identification (barcoding) of avian *Haemoproteus* parasites

Parasite species	GenBank accession and lineage code (in parentheses)^a^	References^b^
*H. abdusalomovi*	Not available	Not available
*H. aegithinae*	Not available	Not available
*H. africanus*	Not available	Not available
*H. alaudae*	Not available	Not available
*H. ammoperdix*	Not available	Not available
*H. anthi*	Not available	Not available
*H. antigonis*	KX223839 (GRUAME01)^c^, KX223842 (GRUAME02), KX223843 ((GRUAME03)	[190]
*H. apodus*	Not available	Not available
*H. archilochus*	Not available	Not available
*H. asymmetricus*	MW492355 (TUPHI01)	[63]
*H. attenuatus*	DQ630007 (LULU01)^d^, DQ451431 (LULU01), AY393807 (ROBIN01), KJ488597 (ROBIN01)	[16, 85, 147, 170]
*H. balearicae*	Not available	Not available
*H. balmorali*	DQ630008 (SFC1), DQ060770 (SFC1), JX026912 (SFC1), DQ630014 (hCOLL3)	[147, 148, 172]
*H. beckeri*	Not available	Not available
*H. belopolskyi*	DQ630006 (HIICT1), JX026904 (HIICT1), JX026909 (HIICT3), MN025422 (HIICT1), KJ627801 (HIICT1), AF254969 (MW1)	[147–149, 159, 160]
*H. bennetti*	Not available	Not available
*H. bilobata*	Not available	Not available
*H. borgesi*	Not available	Not available
*H. brachiatus*	MK580170 (LK03)	[100]
*H. bubalornis*	Not available	Not available
*H. bucconis*	Not available	Not available
*H. bucerotis*	Not available	Not available
*H. bukaka*	KX100323 (CRALOU01)	[143]
*H. burhini*	Not available	Not available
*H. buteonis*	Not available	Not available
*H. calandrellae*	Not available	Not available
*H. caprimulgi*	Not available	Not available
*H. catharti*	MF953291 (CATAUR01)^e^	[69]
*H. centropi*	Not available	Not available
*H. ciconiae*	Not available	Not available
*H. circumnuclearis*	Not available	Not available
*H. clamatori*	Not available	Not available
*H. coatneyi*	KT698210 (ARBRU02), KM211350 (ANSOM01), KF537292 (ARBRU01), KT698210 (ARBRU02), KF537309 (ATPAL02), KF537326 (PIOLI03), KF537285 (TANIG01), KF537283 (TANVAS02), KF537327 (ZOCAP13)^f^	[62, 182]
*H. columbae*	KU131584 (COLI03), KU1311585 (COQUI05), KJ644778 (HAECOL01)), KF537314 (HAECOL01)	[62, 81, 191–193]
*H. concavocentralis*	GQ396708 (HAWF2)	[91]
*H. contortus*	Not available	Not available
*H. coraciae*	KU297278 (CORGAR01)	[94]
*H. cornuata*	Not available	Not available
*H. cracidarum*	Not available	Not available
*H. crumenium*	Not available	Not available
*H. cublae*	Not available	Not available
*H. cuculis*	Not available	Not available
*H. cyanomitrae*	EU810741 (CYAOLI03), FJ404696 (CYAOLI05), FJ404698 (CYAOLI06)	[180]
*H. danilewskii*	DQ451411 (COCOR01)	[85]
*H. dicaeus*	Not available	Not available
*H. dicruri*	Not available	Not available
*H. dolniki*	Not available	Not available
*H. elani*	Not available	Not available
*H. enucleator*	DQ659592 (ALCLEU01)^g^	[194]
*H. erythrogravidus*	KF537315 (ZOCAP01), KF537329 (ZOCAP14)	[182]
*H. eurylaimus*	Not available	Not available
*H. eurystomae*	Not available	Not available
*H. fallisi*	Not available	Not available
*H. formicarius*	Not available	Not available
*H. forresteri*	Not available	Not available
*H. fringillae*	DQ060764 (CCF3)	[43, 172]
*H. furnarius*	Not available	Not available
*H. fuscae*	EU810722 (CELEC01)	[93]
*H. gallinulae*	Not available	Not available
*H. gavrilovi*	KP462688 (MEAPI02)	[91]
*H. globulosus*	Not available	Not available
*H. goodmani*	Not available	Not available
*H. greineri*	Not available	Not available
*H. halcyonis*	Not available	Not available
*H. handai*	Not available	Not available
*H. herodiadis*	Not available	Not available
*H. hirundinis*	KJ499183 (DELURB01), MN025423 (DELURB2)	[128, 149, 161]
*H. homobelopolskyi*	HQ386240 (PLOMEL01), HQ386241 (PLOMEL02)	[177]
*H. homogeneae*	MK580174 (SYAT16)	[100]
*H. homohandai*	KY783725 (ARCHL01)	[125]
*H. homoleiothrichus*	KY623721 (TROERY02)^h^	[165]
*H. homominutus*	MK580175 (CUKI1)	[100]
*H. homopalloris*	MH513601 (PHSIB2)	[168]
*H. homopicae*	MK580172 (GAGLA07)	[100]
*H. homovelans*	GU085195 (PICAN02)	[121]
*H. indicator*	Not available	Not available
*H. iwa*	JF833050 (FREMIN01)	[131]
*H. janovyi*	Not available	Not available
*H. jenniae*	CREFURO1 (JN827318)	[72]
*H. kairullaevi*	Not available	Not available
*H. killangoi*	JN661945 (ZOSMAD01), KT777738 (ZOSLAT07), KX604234 (ZOSLAT10), KX604236 (ZOSXAN03)	[93, 164]
*H. krylovi*	Not available	Not available
*H. lairdi*	Not available	Not available
*H. lanii*	DQ451429 (RSB2) DQ630010 (RB1), MN025425 (RB1), JX026907 (RB1), KR049265 (RB1), KU529942 (RB1), DQ630011 (RBS2), DQ630012 (RBS4)	[23, 85, 147–150]
*H. larae*	AB604310 (SPMAG12), LC230123 (LARCRA02), LC230122 (NUMPHA01)	[74]
*H. leiothrichus*	KY623720 (TROERY01)^h^	[165]
*H. lophortyx*	Not available	Not available
*H. macropigmentatus*	Not available	Not available
*H. macrovacuolatus*	KJ175078 (DENAUT01), KJ592828 (DENAUT01), KJ499987 (CA1017)	[55]
*H. madagascariensis*	Not available	Not available
*H. magnus*	DQ451426 (CCF7)	[85]
*H. majoris*	AF254977 (PARUS1), AY831755 (WW2), MN219405 (PHYBOR04)^i^	[28, 43]
*H. mansoni*	Not available	Not available
*H. manwelli*	KP462687 (MEAPI01)	[91]
*H. megapodius*	Not available	Not available
*H. meropis*	Not available	Not available
*H. micronuclearis*	HQ386235 (RBQ11), HQ386236 (VILWE1), HQ386237 (PLONIG01), HQ386238 (PLONIG02), HQ386239 (PLONIG03)	[177]
*H. minchini*	KU160476 (CORCRI01)	[115]
*H. minutus*	DQ630013 (TURDUS2), DQ060772 (TURDUS2), JX026900 (TURDUS2), DQ060772 (TURDUS2)	[16, 147, 148, 172]
*H. monarchus*	Not available	Not available
*H. montezi*	Not available	Not available
*H. motacillae*	AF495579 (YWT01), AF495580 (YWT02), DQ368371 (YWT03), DQ368372 (YWT05), KC568475 (YWT06)	[16, 181]
*H. multipigmentatus*	GU296216 (JH003W), GU296215 (ZEGAL05), GU296224 (JH3008W)	[82, 83]
*H. multivolutinus*	JX275888 (TURTYM01)	[82]
*H. neseri*	Not available	Not available
*H. nettionis*	Not available	Not available
*H. nipponensis*	Not available	Not available
*H. nisi*	Not available	Not available
*H. noctuae*	KP794612 (CIRCUM01)	[130]
*H. nucleocentralis*	MT724553 (TANDES01)	[184]
*H. nucleocondensus*	JX026901 (GRW1), MN025424 (GRW1)	[148, 149, 158]
*H. nucleofascialis*	HQ386243 (MALRUB02), HQ386244 (PLOMEL03)	[177]
*H. nucleophilus*	Not available	Not available
*H. orioli*	Not available	Not available
*H. orizivorae*	Not available	Not available
*H. ortalidum*	KX171627 (PENOBS01)	[195]
*H. otocompsae*	Not available	Not available
*H. pachycephalus*	Not available	Not available
*H. pallidulus*	AY831752 (SYAT03)	[169]
*H. pallidus*	DQ630004 (hPFC1), JX026899 (hPFC1), DQ630005 (COLL2), DQ060771 (hSFC3)	[43, 147, 148, 172]
*H. palloris*	AF254971 (WW1)	[91]
*H. palumbis*	Not available	Not available
*H. parabelopolskyi*	AY831751 (SYAT2), AY831762 (SYAT16), AF495575 (SYBOR01), JX026902 (SYBOR01), AY831750 (SYAT01), AY831751 (SYAT02), KJ499184 (SYNIS01)	[100, 128, 148, 159]
*H. parahirundinis*	MT119966 (HIRUS05)	[161]
*H.paramultipigmentatus*	FJ462657 (COLPAS03), JN788939 (COLPAS05)	[82]
*H. paranucleophilus*	HQ386242 (MALRUB01)	[177]
*H. paraortalidum*	MH036944 (ABUJAC01)	[108]
*H. parus*	Not available	Not available
*H. passeris*	DQ451420 (P37), DQ451421 (P102), DQ451422 (P89), DQ451423 (P138), HM146898 (PADOM05), GU065651 (PAHIS01)	[85, 196]
*H. pastoris*	KJ499185 (LAMPUR01), KU752568 (LAMPUR01)	[128, 173]
*H. payevskyi*	AF254968 (RW01), DQ630009 (RW01), JX026905 (RW01)	[147, 148]
*H. pelouroi*	Not available	Not available
*H. philippinensis*	Not available	Not available
*H. picae*	Not available^j^	Not available
*H. pittae*	Not available	Not available
*H. plataleae*	Not available	Not available
*H. porzanae*	Not available	Not available
*H. pratasi*	Not available	Not available
*H. psittaci*	Not available	Not available
*H. pteroclis*	Not available	Not available
*H. ptilotis*	KP721986 (LICHRYS01), JX021554 (LICFRE03), KP721990 (MELLEW03), KP721987 (MELALB01), KP721988 (MELALB02), KP721989 (MELALB03), AY714177 (MELLEW01), KP721992 (NMIN01)	[138]
*H. pulcher*	OL906298 (CARCRI02) ^k^	[67]
*H. queleae*	Not available	Not available
*H. quiscalus*	Not available	Not available
*H. rileyi*	Not available	Not available
*H. rotator*	Not available	Not available
*H. sacharovi*	JX073258 (MODO1)	[45]
*H. sanguinis*	DQ451409 (BUL1), DQ451410 (BUL4)	[85]
*H. scolopaci*	Not available	Not available
*H. sequeirae*	Not available	Not available
*H. sittae*	Not available	Not available
*H. skuae*	Not available	Not available
*H. souzalopesi*^l^	Not available	Not available
*H. stableri*	Not available	Not available
*H. stellaris*	Not available	Not available
*H. syrnii*	KJ451480 (OTSCO05), KF279523 (STAL02), DQ451424 (STSEL01), KP794611 (CULKIB01)^m^	[128–130]
*H. tartakovskyi*	AY393806 (SISKIN01), JX026908 (SISKIN01), JX026903 (HAWF1), GU289671 (ALARV1), GU289672 (ALARV2), GU289673 (ALARV3)	[148, 150, 172, 179]
*H. telfordi*	Not available	Not available
*H. thereicerycis*	Not available	Not available
*H. timalus*	Not available	Not available
*H. tinnunculi*^n^	MK580171 (FALSUB01)	[100]
*H. trochili*	Not available	Not available
*H. trogonis*	Not available	Not available
*H. turtur*	DQ451425 (STSEN1)	[85]
*H. tyranni*	Not available	Not available
*H. undulatus*	Not available	Not available
*H. upupae*	Not available	Not available
*H. uraeginthus*	Not available	Not available
*H. vangii*	Not available	Not available
*H. vacuolatus*	EU770153 (ANLAT02)	[167]
*H. valkiunasi*	GQ404559 (FREAND01)	[46]
*H. velans*	MH311671 (MELCAR01), MH311672 (MELSTR01)	[120]
*H. vireonis*	FJ168561 (VIGIL01), KF482350 (VIOLI05), KF537331 (VIOLI06)	[62, 136]
*H. wenyoni*	Not available	Not available
*H. witti*	JQ988105 (TROAED20), KF767420 (TROAED20)	[61, 62]
*H. xantholaemae*	Not available	Not available
*H. zosteropis*	JX021550 (ZOSLAT04), KX604237 (ZOSXAN02), KX604235 (ZOSLAT11), FJ664153 (ZOSSTE01)	[164]

^a^Mainly DNA sequences, for which parasite species identity was supported by morphological analysis were included in this table. In cases of the identical DNA sequences of same morphospecies, the preference was given to the GenBank accessions, which directed a reader straightforward to articles containing morphological parasite descriptions. This simplifies the parasite identification using GenBank information. Where possible, the codes of lineages were given according to MalAvi database

^b^References of articles containing description of parasite morphology and/or discussion on their molecular characterization, which are valuable for species identification

^c^Disputable quality of the data (consensus inferred from diverse partial *cytb* and *cox1* gene sequences obtained from mixed infections) calls for support of the barcoding sequences of *H. antigonis* (see [9, 190])

^d^Re-examination of blood films from *Luscinia luscinia* showed that the lineage LULU01 belongs to *H. attenuatus,* but not to *H. balmorali* as reported formerly [147]

^e^Molecular characterization of *H. catharti* needs confirmation due to coinfections of several haemosporidian species in samples, which were used for the parasite molecular characterization [69]

^f^See Table 35 for comments in regard of molecular characterization of *H. paruli* and *H. thraupi*

^g^Molecular characterization of *H. enucleator* needs confirmation because it was not supported by morphological data, and the DNA sequence was received from parasites found in non-type avian host [194]

^h^In regard of molecular identification of *H. homoleiothrichus* and *H. leiothrichus,* see comments in Table 32

^i^Lineages CCF5, CWT4, PHSIB1 were formerly attributed to *H. majoris* [43], but the morphological proof remains insufficient due to possible co-infection with other haemoproteids. Further research is needed to validate this action

^j^Molecular characterization of *H. picae* was incorrect in [197] because it was developed using samples, which came from non-type host (*Picoides pubescens*, Piciformes). *Haemoproteus picae* was originally described from *Pica pica* (Passeriformes) and ideally should be characterised molecularly using the samples from this avian host

^k^Molecular characterization of *H. pulcher* needs confirmation due to possible presence of overlapped sequences [67]

^l^*Haemoproteus souzalopesi* likely belongs to *Plasmodium*. See comments in Table 26

^m^Status of *H. ilanpapernai* [129] remains unclear; this parasite might belong to *H. syrnii* group (see comments in Table 24)

^*n*^*Haemoproteus obainae* and *H. deharoi* are probable synonyms of *H. tinnunculi.* See comments in Table 15

**Fig. 39:**
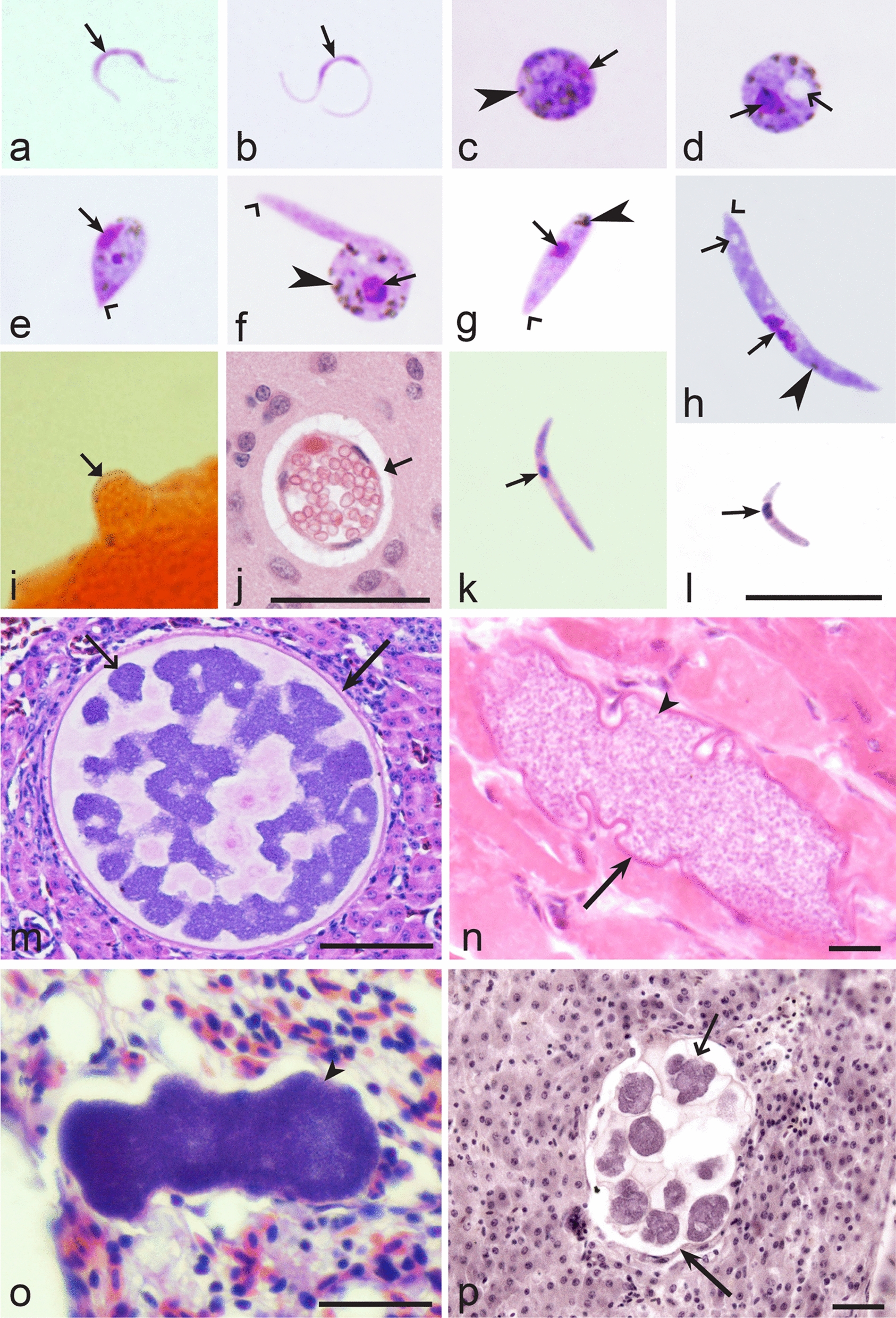
Examples of marked variation in morphological characters of different *Haemoproteus* species during sporogony (**a**–**l**) and exo-erythrocytic development (**m**–**p**). Microgametes (**a**, **b**), zygotes (**c**, **d**), developing ookinetes (**e**, **f**), fully grown ookinetes (**g**, **h**), oocysts (**i**, **j**), sporozoites (**k**, **l**), exo-erythrocytic meront (**o**) and megalomeronts (**m, n, p**) of *H. minutus* (**a, g**), *H. tartakovskyi* (**b**, **c**, **h**, **k**), *H. fringillae* (**d**, **i**), *H. palumbis* (**j**, **l**), *H. majoris* (**m**), *H. mansoni* (**n**), *H. attenuatus* (**o**) and *H. passeris* (**p**). Note that these *Haemoproteus* species are readily different not only on gametocyte stages (see Figs. 13, 17, 31, 34, 35), but also due to the following characters of their sporogonic and exo-erythrocytic stages: length of microgametes (**a**, **b**), mode of the cytoplasm vacuolization in zygotes (**c**, **d**), mode of elongation during initial stage of ookinete development (**e**, **f**), size and form of fully grown ookinetes (**g**, **h**), oocyst structure (**i**, **j**), size and form of sporozoites (**k**, **l**), form of exo-erythrocytic stages (**m**–**p**). Microgametes (**a**, **b**), ookinetes (**g**, **h**), sporozoites (**k**, **l**), oocysts (**i**, **j**) and exo-erythrocytic meronts (**m**–**p**) are clearly different in size. A large conspicuous vacuole (**d**) is present in zygote of *H. fringillae*, which is not a case in zygote of *H. tartakovskyi* (**c**). A long outgrowth appears in apical end of developing *H. tartakovskyi* ookinete (**f**), which is not a case in *H. minutus* ookinete (**e**). Developing oocyst of *H. palumbis* (**j**) contains numerous germinative centres (**j**), which is not a case in small oocyst of *H. fringillae* (**i**). One end of *H. palumbis* sporozoite is rounded (**l**), which is not the case in *H. tartakovskyi* sporozoite (**k**). Morphology of exo-erythrocytic meronts is markedly variable in different species of *Haemoproteus* (**m**–**p**). These examples show that different *Haemoproteus* and other haemosporidian species can be distinguished due to many characters of sporogonic and exo-erythrocytic stages, which remain unexplored in taxonomy. Long simple arrows—capsular-like wall of megalomeronts. Short simple arrows—parasite nuclei. Simple arrowheads—pigment granules. Simple wide arrowheads—apical end of ookinetes. Simple wide short arrows—vacuoles. Simple small arrowhead—merozoites. Simple wide long arrows—megalomeront cytomeres. Triangle wide short arrows—oocysts. Scale bars 10 µm (**a**–**i**, **k**, **l**), 50 µm (**j**, **m**–**p**). Other explanations are given in the text

The application of molecular diagnostic tools provides new opportunities to distinguish haemosporidian parasites using their unique DNA sequences [38]. This stimulates biodiversity studies of wildlife haemosporidians, particularly because the molecular markers make the research readily repeatable due to DNA sequence information. Additionally, parasite species detection becomes possible at all stages of the life cycle, including the relatively insubstantially studied sporogonic and tissue stages, which are still difficult to distinguish to species and even the subgeneric or generic levels using morphological characters. Due to the easy accessibility of peripheral blood of birds, the gametocytes of *Haemoproteus* parasites likely will remain the main stage used for parasite morphological identification in the nearest future. However, it is worth noting that haemosporidian species are morphologically remarkably diverse, and often are readily distinguishable at other stages of their life cycle, including gametes (Fig. 39a, b), zygotes (Fig. 39c, d), ookinetes (Fig. 39e–h), oocysts (Fig. 39i, j), sporozoites (Fig. 39k, l) and exoerythrocytic meronts (Fig. 39m–p). Morphological characters of these life cycle stages can be applied in species taxonomy, and this might reduce the number of so-called morphologically cryptic haemosporidian parasites, which are similar or non-distinguishable at the gametocyte stage [3, 7, 165]. It is interesting to note in this regard that many species of *Haemoproteus* can be distinguished due to the markedly different size of their mature ookinetes (Fig. 39g, h) and the rate of their development, which also can be considered taxonomic characters [3, 149]. For example, the fully grown ookinetes of *Haemoproteus minutus*, *H. pallidus* and *H. asymmetricus* usually do not exceed 12 µm on average in length, and they complete development rapidly (within 1 h at 18–20 °C) in vitro, but the ookinetes of *H. tartakovskyi*, *H. fringillae* and *H. balmorali* are at least 1/3 bigger and develop 3-times more slowly at the same conditions [3, 63].

Numerous morphologically indistinguishable *Haemoproteus* species have been described and are currently considered as synonyms of valid parasite names [3, 31, 32]. The lists of synonymous *Haemoproteus* species names as well as the names of unknown taxonomic position (*incertae sedis*), the names of species of doubtful identity (*species inquirenda*), were justified and are available in [3]; these data did not change significantly and were not repeated in this review. Most of these parasite descriptions were incomplete and came from old studies published in the twentieth century. Some new names of doubtful identity were reported in keys. Because of the high genetic diversity of avian *Haemoproteus* parasites and their relatively high vertebrate host specificity, some of the available synonymous names might be considered valid in the future. In other words, such names might be a reserve for future nomenclature studies [198]. Further morphological research combined with molecular parasite characterization are needed to prove the validity of available synonyms [3]. For taxonomic clearance, invalid species names (*nomen nudum*) of haemosporidian parasites should be excluded from taxonomic use [198, 199]. The *Haemoproteus* parasites belonging to categories of *incertae sedis* and *species inquirenda* [3, 31, 32] will require further investigation before their final taxonomic status is determined.

It should be mentioned that GenBank contains DNA sequences of haemosporidian parasites, where the molecular characterization was developed incorrectly or might be questionable [200, 201]. Parasite species identification and molecular characterization is the responsibility of researchers who deposit the sequence information in GenBank. With regard to *Haemoproteus* species, some such insufficiently dependable molecular characterizations are mentioned in the footnotes of Table 36. Selection of the sequences for phylogenetic analysis and related molecular research requires targeting efforts and the MalAvi database is a helpful resource of information for this purpose [13].

## Conclusion

*Haemoproteus* is a sister genus to malaria parasites of genus *Plasmodium*. The parasites of both genera are common in birds globally, however, *Haemoproteus* species remain neglected. This is unfortunate because knowledge about close relatives of malaria pathogens is essential for understanding the evolutionary history of malaria and also the entire group of haemosporidian infections, which are flourishing in wildlife. Recent histopathological findings show that *Haemoproteus* parasites can damage bird organs during exo-erythrocytic development and thus can contribute to the overall bird health. However, these avian pathogens remain insufficiently studied partly due to difficulties in their species identification, which is an obstacle for comprehensive biodiversity research. This study developed illustrated dichotomous keys for the identification of described avian *Haemoproteus* species allowing their recognition using morphological features of blood stages (gametocytes). The most taxonomically valuable morphological characters of gametocytes and their host cells were systemized and used in the keys. In all, 177 species of *Haemoproteus* species parasitizing birds can be identified using the morphological characters of blood stages. The DNA sequences (taxonomic barcodes) are available for approximately 42% of them, and can simplify the identification of these parasites. The easy-to-use keys should be helpful not only for identification of described parasites, but also for distinguishing new pathogens, which still need discovery and description. It worth noting that species of *Haemoproteus* are remarkably morphologically diverse not only on gametocyte stage, but also on the exo-erythrocytic and vector stages, which remain insufficiently investigated and remain as unexplored reserves for future taxonomic and biodiversity studies. The majority of described *Haemoproteus* parasites also remain non-characterized using molecular markers; their development is an important task for current haemosporidian parasite researchers. This is particularly true because such markers are invaluable for haemosporidian parasite species identification in the sporogonic and tissue stages, which remain unassessed in most of these wildlife pathogens.

## Data Availability

All data generated during this study are included in this published article.
